# Recent Progress in Synthetic Applications of Hypervalent Iodine(III) Reagents

**DOI:** 10.1021/acs.chemrev.4c00303

**Published:** 2024-09-13

**Authors:** Akira Yoshimura, Viktor V. Zhdankin

**Affiliations:** † Faculty of Pharmaceutical Sciences, Aomori University, 2-3-1 Kobata, Aomori 030-0943, Japan; ‡ Department of Chemistry and Biochemistry, University of Minnesota Duluth, Duluth, Minnesota 55812, United States

## Abstract

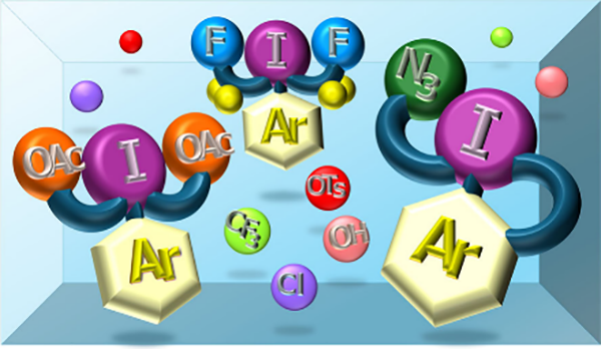

Hypervalent iodine(III) compounds have found wide application in modern organic chemistry as environmentally friendly reagents and catalysts. Hypervalent iodine reagents are commonly used in synthetically important halogenations, oxidations, aminations, heterocyclizations, and various oxidative functionalizations of organic substrates. Iodonium salts are important arylating reagents, while iodonium ylides and imides are excellent carbene and nitrene precursors. Various derivatives of benziodoxoles, such as azidobenziodoxoles, trifluoromethylbenziodoxoles, alkynylbenziodoxoles, and alkenylbenziodoxoles have found wide application as group transfer reagents in the presence of transition metal catalysts, under metal-free conditions, or using photocatalysts under photoirradiation conditions. Development of hypervalent iodine catalytic systems and discovery of highly enantioselective reactions using chiral hypervalent iodine compounds represent a particularly important recent achievement in the field of hypervalent iodine chemistry. Chemical transformations promoted by hypervalent iodine in many cases are unique and cannot be performed by using any other common, non-iodine-based reagent. This review covers literature published mainly in the last 7–8 years, between 2016 and 2024.

## 1 Introduction

Compounds of polyvalent iodine, commonly known as hypervalent iodine reagents, have found wide application in modern organic synthesis as reagents and catalysts. Many features of hypervalent iodine chemistry are similar to the transition metals chemistry, and further exploration of these similarities has led to the creation of new reagents and important synthetic methodologies.^1^ In contrast to heavy transition metals, iodine is an environmentally friendly and a relatively inexpensive element (according to the U.S. Geological Survey bulk average price of iodine in the United States from 2017 to 2023 was between $19 and $61 per kilogram, see https://www.statista.com/statistics/1001991/average-price-iodine-us/). Many “metal-free” synthetic applications of hypervalent iodine chemistry are based on the unique oxidizing properties and commercial availability of polyvalent iodine compounds. Compounds of hypervalent iodine are widely utilized in synthetically important oxidations, halogenations, aminations, and many other oxidative transformations of organic substrates. Iodonium salts are important arylating reagents, and iodonium ylides and imides are excellent carbene and nitrene precursors. Particularly important are derivatives of benziodoxoles, such as trifluoromethylbenziodoxoles, alkynylbenziodoxoles, alkenylbenziodoxoles, and azidobenziodoxoles, which are used as group transfer reagents under metal-free conditions, or under transition metal catalysis, or using photocatalysts under photoirradiation conditions. It is important to emphasize that in many cases the reactions of hypervalent iodine compounds are unique and cannot be performed by using any other common, non-iodine-based reagent.

The important role of polyvalent iodine in modern chemistry is confirmed by a large number of recently published books and reviews covering several hot areas of hypervalent iodine chemistry. Just in the last 6 years, between 2016 and 2023, three books^2−4^ and over a hundred major reviews covering different areas of hypervalent iodine chemistry have been published.^5−131^ Several newly developed synthetic methodologies are based on the similarities between chemistry of hypervalent iodine and the chemistry of transition metals. In particular, hypervalent iodine(III) reagents and catalysts can effectively promote numerous coupling reactions, leading to the formation of new carbon–carbon and carbon–element bonds.^132^ The use of hypervalent iodine(III) compounds for the creation of new carbon–carbon bonds was summarized in several reviews.^74,79,80,133^ Numerous recent reviews were dedicated to the reactions of hypervalent iodine compounds, resulting in the formation of new carbon–nitrogen bonds.^8,15,44,55,75,81−85,134^ Construction of C–O,^135^ C–S, and C–Se bonds mediated by hypervalent iodine reagents under metal-free conditions was also reviewed.^136^ Development of enantioselective catalytic systems based on the unique redox and photoredox properties of iodine(III) has been an important direction in the field of hypervalent iodine chemistry summarized in numerous specialized reviews.^12,26,28,40,41,43,45,53,65,93−95,137,138^

Application of hypervalent iodine reagents in organic synthesis has been an active area of research.^139^ Numerous recent reviews were dedicated to applications of hypervalent iodine in the following reactions: functionalization of alkenes,^117,118,140^ direct C–H bond functionalization,^10,66,141^ chlorinations and fluorinations,^30,69,72,86,87,142−145^ phenolic dearomatization reactions,^47,56,63,121,122,146^ rearrangements promoted by hypervalent iodine,^25,67,123,147,148^ synthesis of spiroheterocycles,^119^ applications of hypervalent iodine(III) reagents in organophosphorus chemistry,^124^ reactions of hypervalent iodine with boronic compounds,^149^ reactions of hypervalent iodine with enol and ynol surrogates,^150^ hypervalent iodine compounds in transition metal chemistry,^21,125^ copper-catalyzed hypervalent iodine-mediated functionalization of unactivated compounds,^151^ palladium-catalyzed organic reactions involving hypervalent iodine reagents,^152^ organocatalytic group transfer from hypervalent iodine species,^120^ glycosylation reactions,^153,154^ functionalization of carbohydrates,^131^ tandem reactions that allow for incorporating the aryl motif into the products through a subsequent one-pot nucleophilic addition or catalytic coupling reaction,^155^ and late-stage peptide and protein functionalization.^22^ Iodine(III) compounds are commonly utilized for the preparation of various heterocycles via oxidative formation of C–C, C–O, C–N, C–S, N–O, N–N, or N–S bonds.^24,48,50,62,68,70,73,88−91,116,156−158^ Hypervalent iodine reagents are broadly used in total synthesis of natural products.^11,42,58,61,92,159^

Numerous reviews have summarized recent synthetic applications of different classes of polyvalent iodine compounds. In particular, aryliodonium salts are important arylating reagents^6,32,60,96−101,160−167^ and precursors for Positron Emission Tomography.^102,168^ Iodonium ylides are used as carbene precursors and also as reagents for radiofluorination.^103,104,169^ (Diacetoxyiodo)benzene is an important reagent with various synthetic applications.^170^ Numerous recent reviews were dedicated to the application of group-transfer benziodoxole-based reagents.^7,105−107,109,171^ Trifluoromethylbenziodoxoles are important trifluoromethylating reagents.^27,108^ Azidobenziodoxoles are exceptionally effective reagents for direct azidation of C–H bonds.^5,19,111^ Ethynylbenziodoxoles and vinylbenziodoxoles are useful alkynylating and alkenylating reagents.^13,110,130,172^ Weiss’ reagent, [PhI(Pyr)_2_]^2+^ 2TfO^–^, is a synthetically useful oxidizing reagent.^173^ Radical and photochemical reactions of benziodoxole derivatives is a hot area of modern hypervalent iodine chemistry.^14,112,113,174^ Applications of in situ generated hypervalent iodine reagents in organic synthesis have recently been reviewed.^175^

Synthetic applications of several new classes of hypervalent iodine compounds have recently been reviewed, such as pseudocyclic hypervalent iodine reagents,^114^ benziodoxole sulfonates,^51,78^ water-soluble hypervalent iodine reagents and their reactions in aqueous medium,^115,176,177^ and recyclable hypervalent iodine reagents.^20^

New methods for generating derivatives of polyvalent iodine using green chemistry methods were recently reviewed. The anodic oxidation of aryl iodides provides an efficient approach to hypervalent iodine derivatives. Electrochemically generated hypervalent iodine species have been utilized as in-cell or ex-cell mediators for valuable fluorinations and oxidative transformations.^36,37,126,178−181^ Another environmentally sustainable procedure, the aerobic preparation of hypervalent iodine reagents, is based on the oxidation of iodoarenes by the reactive intermediates generated during autoxidation of aldehydes.^127^

Iodonium salts have found some industrial application as initiators of polymerization.^46,128,129^ Hypervalent organic polyiodides are used in disinfectants, polarizing films, dye-sensitized solar cells, and precursors to low-density graphitic films.^182^ It can be expected that the interest in industrial applications of hypervalent iodine compounds will grow in the future.

The present review provides an update of our 2016 comprehensive review.^183^ The review is limited to synthetic applications of iodine(III) reagents excluding the chemistry of iodine(V). Recent publications in the area of iodine(V) chemistry are mainly represented by numerous papers on routine oxidations of alcohol groups in complex molecules by Dess-Martin periodinate or 2-iodoxybenzoic acid, which is a traditional and well-reviewed area of organic synthesis.^29,31,57,184^ Our present review covers literature on organoiodine(III) compounds published mainly in the last 5–7 years, through spring 2024.

## 2 Structure and Reactivity of Hypervalent Iodine(III) Compounds

Because of the large size of the iodine atom, the bonding in compounds of polyvalent iodine is different from the light main-group elements. Computational studies indicate that the typical light p-block elements double bonds formed by the interatomic π-bonding do not exist in the derivatives of polyvalent iodine.^185^ Structure and reactivity of polyvalent iodine compounds is usually explained by the presence of a hypervalent bond, a linear, three-center-four-electron bond L—I—L formed by the overlap of the 5p orbital on iodine atom with the orbitals on the ligands L. Hypervalent bond is highly polarized, longer, and weaker compared to a regular covalent bond, resulting in special structural features and reactivity typical of the derivatives of polyvalent iodine. Reactions of hypervalent iodine compounds are commonly discussed in terms of oxidative addition, ligand exchange, reductive elimination, and ligand coupling, which are typical of the transition metal chemistry.^1−3^ General aspects of structure and reactivity of hypervalent iodine compounds have been discussed in our 2016 review.^183^ In summary, the iodine(III) organic derivatives ArIX_2_ have trigonal bipyramidal geometry with electronegative ligands X in the axial positions, and the aryl substituent and two unshared electronic pairs occupying the equatorial positions. Iodonium salts R_2_IX with inclusion of a weakly bonded anionic part X of the molecule have a similar distorted trigonal bipyramidal structure. The hypervalent I–X bond length in iodine(III) compounds is longer than an average I–X covalent bond length in compounds of monovalent iodine. Bond angles R–I–R in iodonium salts and iodonium ylides, as well as bond angles R–I–N in iodonium imides, are close to 90°.

Several structural studies of hypervalent iodine compounds were recently published. Dutton and co-workers have reported numerous studies on structural verification of several common hypervalent iodine reagents.^186−190^ In particular, the structure of Stang’s reagent, PhI(CN)OTf, was confirmed by X-ray crystallography and determined to be best described as an ion-pair in organic solution.^186^ The structure of a complex of PhICl_2_ with pyridine was established by X-ray, NMR, and theoretical studies.^188^ Reinvestigation of the structure of PhI(OTf)_2_ has demonstrated the actual identity of the reagent being used was PhI(OTf)(OAc).^191^ While PhI(OTf)_2_ is unstable, the *para*-nitro-substituted derivative, 4-O_2_NC_6_H_4_I(OTf)_2_, was isolated as a stable compound and characterized by X-ray diffraction.^189^ The previously proposed molecule of PhIBr_2_ was demonstrated to be a mixture of PhI and Br_2_ on the basis of spectroscopical and computational studies.^190^ Waser and co-workers have reviewed X-ray and NMR structural data of ethynylbenziodoxolones (EBXs) reagents and their analogues.^192^

Reactivity of hypervalent iodine compounds in radical reactions under photochemical conditions was summarized in recent reviews.^14,40^ Numerous recent publications were dedicated to the effects of halogen bonding and Lewis acidity on structure and reactivity of hypervalent iodine compounds.^34,193−200^

Theoretical studies of the mechanisms of catalytic and stoichiometric iodine-mediated reactions have been discussed in numerous recent publications.^193,201−220^

## 3 Acyclic Iodine(III) Compounds as Reagents

### 3.1 Iodine(III) Compounds with Halide Ligands

#### 3.1.1 (Difluoroiodo)arenes

**Scheme 1:**
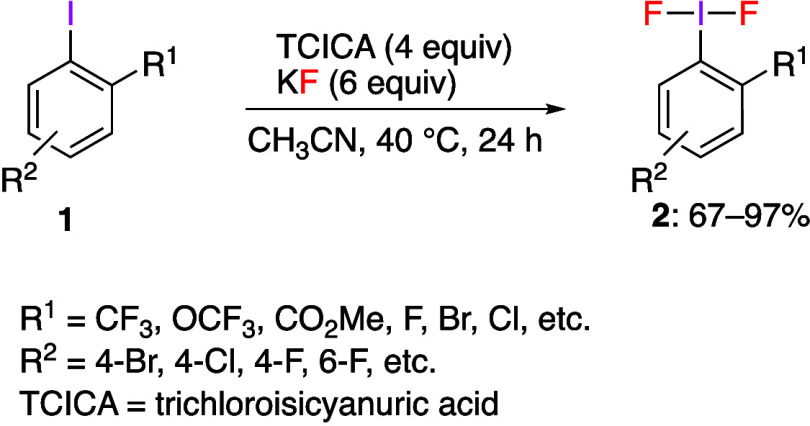
Preparation of (Difluoroiodo)arenes **2** with Ortho Substitution

The chemistry and synthetic applications of hypervalent iodine fluorides have been extensively reviewed previously^221−224^ and summarized in numerous recent reviews.^30,69,72,86,87^ Two general methods are known for the synthesis of (difluoroiodo)arenes. The first method involves the fluorination of an iodoarene by a fluorinating reagent or a combination of a common oxidant with a fluoride source. The second method is based on the ligand exchange between a hypervalent iodine compound and a fluoride source. These two approaches have been summarized in numerous previous publications.^225−228^ In the following text, several recently published synthetic procedures for converting iodoarenes to the corresponding (difluoroiodo)arenes are described. Gilmour’s group^228^ has published an improved procedure for the synthesis of (difluoroiodo)arenes that was originally reported by Shreeve’s group in 2005.^228,229^ In the improved procedure, *para*-iodotoluene is mixed with Selectfluor and CsF as an additional fluoride source to give *para*-(difluoroiodo)toluene in 62% yield. Using Et_3_N·HF instead of CsF improved the yield up to 92% yield; however, Et_3_N·HF is a potentially hazardous reagent. Monitoring this reaction by NMR indicated poor conversion after 48 h for iodoarenes with a strong electron-withdrawing groups such as nitro- and trifluoromethyl group, while the conversion was good for iodoarene with electron-donating groups. Murphy’s group reported a large scale procedure for the preparation of *para*-(difluoroiodo)toluene up to 50 mmol.^226^ This procedure involves three steps to prepare *para*-(difluoroiodo)toluene starting from 4-iodotoluene in 64–72% yield without purification of intermediate products in the first and second steps. Togni’s group developed direct synthesis of (difluoroiodo)arenes from iodoarenes with various electron-withdrawing groups in the *ortho*-position.^227^ It has been noted that the *ortho*-substituent provides a minor improvement of hydrolytic stability of the obtained (difluoroiodo)arenes and it plays an important role during the reaction with TCICA (trichloroisicyanuric acid) and KF, inhibiting further oxidation of iodine(III) to iodine(V). The reaction of iodoarene **1** and TCICA with KF as the fluoride source affords the corresponding products **2** in good yields (Scheme 1). The authors suggest that the introduction of an electron-withdrawing group at the *ortho*-position suppresses overoxidation of the produced iodine(III) product to an iodine(V) compound. This methodology provides a mild and efficient approach to electron-deficient (difluoroiodo)arenes and complements the methods of Shreeve’s group^229^ and Gilmour’s group^228^ which utilize Selectfluor to synthesize electron-rich (difluoroiodo)arenes.

**Scheme 2:**
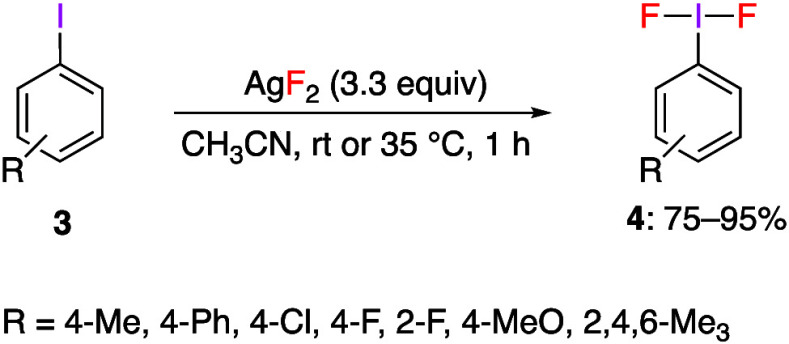
Preparation of (Difluoroiodo)arenes **4** Using AgF_2_

**Scheme 3:**
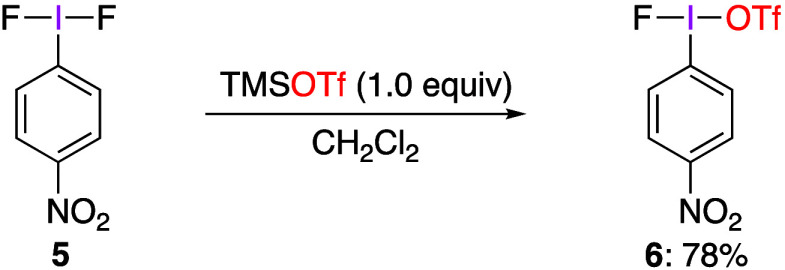
Preparation of *p*-NO_2_C_6_H_4_IF(OTf) **6**

In 2022, Zhang and co-workers reported a one-step synthesis of (difluoroiodo)arenes using silver difluoride (AgF_2_) as fluorinating reagent.^225^ The reaction of iodoarene **3** with AgF_2_ is carried out by stirring at room temperature for 1 h to give (difluoroiodo)arenes **4** in good yileds (Scheme 2). In the reaction of 2,4,6-trimethyliodobenzene, slight heating was necessary to give the respective product in 82% yield. A moderately unstable product **4** with methoxy group in the *para*-position was obtained in 79% yield.

(Difluoroiodo)arenes can participate in ligand exchange reactions to produce the corresponding products.^230−232^ Dutton and co-workers used the ligand exchange reaction between *p*-NO_2_C_6_H_4_IF_2_**5** and TMSOTf to synthesize *p*-NO_2_C_6_H_4_IF(OTf) **6** (Scheme 3), the structure of which was characterized by X-ray crystallography.^230^ Previously, the formation of PhIF(OTf) in situ from iodobenzene and xenon fluorotriflate −78 °C was proposed by Stang, Zefirov, and co-workers without any structural proof based on the formation of iodonium salts in the reactions with alkynes.^233^ Dutton and co-workers have found that the presence of the nitro group stabilizes compound **6** by preventing degradation via an electrophilic aromatic substitution pathway observed in previous studies of ArI(OTf)_2_ species.^189^ The authors also found that compound **6** reacts with anisole and toluene to give the corresponding diaryiodonium triflates, but it does not react with bromobenzene.

**Scheme 4:**
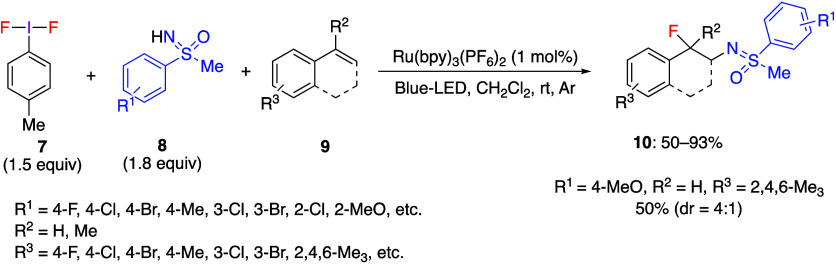
Fluoro Sulfoximidations of Styrenes **9**

Bolm and co-workers have reported that *p*-TolIF_2_ reacts with NH-sulfoximines, leading to the formation of unstable ligand exchange species as confirmed by ^1^H NMR, ^19^F NMR, and ESI-MS.^232^ These species were utilized in situ in subsequent reactions with alkenes. The reaction of *p*-TolIF_2_**7** with various NH-sulfoximines **8** and styrenes **9** under Blue-LED with Ru photoredox catalyst gave the corresponding fluoro sulfoximination products **10** in moderate to good yields (Scheme 4). The same authors also reported that when the active species generated by ligand exchange between *p*-TolIF_2_**7** and NH-sulfoximine **8** were stirred in dichloromethane or dibromomethane under Blue-LED without Ru photoredox catalyst conditions, the corresponding chlorinated or brominated products instead of the fluorinated products **10** were formed.^231^ A radical mechanism was proposed for these reactions because the products were not formed under dark conditions or in the presence of the radical scavenger such as TEMPO or BHT.

**Scheme 5:**
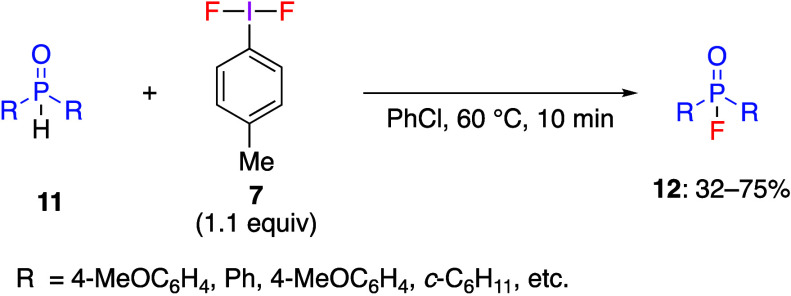
Preparation of Phosphate Fluorides **12**

**Scheme 6:**
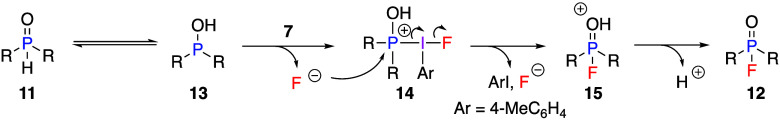
Proposed Reaction Mechanism of Fluorination of Phosphine Oxide **11**

As a useful fluorination reagent, *p*-TolIF_2_**7** has been used in direct mono- and difluorination reactions of various substrates.^87,183^ As a recent example, Balz-Scheimann fluorination of aromatic diazonium salts with *p*-TolIF_2_**7** in the presence of BF_3_·Et_2_O has been reported by Hu and co-workers.^234^ Murphy’s group developed a denitrogenative hydrogen-fluorination of benzaldehyde hydrazine derivatives with electron-withdrawing group.^235^ The same research group has demonstrated that the reaction of *p*-TolIF_2_**7** with diaryl- or dialkyl phosphine oxides **11** leads to the corresponding phosphate fluorides **12** (Scheme 5),^236^ and the reaction with some substrates yields phosphinic acid as a by-product. In the reaction with diethylphosphite, the desired product was obtained in only 34% yield. Although some products **12** are unstable, they can be effectively converted to stable phosphinate esters by treatment with ethanol and isolated. The authors proposed a reaction mechanism involving initial nucleophilic addition of phosphine **13** to the electrophilic iodane **7**, forming phosphonium adduct **14**. Subsequent nucleophilic attack by fluoride anion on the phosphonium atom in adduct **14** gives intermediate **15**, whose deprotonation affords the final phosphoric fluoride **12** (Scheme 6).

**Scheme 7:**
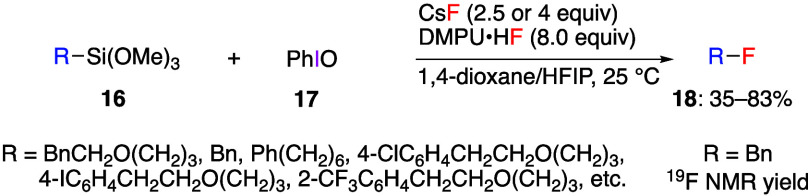
Fluorination Reaction of Alkylsilanes **16**

Monofluorination reactions using ArIF_2_ in situ, in which a fluoride source is added to iodosylbenzene, DIB, or PIFA to form ArIF_2_ in the reaction system, are also known.^237,238^ Xu and Tang reported that the reaction of alkylsilanes **16** with iodosylbenzene **17** using two fluoride sources, 1,3-dimethyl-3,4,5,6-tetrahydro-2(1*H*)-pyrimidone hydrofluoride (DMPU·HF) and CsF, gave the corresponding alkyl fluorides **18** (Scheme 7).^238^ Using this procedure, a variety of primary alkylsilanes **16** bearing electron-donating or electron-withdrawing substituents in aromatic ring and not-aryl-substituted alkylsilanes **16** were converted to products **18** in good yields. In the reactions of secondary alkylsilanes no products **18** were observed, and the major by-products were alkenes and ketones. The reaction could be carried out on the gram scale. For the mechanism, the results of a radical clock reaction and the addition of TEMPO indicate that radical species are involved. In the presence of CsF and DMPU·HF, PhIF_2_ is generated from alkylsilane and PhIO with organopentafluorosilicate as the by-product.

**Scheme 8:**
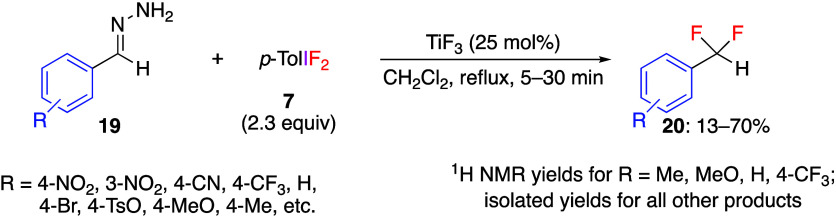
*gem*-Difluorination Reaction of Hydrazones **19**

Substrates bearing diazo or benzaldehyde hydrazone moiety react with *p*-TolIF_2_**7**, producing products of difluorination.^239−241^ For example, Murphy and co-workers demonstrated that the reaction of benzaldehyde hydrazone **19** with *p*-TolIF_2_**7** and a catalytic amount of TiF_3_ yields the corresponding *gem*-difluorides **20** in moderate yields (Scheme 8).^239^ In the reaction without TiF_3_, the monofluoride is the main product due to insufficient activation of **7**. In this reaction, starting compounds **19** react with reagent **7** to form diazo species as the reaction intermediate, which was confirmed by ^1^H NMR.

**Scheme 9:**
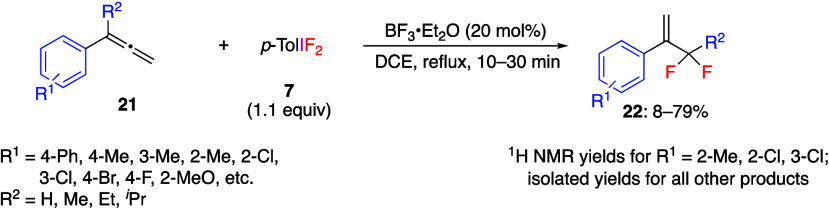
Preparation of α-(Difluoromethyl)styrenes **22**

It has been reported that the reaction of aryl-substituted allenes **21** with *p*-TolIF_2_**7** in the presence of a catalytic amount of BF_3_ yields α-(difluoromethyl)styrenes **22** via 1,2-aryl group shift (Scheme 9).^242^ The reactions of *para*-substituted substrates **21** work best, while the yields of *ortho*- and *meta*-substituted derivatives are generally lower. The reaction also proceeds well with allene compounds having an alkyl substituent at the α-position, resulting in the desired products in moderate yields. Futhermore, the reaction was confirmed to proceed under similar conditions for 1-naphthyl and 2-naphthylallenes. To identify which double bond of the allene is involved in this reaction, the authors tested the allene compound with deuterated terminal hydrogens and found that this reaction produced α-difluoromethylstyrene with deuterated hydrogens at the β-position. This result confirmed that no terminal double bond was involved in this reaction. When exocyclic allenes are used as substrates, the ring expansion reaction proceeds and the cyclic products are obtained in moderate yields.^243^

**Scheme 10:**
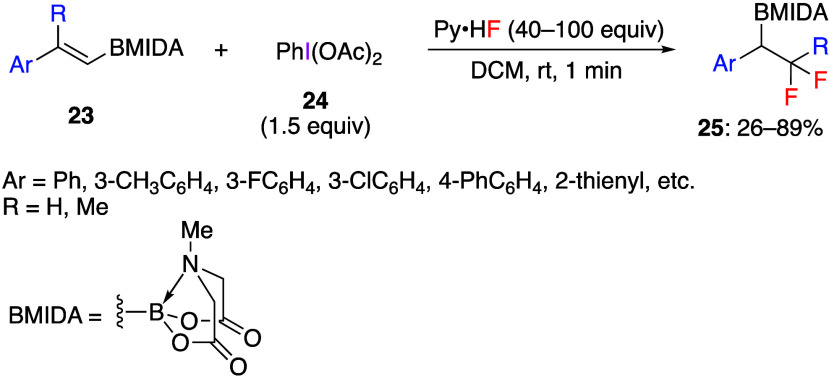
Synthesis of β-Difluorinated Alkylborons **25** from Styrenes **23**

**Scheme 11:**
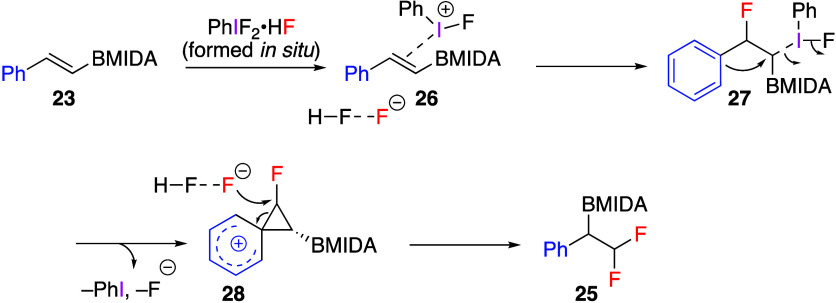
Proposed Mechanism of Formation of Products **25** from Styrenes **23**

The synthesis of difluorinated compounds from styrenes via 1,2-phenyl group shift reaction using (difluoroiodo)benzene generated by adding a fluoride source to DIB or PhIO was also reported.^244^ For example, Wang and co-workers reported that the reaction of aryl-substituted alkenyl *N*-methyliminodiacetyl (MIDA) boronates **23** with DIB **24** and HF·Py gave β-difluorinated alkylborons **25** in low to good yields (Scheme 10).^245^ When 1,1-disubstituted alkenyl MIDA boronates were used as substrates, α-difluorinated alkylborons were successfully obtained. The authors prepared a deuterated substrate and based on the analysis of products found that 1,2-aryl shift occurs during the reaction. The proposed reaction mechanism starts from the interaction between the double bond of the substrate **23** and (difluoroiodo)benzene generated from PIDA and Py·HF, leading to regioselective vicinal fluoroiodination intermediate **27** via species **26** (Scheme 11). The observed regioselectivity is explained by the ability of the aryl group to stabilize the developing benzylic carbocation. At the next step, departure of the PhI leaving group affords phenonium ion species **28**. Finally, the regioselective fluoride attack results in the ring-opening of **28** forming final products **25**.

**Scheme 12:**
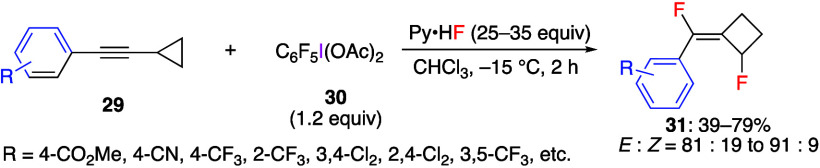
Preparation of Difluorinated Alkylidenecyclobutanes **31**

**Scheme 13:**
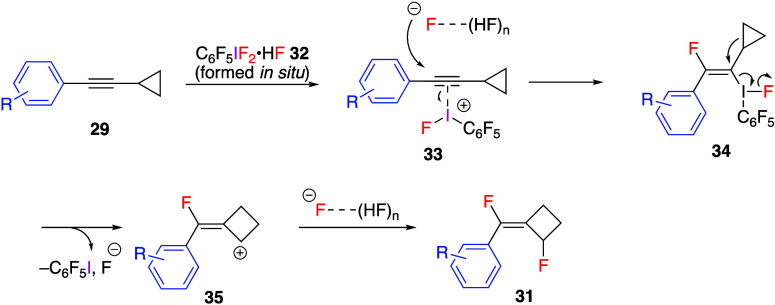
Proposed Mechanism of Formation of Difluorinated Alkylidenecyclobutanes **31**

Difluorination reactions with alkynes using (difluoroiodo)arene generated in the reaction system were reported by Li’s group.^246^ They reported that the reaction of aryl alkynylcyclopropanes **29** with pentafluoro(diacetoxyiodo)benzene **30** and an excess amount of HF·Py gave the ring-expanded difluoroalkylidene cyclobutane compounds **31** with (*E*)-stereoselectivity (Scheme 12). The reaction proceeded efficiently with electron-poor phenyl-substituted alkynylcyclopropanes as substrates but was complicated for the electron-rich aryl-substituted and alkyl-substituted alkynylcyclopropanes, presumably because of the competing oxidation of the substrate under reaction conditions. The proposed mechanism of this reaction involves the initial ligand exchange of compound **30** with HF·Py giving activated iodoarene difluoride **32**, which then interacts with the triple bond of substrates **29** to form π-complex **33**. At the next step, the regio- and stereoselective fluoride attack on π-complex **33** affords intermediate **34**, which undergoes a Wagner–Meerwein-type rearrangement to form cyclobutyl cation **35**. Finally, the trapping of this cation by second fluoride leads to stereoselective formation of products **31** (Scheme 13).

Variuos (difluoroiodo)arenes, ArIF_2_, can be generated from the corresponding iodoarenes in situ and further reacted with organic substrates using electrochemical or chemical oxidation in the presence of fluoride anions.^247,248^

**Scheme 14:**
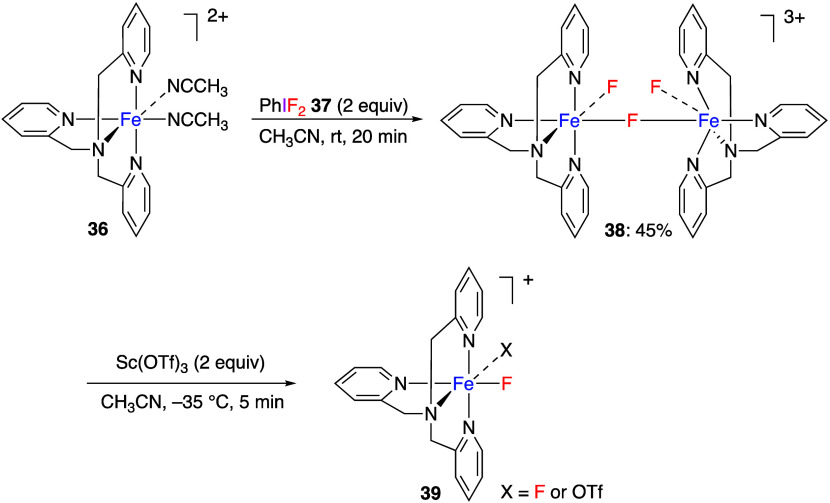
Synthesis of μ-Fluorido-Diiron(III) Complex **38** and Monomeric Product **39**

The McDonald group found that iron(II) complex **36** bearing tris(2-pyridylmethyl)amine react with (difluoroiodo)benzene **37** at room temperature in acetonitrile to form μ-fluorido-diiron(III) complex **38** (Scheme 14).^249^ The structure of the obtained compound, which is bridged by fluorine atoms, was determined by X-ray structural analysis. Furthermore, the addition of Lewis acid Sc(OTf)_3_ to compound **38** resulted in the formation of a monomeric product **39**. The same group also found that the reaction of iron(II) complex with tris(2-benzimidazoylmethyl)amine and (difluoroiodo)benzene formed the analogous μ-fluorido-diiron(III) complex.^250^

**Scheme 15:**
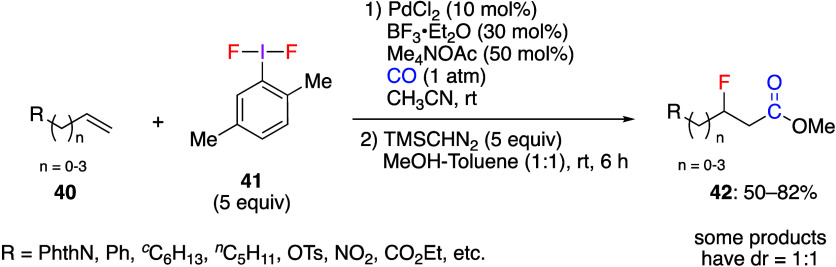
Fluorocarbonylation of Unactivated Alkenes **40**

Liu and co-workers have reported the palladium-catalyzed oxidative fluorocarbonylation of unactivated alkenes **40** using 2,5-(CH_3_)_2_C_6_H_3_IF_2_**41** (Scheme 15).^251^ The reaction is applicable to a variety of alkenes **40**, and the addition of MeOH and TMSCHN_2_ during the reaction yields the corresponding β-fluoro carboxylic esters **42**. When H_2_O or amine were used instead of MeOH and TMSCHN_2_, β-fluoro carboxylic acids and amides were obtained.

Additional recent examples of synthetic applications of (difluoroiodo)arenes include the following works: fluorination of *cis*-1,4-polyisoprene,^252^*para*-selective benzylation of aryl iodides by the in situ preparation of ArIF_2_,^247^ oxidative fluoroarylation of benzylidenecyclopropanes by in situ generated ArIF_2_,^253^ and hypervalent iodine-mediated gem-difluorination of vinyl halides by in situ generated ArIF_2_.^254^

#### 3.1.2 (Dichloroiodo)arenes

From an historical point of view, (dichloroiodo)benzene is one of the first reported hypervalent iodine compounds that has been intensively used as a convenient chlorinating reagent for over a century.^221−224^ The chemistry and synthetic applications of hypervalent iodine chlorides have recently been reviewed.^30,86^ Numerous synthetic approaches have been developed to (dichloroiodo)arenes, which are the oldest known hypervalent iodine compounds with many practical applications.^255^ The classic general approach to (dichloroiodo)arenes is based on reactions of iodoarenes with chlorine gas or chlorine-derived oxidants.^256^ Alternatively, (dichloroiodo)arenes can be prepared by ligand exchange reactions of other hypervalent iodine reagents with sources of chloride anion.^257^ Ligand exchange reactions of (dichloroiodo)arenes leading to other hypervalent iodine reagents are also known.^230,258^ In recent years, numerous oxidations or oxidative chlorination reactions of various organic or organometallic substrates with (dichloroiodo)arenes have been reported. Computational studies of several chlorination reactions have also been performed.^211,259^

**Scheme 16:**
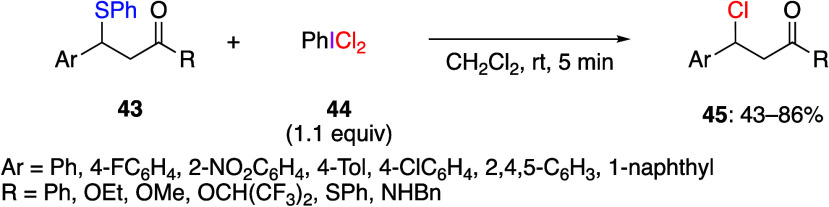
Reaction of β-Sulfidocarbonyl Compounds **43** with (Dichloroiodo)benzene **44**

Electrophilic or radical addition or substitution reactions are the most typical reactions of (dichloroiodo)arenes. Chlorinations by substitution reactions have been reported for a variety of aromatic and aliphatic substrates.^236,260−269^ Ibrahim and Adamo reported that the reaction of β-sulfidocarbonyl compounds **43** with a (dichloroiodo)benzene **44** at room temperature for a few minutes afforded the corresponding β-aryl-β-chlorocarbonyl compounds **45** in moderate to good yields (Scheme 16).^260^ This reaction is also applied to alkylphenyl sulfides as substrates. When (*S*)-β-sulfidocarbonyl compounds are used as substrates in this reaction, (*R*)-β-aryl-β-chlorocarbonyl compounds are obtained with high stereoselectivity. Based on this result, the authors suggest that the reaction mechanism starts from the oxidation of sulfur with (dichloroiodo)benzene, followed by an S_N_2-like reaction with the chloride anion to afford product **45**.

**Scheme 17:**
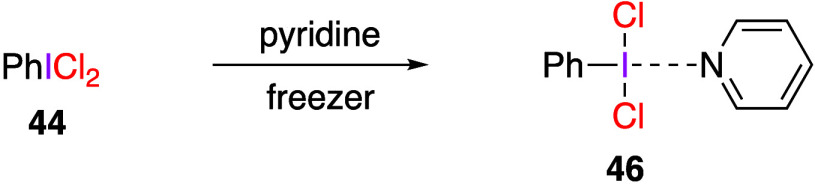
Preparation of Tetracoordinated Hypervalent Iodine Compound **46**

Dutton and co-workers reported aromatic chlorination reactions of (dichloroiodo)benzene with arenes in the presence of additives.^188,270−272^ They have found that the addition of pyridine as an additive to (dichloroiodo)benzene **44** gives the corresponding tetracoordinated hypervalent iodine compound **46** (Scheme 17), the structure of which was characterized by X-ray structural analysis.^188^ The N–I distance is 2.750 Å, which is longer than the covalent distance of a typical N–I bond. Likewise, when tetrabutylammonium chloride is used as an additive instead of pyridine, similar tetracoordinated hypervalent iodine compounds can be obtained.^271^ This group has also investigated the mechanism of this reaction using NMR and computational chemistry.

**Scheme 18:**
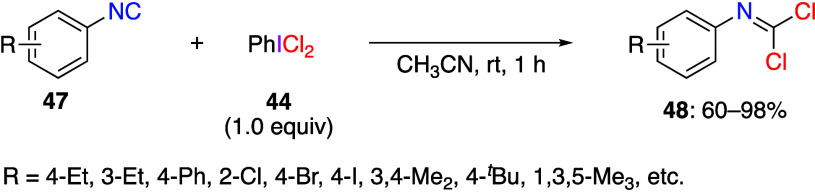
*gem*-Dichlorination of Aromatic Isonitriles **47** Using **44**

**Scheme 19:**
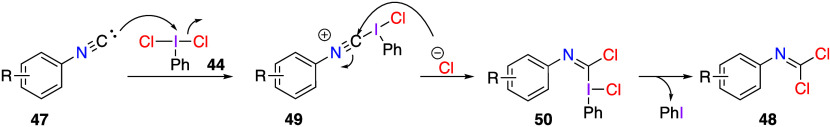
Proposed Mechanism of *gem*-Dichlorination Reaction of Aromatic Isonitriles **47**

Reactions using (dichloroiodo)arenes to add chlorine to organic substrates have also been reported. Liu and co-workers reported that the reaction of substituted aromatic isonitrile **47** with (dichloroiodo)benzene **44** in acetonitrile proceeded as *gem*-dichlorination, yielding the corresponding carbonimide dichlorides **48** in moderate to good yields (Scheme 18).^273^ In contrast, the reaction with aliphatic isonitrile did not yield the product of *gem*-dichlorination. The potential synthetic value of carbonimide dichlorides **48** is that these compounds can be used as synthetic intermediates for 2,2,3,3-tetrachloroaziridine, propyl formimidate, and benzophenone imine. The reaction begins with the hypervalent iodine center of **44** attacking the nucleophilic carbon atom of isonitrile **47** to form intermediate **49**. Subsequently, intermolecular nucleophilic substitution results in the formation of a C–Cl bond to produce intermediate **50**. Then, intermediate **50** undergoes reductive elimination from the iodine center to give the dichlorination product **48** (Scheme 19).

**Scheme 20:**
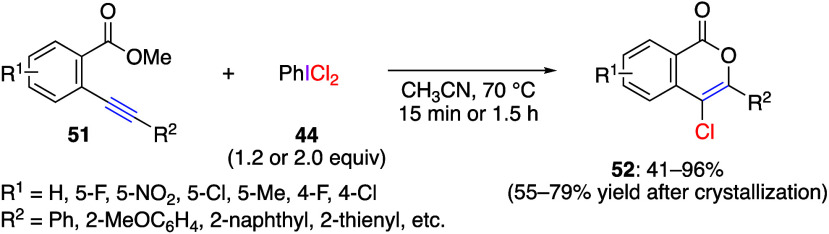
Reaction of Methyl *o*-Alkynylbenzoates **51** with Reagent **44**

**Scheme 21:**
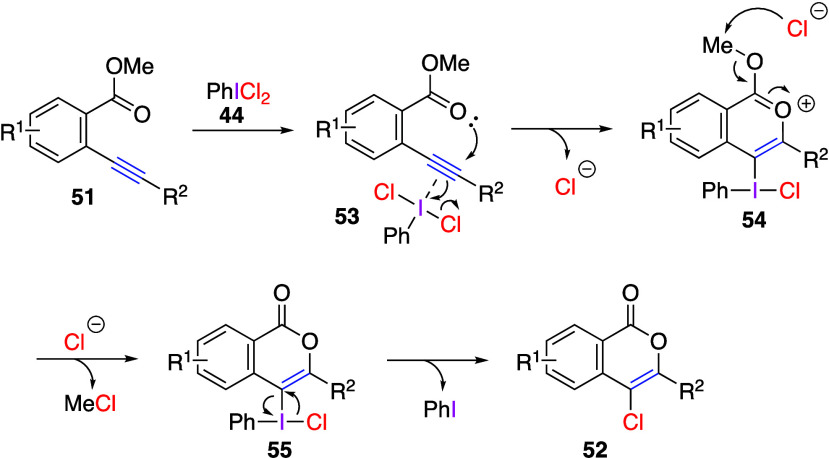
Proposed Mechanism of Chlorolactonization of Methyl *o*-Alkynylbenzoates **51**

Chlorocyclization reactions have also been reported.^259,274,275^ Du and co-workers have reported that the reaction of methyl *o*-alkynylbenzoate **51** with (dichloroiodo)benzene **44** proceeds with the formation of carbon–oxygen and carbon-chlorine bonds to produce the corresponding 4-chloroisocoumarins **52** (Scheme 20).^275^ The reaction proceeds even when ethyl or *tert*-butyl groups are used instead of methyl groups in the ester moiety of the substrate, and especially when benzyl esters are used; benzyl chloride is obtained as a byproduct, which is an important product in predicting the reaction mechanism. The proposed reaction mechanism of this chlorolactonization reaction starts from the initial coordination of PhICl_2_**44** with the alkyne triple bond to give intermediate **53**. This is followed by a concerted process involving nucleophilic attack of the carbonyl oxygen atom on the triple bond and the triple bond on the hypervalent iodine center of PhICl_2_**44**, affording cyclic oxonium ion **54**. At the next step, chloride anion nucleophilically attacks the methyl carbon center in **54**, leading to intermediate **55**. Finally, reductive elimination of iodobenzene in **55** gives the chlorination product **52** (Scheme 21). A similar chlorocyclization reaction using methyl *o*-styrylbenzoate or methyl *o*-styrylbenzamide has also been reported. In this chlorolactonization reaction, the corresponding 3,4-dihydroisocoumarins or 3,4-dihydroisocoumarin-1-imines were efficiently obtained.^274^

**Scheme 22:**
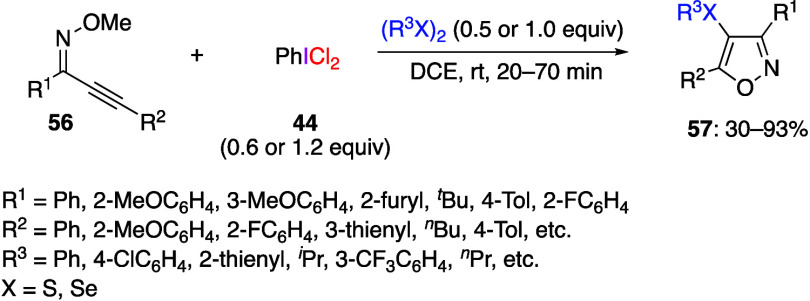
Oxidative Cyclization Reaction of Alkynone (*Z*)-*o*-Methyl Oximes **56**

It has been reported that (dichloroiodo)benzene reacts with disulfides or diselenides, forming highly reactive sulfenyl chlorides or selenenyl chlorides.^276−282^ The generated active species can then react with various substrates, leading to the corresponding sulfur or selenium products. For example, Du and co-workers reported that sulfenyl chloride or selenenenyl chloride species, generated in situ by the addition of dichloroiodobenzene **44** to a disulfide or diselenide, react with alkynone (*Z*)-*o*-methyl oximes **56**, providing the corresponding organoselanyl- or organosulfanyl-isoxazoles **57** (Scheme 22).^279^ The authors attempted to isolate the active species, selenyl chloride, and performed NMR measurements. They have also found that the isolated selenyl chloride species can be used to obtain products **57**, confirming that it is the key intermediate in this reaction. It has also been reported that when methyl *o*-alkynylbenzoate was used as a substrate under similar reaction conditions, 4-sulfenyl- or 4-selenyl-substituted isocoumarin products could be effectively obtained.^277^ Du and Zhao reported a method for generating organosulfenyl chloride species from sulfoxide and (dichloroiodo)benzene **44** and cyclization reactions using this species.^283^

**Scheme 23:**
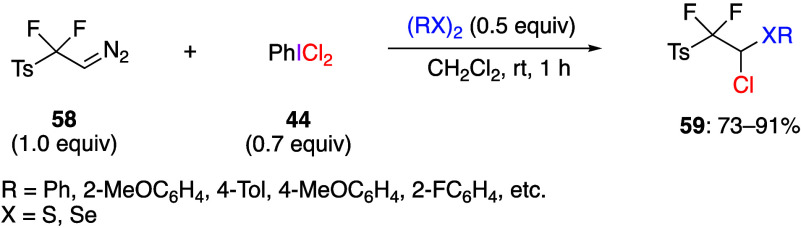
*gem*-Chlorosulfurization of *p*-Toluenesulfonyl Difluorodiazoethane **58**

Chlorosulfurization reactions as well as intramolecular oxidative cyclization reactions could also be performed using reactive sulfenyl chloride species generated from disulfide compounds and (dichloroiodo)benzene **44**.^278^ The reaction of a disulfide compound and (dichloroiodo)benzene **44** with *p*-toluenesulfonyl difluorodiazoethane **58** gives the corresponding products of chlorosulfurization **59** (Scheme 23). The reaction with diselenides instead of disulfides yields the corresponding selenium products. Instead of difluorodiazoethanes **58**, diazo compounds with perfluoroalkyl groups, α-diazophosphonates, α-diazoesters, or α-diazoketones can also be used to obtain the corresponding products in good yield.

**Scheme 24:**
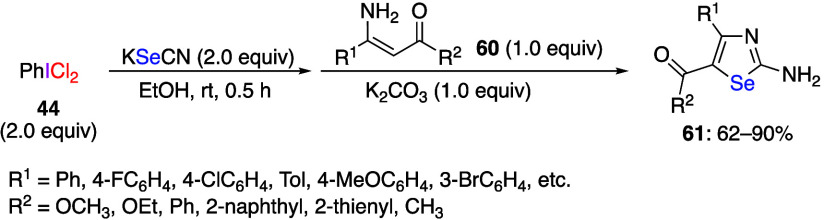
Preparation of 2-Amino-1,3-selenazoles **61**

Du and co-workers reported that the reaction of KSeCN with (dichloroiodo)benzene **44** and conjugated enamine compounds **60** allows synthesis of 2-amino-1,3-selenazole compounds **61** (Scheme 24).^284^ It was suggested that selenocyanogen (SeCN)_2_ is initially generated from (dichloroiodo)benzene and KSeCN in this reaction. The generated (SeCN)_2_ reacts with conjugated enamine compounds to give final products **61**. Another possible reaction intermediate is chloroselenocyanate, ClSeCN. However, a control reaction of ClSeCN with conjugated enamine **60** gives product **61** only in low yield, confirming that ClSeCN is not a likely intermediate in this reaction.

**Scheme 25:**
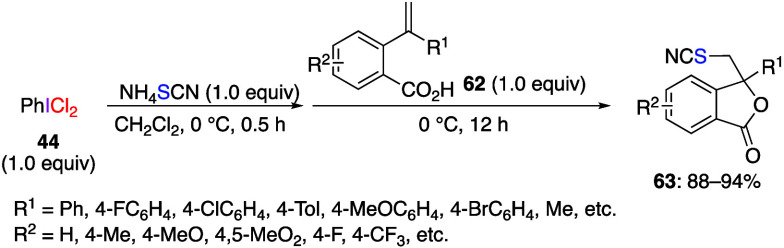
Preparation of Thiocyanated Isobenzofuranones **63**

**Scheme 26:**
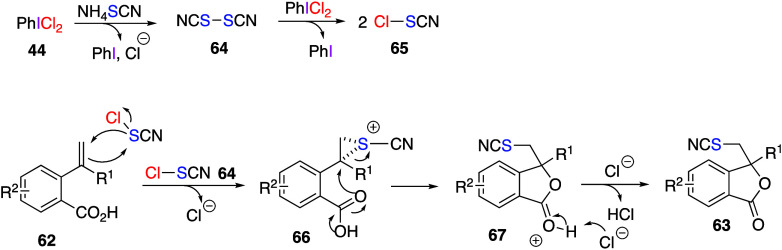
Proposed Mechanism of Formation of Thiocyanated Isobenzofuranones **63**

**Scheme 27:**
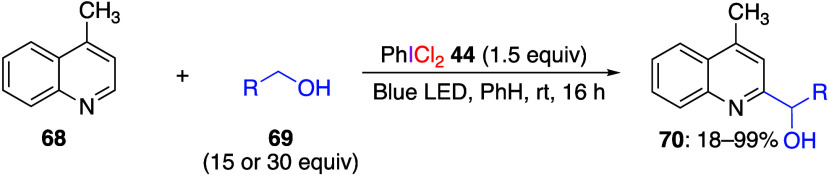
Reaction of 4-Methylquinoline **68** and Alcohols **69** with Reagent **44** under Blue-LED

**Scheme 28:**
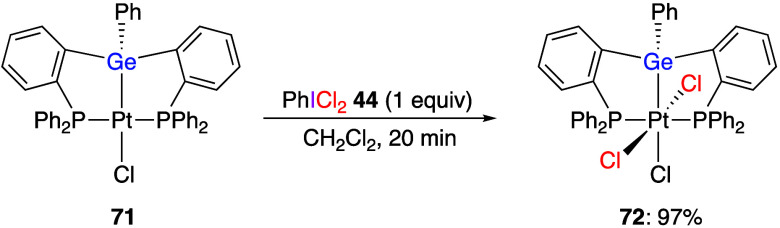
Preparation of Complex **72** using Reagent **44**

**Scheme 29:**
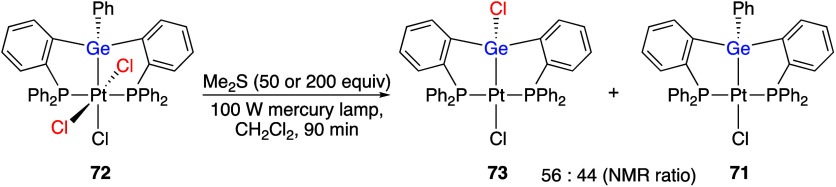
Photolysis of Complex **72**

The same group has also reported the reaction of chlorothiocyanate ClSCN generated from (dichloroiodo)benzene and NH_4_SCN.^285,286^ The reaction of *o*-alkenylbenzoic acids **62** with NH_4_SCN and (dichloroiodo)benzene **44** gave the corresponding cyanated isobenzofuranones **63** (Scheme 25).^285^ The proposed mechanism of this reaction involves consecutive generation of the reactive electrophilic species (SCN)_2_**64** and Cl-SCN **65** from PhICl_2_**44** and NH_4_SCN. Next, electrophilic addition of Cl-SCN **65** to the double bond of **62** produces thiiranium ion **66**, which is highly reactive toward nucleophilic ring opening. The carbonyl oxygen of the carboxylic acid moiety would attack the more substituted position via the S_N_1 mechanism to afford intermediate **67**. Final deprotonation of this intermediate B gives final product **63** (Scheme 26). When KSeCN was used instead of NH_4_SCN, the corresponding selenocyanated isobenzofuranones were obtained via chloroselenocyanate, ClSeCN, as active species. Similar to the reactions of KSeCN and NH_4_SCN, a reaction of tetramethylammonium trifluoromethylselenate, NH_4_SeCF_3_, with (dichloroiodo)benzene to generate CF_3_SeSeCF_3_ species has also been reported.^287^

(Dichloro)iodobenzene can also be used in the hydrogen atom abstraction (HAT) reactions. Hu and Yin reported that when 4-methylquinoline **68** and an aliphatic alcohol **69** were reacted with dichloroiodobenzene under LED irradiation, an α-heteroarylation reaction of the aliphatic alcohol occurred and the corresponding secondary alcohols **70** were obtained (Scheme 27).^288^ In the presence of radical scavengers such as TEMPO, butylated hydroxytoluene (BHT), or 1,1-diphenylethylene the reaction did not yield products **70**, indicating that radicals were involved in this reaction. The authors suggest that the radical species generated in this system participate in selective hydrogen atom abstraction from alcohols.

(Dichloroiodo)arenes have been used as oxidants of various transition metal complexes. Numerous recent works have reported reactions of (dichloroiodo)arenes with compounds of Fe,^289,290^ Ni,^291−293^ Cu,^294^ Y,^295^ Zr,^295^ Ru,^296^ Rh,^297,298^ Pd,^299−302^ Os,^303^ Ir,^304−306^ Pt,^307−317^ and Au.^318−333^ The oxidized organometallic compounds were analyzed by X-ray structural analysis, NMR, UV–vis, luminescence, EPR instrumentation, and DFT computational chemistry to investigate their structure and properties. For example, Gabbai and co-workers reported that the reaction of platinum complex **71** with (dichloroiodo)benzene **44** gave chlorinated complex **72** (Scheme 28).^310^ In the presence of dimethylsulfide, complex **72** undergoes a clean photolysis that affords products **71** and **73** (Scheme 29), which is a unique example of a germanium-centered light-induced reduction resulting in the ipso-chlorination of a phenylgermanium species.

**Scheme 30:**
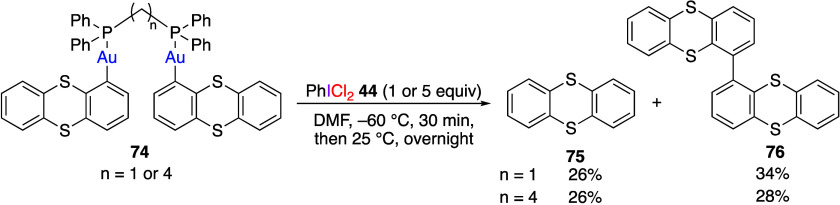
Reaction of Gold Complexes **74** with Reagent **44**

Osakada and Tsuchida et al. have reported that the reaction of gold complexes **74** with (dichloroiodo)benzene **44** yields a mixture of thianthrene **75** and its dimer **76** in moderate yields (Scheme 30).^321^ The authors propose that the reaction pathway leading to these two products are derived from two independent mechanisms. One mechanism is the oxidation of the thiantrenyl ligand to form a radical cation intermediate, while the other oxidation reaction is thought to occur at the two Au centers.^334^

### 3.2 Iodine(III) Compounds with Oxygen Ligands

#### 3.2.1 [Bis(acyloxy)iodo]arenes

[Bis(acyloxy)iodo]arenes represent an important class of hypervalent iodine compounds with many practical applications summarized in numerous previous review articles.^10,147,335−337^ According to CAS SciFinder, over 2,500 research papers on various synthetic uses of just (diacetoxyiodo)benzene, PhI(OAc)_2_, were published between 2016 and 2024; however, most of these applications deal with routine oxidative transformations of various organic substrates, which were summarized in earlier reviews. This section mainly covers novel applications of [bis(acyloxy)iodo]arenes in synthetically useful reactions.

**Scheme 31:**
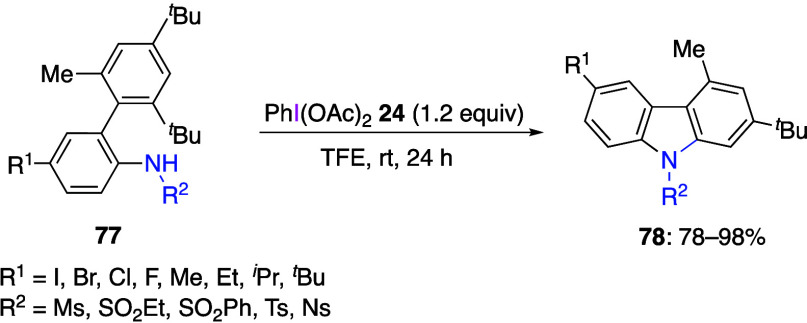
Preparation of Carbazole Compounds **78** from Anilines **77**

Several general synthetic approaches to [bis(acyloxy)iodo]arenes have been developed. The classical general approach to (diacetoxyiodo)arenes is based on the reaction of an oxidant with an iodoarene in acetic acid solution. Older procedures involve the use of potentially explosive concentrated hydrogen peroxide as the oxidant, while recently developed methods utilize the less explosive oxidants.^338−341^ Structures of several recently synthesized (diacetoxyiodo)arenes have been characterized by X-ray analysis.^339,342,343^ In addition to structural characterization, a computational study of the oxidizing capacity of various (diacetoxyiodo)arenes has also been reported.^206^ Synthesis of several other [bis(acyloxy)iodo]arenes by ligand exchange reactions of (diacetoxyiodo)arenes with different carboxylic acids and study of their solubility in organic solvents has been recently published.^344^

(Diacetoxyiodo)arenes are efficient oxidants for a variety of organic substrates. A useful feature of these compounds is that they can be easily converted to different hypervalent iodine reagents during the reaction in the presence of appropriate additives. (Diacetoxyiodo)benzene (DIB) **24** is an efficient reagent that can oxidize phenolic compounds to quinone derivatives resulting from subsequent intra-^345−355^ and intermolecular cyclization or addition reactions.^356−367^ Such dearomatization reactions can produce quinone products from phenols or anilines.^368−377^ The cyclization reactions on nitrogen have been reported for dearomatization of aniline compounds in the absence of external nucleophiles. For example, Mal and co-workers reported that the reaction of sulfonamide derivatives **77** with DIB **24** results in the replacement of a *tert*-butyl group with a new carbon–nitrogen bond to give carbazole products **78** (Scheme 31).^378^ This reaction proceeds successfully for substrates with different substituents on the aromatic ring and various sulfonamide groups. The same group has also reported a cyclization reaction on the sulfur atom of thiophenol instead of the nitrogen atom using DIB **24** as the oxidant.^379^ Other reactions of anilines using DIB have been reported, such as the synthesis of azo compounds by aniline coupling.^380,381^

**Scheme 32:**
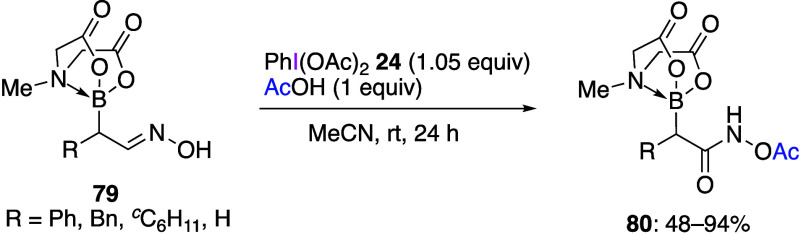
Preparation of *N*-Acetoxyamides **80** from Aldoximes **79**

Oxidation of aldoximes with DIB **24** generates the corresponding nitrile oxides, which react with alkenes or alkynes to give the corresponding heterocyclic compounds.^382−385^ The in situ generated nitrile oxides can also be trapped by the reaction with acetic acid, leading to the formation of *N*-acetylamides.^386^ For example, the reaction of α-MIDA borylaldoximes **79** with DIB **24** in the presence of acetic acid yields the corresponding *N*-acetoxyamides **80** in good yields (Scheme 32). The resulting *N*-acetoxyamides **80** can be further converted to boron-containing hydroxylamines by appropriate treatment. Oxidation of ketoximes with DIB **24** has also been reported.^387,388^

**Scheme 33:**
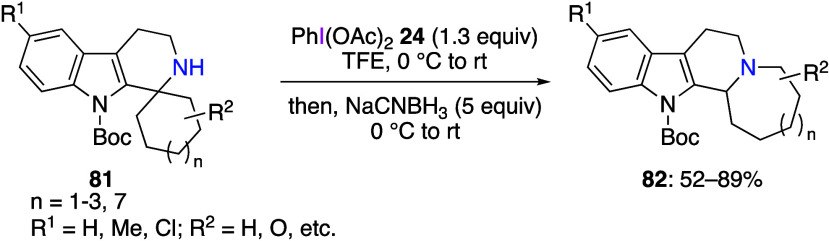
Oxidative Rearrangement of Secondary Amines **81**

**Scheme 34:**
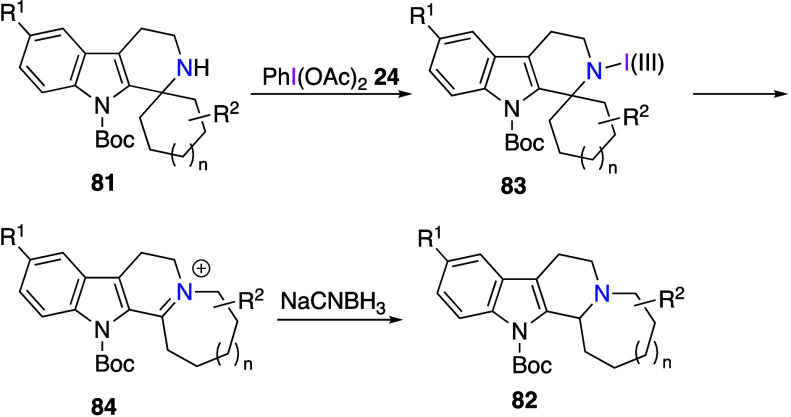
Proposed Mechanism for the Formation of **82**

Various reactions of amines with DIB **24** resulting in the oxidation of nitrogen atom have been reported.^389−408^ For example, Murai and co-workers reported an oxidative rearrangement reaction of amines promoted by DIB.^392^ In this reaction, secondary amines **81** react with DIB in trifluoroethanol solution and are subsequently treated with a reducing agent to obtain rearrangement products **82** in moderate to high yields (Scheme 33). The proposed reaction mechanism includes the in situ generation of N–I(III) intermediate **83** which further undergoes 1,2-carbon-to-nitrogen migration, leading to iminium species **84**. These species are reduced by NaCNBH_3_ to afford final products **82** (Scheme 34). The same group has also reported the oxidative rearrangement reactions using primary amines.^394^

**Scheme 35:**
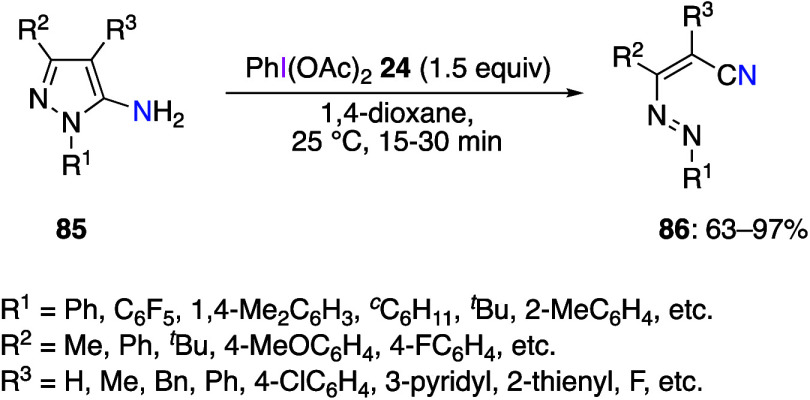
DIB-Mediated Oxidative Cleavage Reaction of 5-Aminopyrazoles **85**

**Scheme 36:**
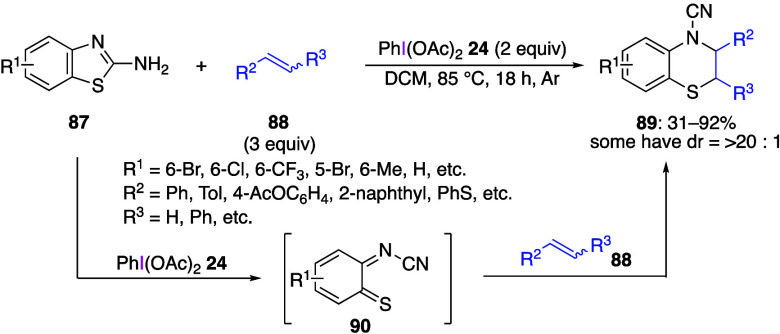
DIB-Mediated Oxidative Ring-Expansion Reaction of 2-Aminobenzothiazoles **87**

Preparation of nitrile compounds by oxidative cleavage of amines by DIB has been reported. The reaction of 5-aminopyrazoles **85** with DIB **24** gives the corresponding 1,2-diaza-1,3-dienes **86** (Scheme 35).^401,405^ The authors believe that radical intermediates are not involved in this reaction because in the presence of radical scavengers such as TEMPO and BHT the reaction affords products **86** in unchanged yields. Bao and Sun reported a similar oxidative cleavage of the amine moiety of 2-aminobenzothiazole upon treatment with DIB, leading to a conjugated nitrile intermediate which could be trapped by a [4 + 2] cycloaddition reaction with alkenes.^408^ The reaction of 2-aminobenzothiazoles **87** with DIB **24** in the presence of alkenes **88** yields dihydro-1,4-benzothiazines **89** (Scheme 36). The key intermediate in this reaction appears to be the conjugated cyano compound **90** formed by the oxidative cleavage of 2-aminobenzothiazole with DIB.

**Scheme 37:**
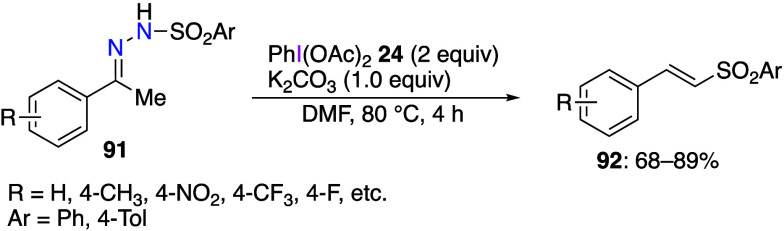
Denitrogenation of *N*-Arylsulfonylhydrazones **91** Using DIB **24**

Luo and He have reported that a denitrogenation reaction occurs when a hydrazone is treated with DIB.^409^ The reaction of *N*-arylsulfonylhydrazones **91** with DIB **24** under basic conditions yields denitrogenated vinylsulfone products **92** (Scheme 37). Since this reaction does not yield products **92** in the presence of TEMPO, the authors suggest that the reaction mechanism involves radicals.

**Scheme 38:**
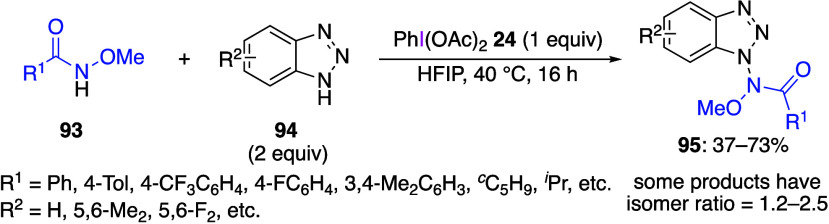
Cross-Coupling Reaction of *N*-Methoxyamide **93** and Benzotriazole **94**

Several Hofmann rearrangement reactions of cyclic imides^410,411^ or amides^412−414^ with hypervalent iodine reagents were previously reported. More recently, the Patureau group reported that the reaction of benzotriazole in the presence of DIB with *N*-methoxyamide as a substrate leads to a nitrogen–nitrogen coupling reaction.^415^ In this reaction, DIB **24** and *N*-methoxy amide **93** are added to the benzotriazole **94** to obtain the nitrogen–nitrogen cross-coupling compound **95** (Scheme 38). Aromatic and aliphatic *N*-methoxyamides **93** can be used in this reaction. In this reaction, if *N*-methoxyamide and DIB were reacted for 16 h and then benzotriazole was added, products **95** were not formed. In contrast, when benzotriazole and DIB were reacted for 16 h and then *N*-methoxyamide **93** was added, the desired product **95** was obtained. The authors suggested that the amide substrates decompose in the presence of DIB, while benzotriazole is stable under these conditions.

**Scheme 39:**
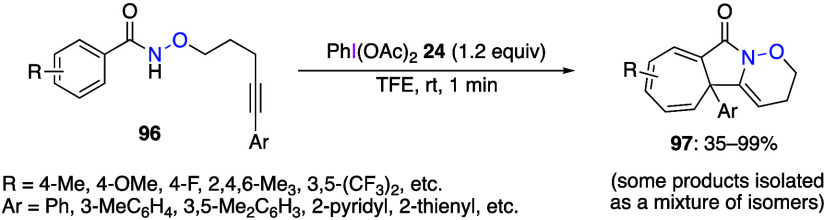
Preparation of Cycloheptatriene-Fused Lactam **97**

Reactions of alkoxy amides with long alkyl chains with alkynes or alkenes instead of methoxy groups have also been reported.^416,417^ When DIB **24** is reacted with alkoxyamides **96** with an alkyne moiety, an intramolecular cyclization reaction proceeds in 1 min to give the corresponding cycloheptatriene-fused lactams **97** (Scheme 39).^417^ A similar cyclization occurs when alkenes are used instead of alkynes. The authors have used DFT calculations to explain the reaction mechanism.

**Scheme 40:**
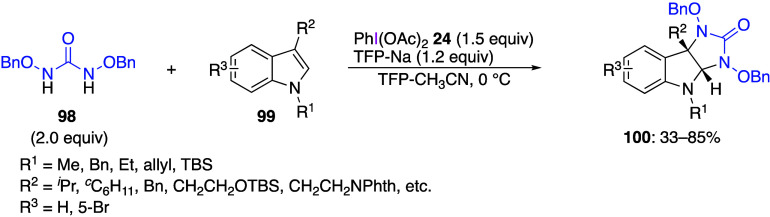
Cycloaddition Reaction of Indoles **99**

**Scheme 41:**
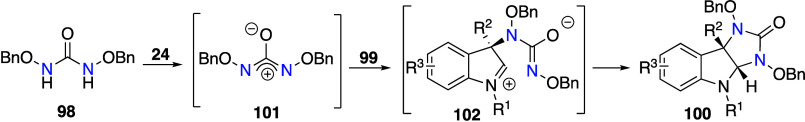
Proposed Mechanism for Cycloaddition Reaction of Compounds **98** and **99** Using Reagent **24**

DIB has been used as an effective reagent in the oxidation reactions of urea derivatives. Jeffrey and co-workers reported that a [2 + 3] cycloaddition reaction proceeds when DIB **24** is added to a mixture of *N*,*N*-dibenzyloxyurea **98** and an indole **99**, yielding the corresponding imidazoloindole products **100** (Scheme 40).^418^ The reaction mechanism includes initial formation of a diazaoxyallylic cationic species **101** by the oxidation of *N*,*N*-dibenzyloxyurea **98** with DIB **24**, which then reacts with indole **99** to produce a zwitterionic intermediate **102**, followed by an intramolecular cyclization reaction to form final product **100** (Scheme 41). Romo and co-workers have reported a similar [2 + 3] cycloaddition reaction with indole of a compound with a guanidine skeleton instead of urea.^419^

**Scheme 42:**
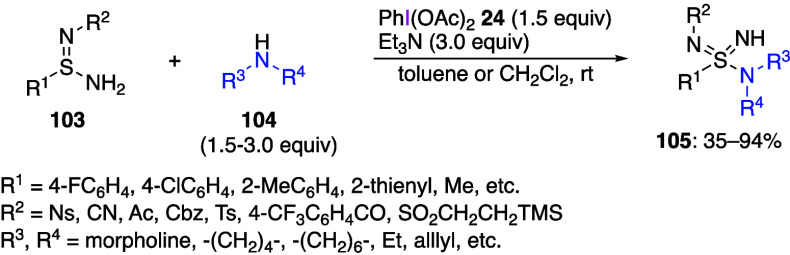
Preparation of Sulfonimidamides **105**

It is known that DIB is an effective oxidant not only for nitrogen compounds but also for sulfur compounds.^420−423^ The oxidation of organic sulfides to sulfoxides using DIB has been well-documented.^183^ On the other hand, the reactions in which sulfur compounds react with nitrogen compounds in the presence of DIB to form various products with a sulfur–nitrogen bond have also been reported recently.^424−428^ For example, Willis and co-workers reported that the reaction of sulfinamidines **103** with DIB **24** and amines **104** yielded sulfonimidamide products **105** with new S–N bonds (Scheme 42).^425^ The reaction proceeds efficiently with sulfinamidines bearing various functional groups and with a variety of amines. This chemistry is suitable for the preparation of several known sulfondiimidamide-derived medicinal agents. The same group has also reported a similar reaction utilizing sulfinamides instead of sulfinamidines. resulting in the corresponding sulfonimidamide products.^428^

**Scheme 43:**
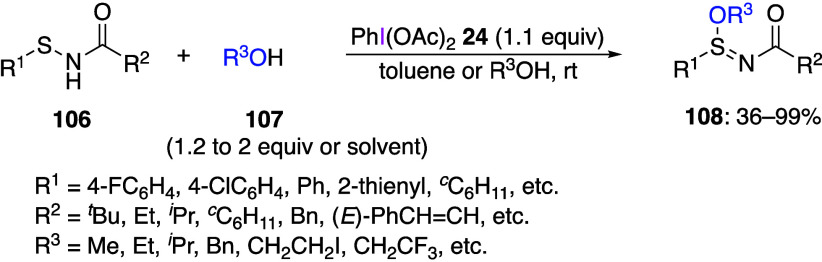
Preparation of Sulfinimidate Esters **108**

Recently, Lu’s group reported the synthesis of sulfinimidate esters from sulfenamides.^429^ In this reaction, the addition of sulfonamide **106** and alcohol **107** to DIB **24** efficiently produced sulfinimidate ester **108** (Scheme 43). A variety of alcohols can be used in this reaction; even when the alcohols derived from natural products (e.g., the hair growth stimulator RU58841 and the estrogen receptor modulator (SERM) ospemifene) are used, the corresponding products **108** are obtained in moderate yields.

**Scheme 44:**
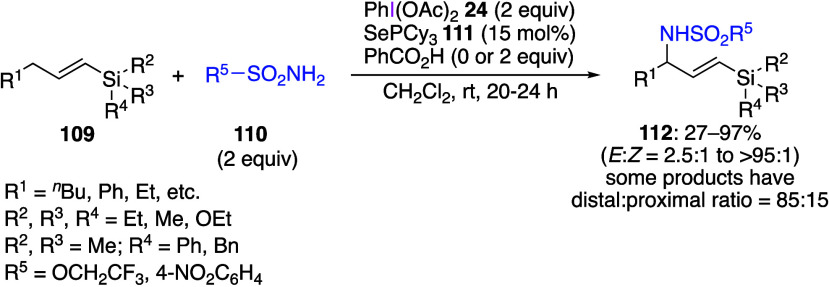
Allylic C–H Amination of **109**

**Scheme 45:**
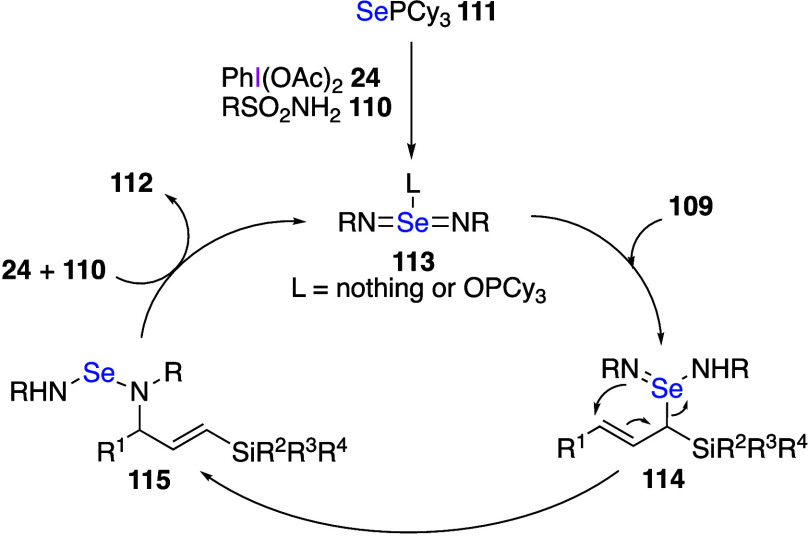
Proposed Mechanism of Product 112 Formation

It was reported recently that the reaction of allyl compounds with DIB, amine, and a catalytic amount of a selenium reagent leads to a metal-free C–H amination of the allylic position.^430−432^ For example, a vinylsilane **109**, a sulfonamide **110**, and a catalytic amount of selenium reagent **111** react with DIB to form compound **112** with the aminated allylic position (Scheme 44).^430^ This reaction also proceeds efficiently when a pinacol boronic acid derivative is used instead of the vinylsilane. The proposed mechanism of this reaction involves initial generation of a reactive selenium bis(imide) species **113**, which undergoes a sequential ene reaction to yield intermediate **114**, followed by a [2,3]-sigmatropic rearrangement affording aminated product **115**. Final product **112** is formed from intermediate **115**, while the selenium catalyst is regenerated from DIB **24** and amine **110** to form selenium bis(imide) species **113** (Scheme 45). The same group has also recently reported that selective C–H amination reactions can also occur for allyl compounds without silyl or boron group substituents.^432^

**Scheme 46:**
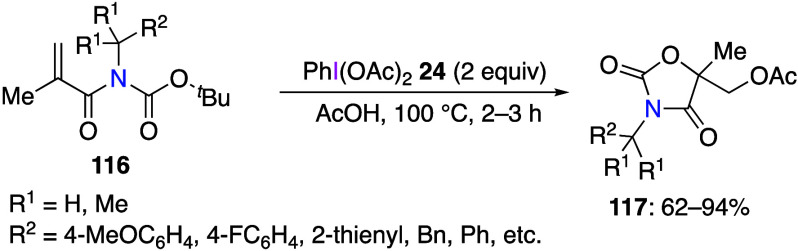
Preparation of 5,5-Disubstituted Oxazolidine-2,4-dione **117**

**Scheme 47:**
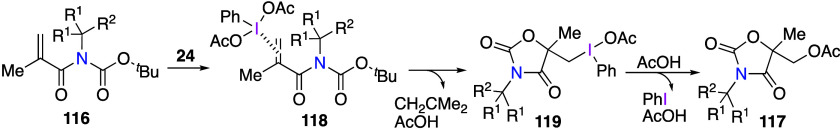
Proposed Mechanism for the Conversion of Compound **116** to Product **117**

DIB and its derivatives are useful reagents for intramolecular cyclization reactions, and numerous cyclization reactions leading to various ring structures have been reported in recent years.^417,433−449^ Cuny and co-workers reported an oxidative cyclization of protected acrylamides **116** using DIB **24** in acetic acid to give oxazolidine-2,4-dione derivatives **117** in good yields (Scheme 46).^442^ The suggested reaction mechansim includes initial coordination of DIB **24** with the double bond of acrylamides **116** via intermediate **118** (Scheme 47) followed by cyclization via elimination of isobutylene to form intermediate **119**. Finally, nucleophilic substitution of the hypervalent iodine leaving group in **119** with an acetate anion affords final product **117**.

**Scheme 48:**
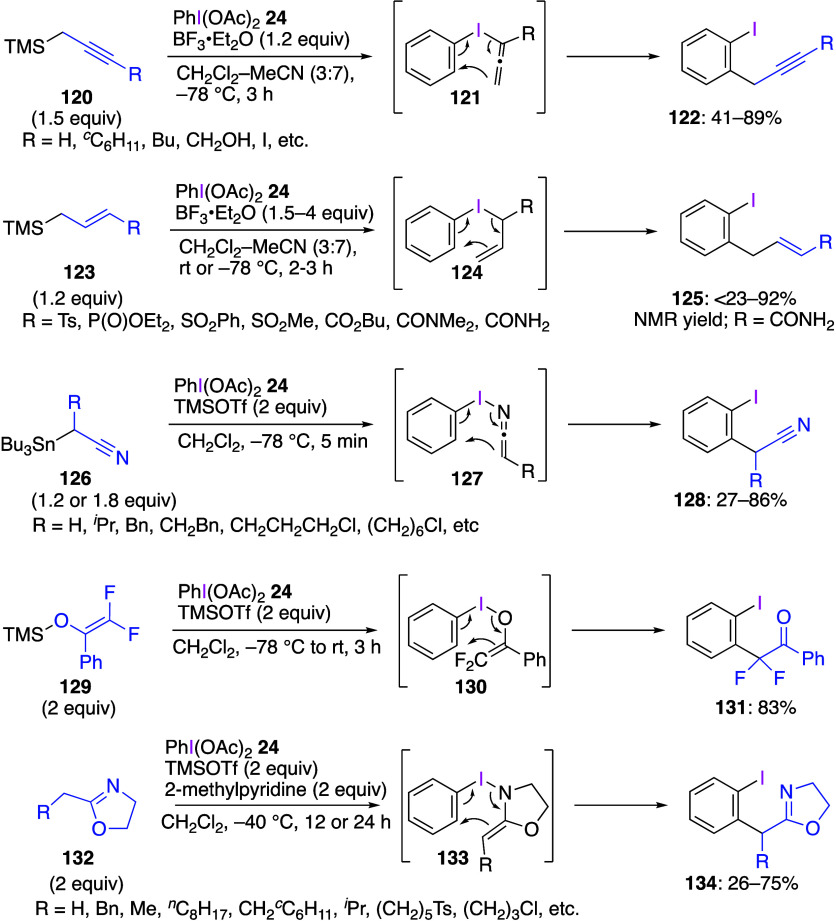
Sigmatropic Rearrangement Reactions Using DIB **24**

Activation of DIB by the addition of Brønsted acid or Lewis acid has been well-documented; however, the structure of the activated species was not reliably confirmed. In 2016, Shafir and co-workers were the first to report the X-ray structure of PhI(OAc)OTf produced by the ligand exchange reaction between DIB and TMSOTf.^450^ This reagent was further converted by dimerization to the Zefirov’s reagent [PhIOIPh](OTf)_2_, the structure of which was also revealed for the first time by X-ray analysis. Furthermore, when BF_3_ was added to DIB instead of TMSOTf, the structure of the activated PhI(OAc)_2_-BF_3_ complex was also confirmed by X-ray analysis, and the reactivity of this complex was experimentally investigated along with NMR studies and DFT calculations. In recent years, a number of sigmatropic rearrangement reactions of unsaturated compounds promoted by DIB-BF_3_ or DIB-TMSOTf have been reported.^450−458^ Several examples of such reactions of DIB with various unsaturated compounds are shown in Scheme 48. In these reactions, DIB **24** reacts with substrates **120**, **123**, **126**, **129**, and **132** to form the corresponding intermediates **121**, **124**, **127**, **130**, and **133**, which then undergo intramolecular sigmatropic reactions to yield final products **122**, **125**, **128**, **131**, and **134**. These reactions can also be carried out using ArI(OAc)_2_ with various substituents in the Ar ring. On the other hand, it has been reported that, under similar reaction conditions, when benzylsilane or benzylborate compounds are used instead of allylsilanes **123**, the insertion reaction proceeds to the *para*-position instead of the *ortho*-position.^459,460^

**Scheme 49:**
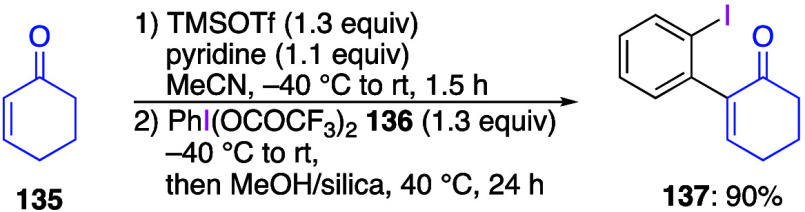
Sigmatropic Rearrangement Reaction Using PIFA **136**

**Scheme 50:**
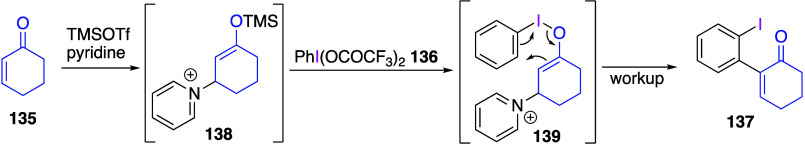
Proposed Mechanism α-Arylation of Ketone **135**

Wengryniuk and co-workers reported the sigmatropic reaction using PIFA and its derivatives instead of DIB.^461^ In this reaction, the addition of pyridine and TMSOTf to 2-cyclohexen-1-one **135** followed by the addition of PIFA **136** leads to the rearrangement product **137** (Scheme 49). The reaction proceeds efficiently even when PIFA with various functional groups is used in this reaction. The proposed mechanism of this reaction involves the formation of β-pyridinium silyl enol ether **138** from pyridine and TMSOTf followed by ligand exchange reaction with PIFA **136** to form an intermediate **139**, which is then converted to final product **137** via a [3,3]-sigmatropic reaction (Scheme 50).

**Scheme 51:**
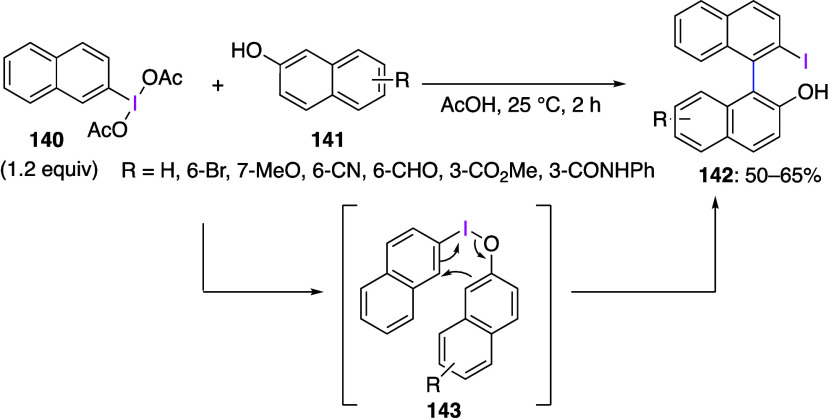
Preparation of Binaphthyl Compounds **142**

Yorimitsu and co-workers reported a sigmatropic reaction using naphthol derivatives. In this reaction, 2-(diacetoxyiodo)naphthalene **140** reacts with 2-naphthols **141** in acetic acid to afford the corresponding binaphthyl derivatives **142** in moderate yield (Scheme 51).^462^ The proposed mechanism of this reaction includes the initial ligand exchange of reagent **140** and 2-naphthol **141** to form intermediate **143**, sigmatropic reaction of which yields product **142**. The authors have also found that products **142** can be converted to π-extended furan compounds upon heating under basic conditions.

**Scheme 52:**
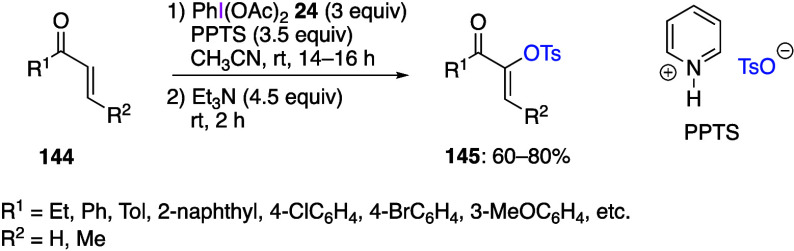
α-Tosyloxylation of α,β-Unsaturated Compounds **144**

**Scheme 53:**
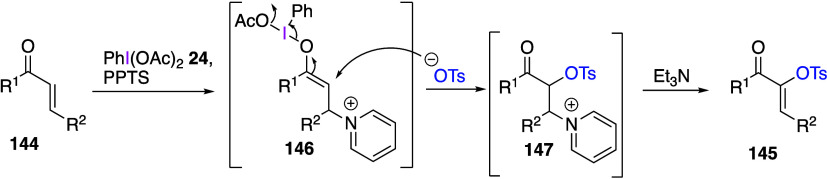
Proposed Mechanism of α-Tosyloxylation Reaction of Compounds **144**

Szpilman’s group reported the umpolung Morita-Baylis-Hillman (MBH) reaction of α,β-unsaturated compounds using DIB.^463^ The reaction of compounds **144** with DIB **24** using pyridinium *p*-toluenesulfonate (PPTS) as a nucleophile, followed by treatment with triethylamine, yields the α-tosylated products **145** in moderate yield (Scheme 52). On the other hand, when acetic acid is used as a nucleophile, an acetoxylation occurs at the α-position, and the initial acetoxylated product is then converted to a 1,2-diketone by passing through a basic column. The proposed mechanism of this reaction includes the initial addition of pyridine and DIB **24** to unsaturated compound **144**, producing an electrophilic enolonium intermediate **146**, followed by the reaction with tosylate anion to form adduct **147**, which is further converted to final product **145** by triethylamine (Scheme 53). The intermediate adduct **146** was detected by NMR. Maulide and co-workers reported the use of DIB and silyl enol ether as the starting material to generate enolonium intermediates, followed by the introduction of an Ar group at the α-position via 1,2-rearrangement of the Ar group.^464^

**Scheme 54:**
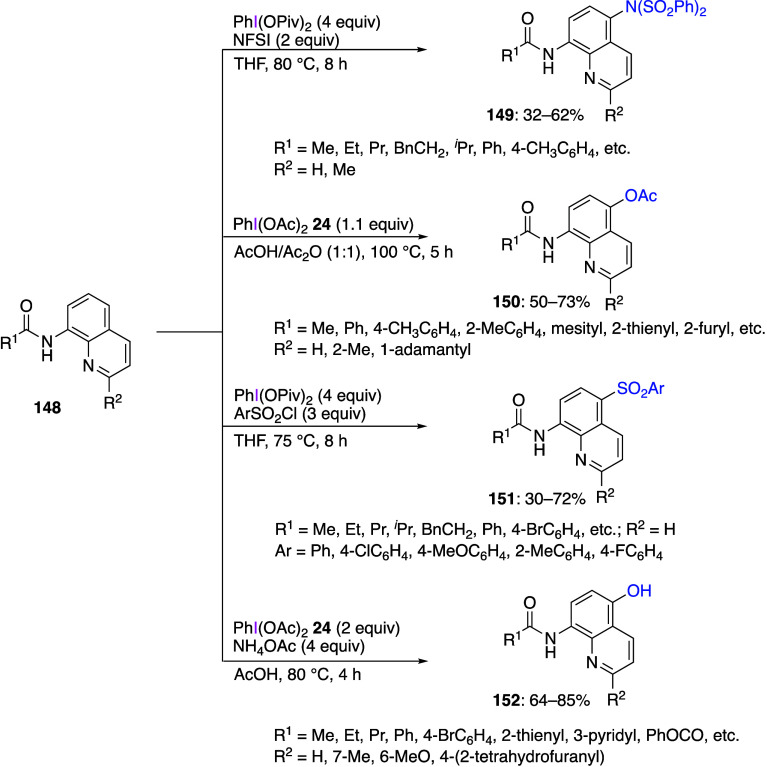
Functionalization of 8-Aminoquinolinamides **148** Using (Diacyloxyiodo)benzene Reagents

Various reactions resulting in a regioselective introduction of a substituent at the C5 position of 8-aminoquinolinamides **148** using DIB or other (diacyloxyiodo)iodobenzene reagents have been reported.^465−469^ Typical examples of such reactions leading to the corresponding products of insertion **149**–**152** in moderate yields are shown below (Scheme 54). It was suggested that the mechanism of these reactions involves initial SET interaction of an iodine(III) reagent with 8-aminoquinolinamides **148**. Similar insertion reactions using metal reagents have also been reported.^470,471^ Other heterocyclic substrates such as imidazopyridines^472−482^ and quinoxalinones^483,484^ have been used for the analogous regioselective insertion reactions at the C3 position.

**Scheme 55:**
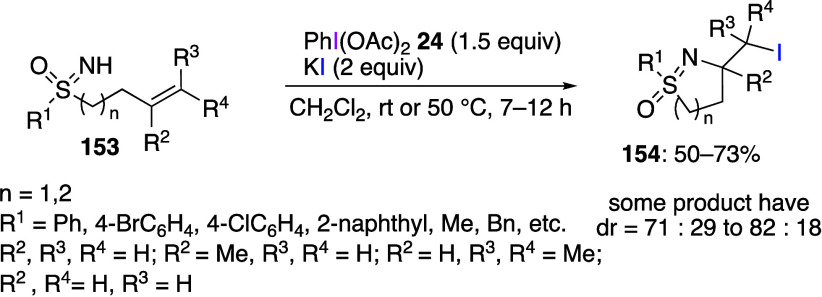
Halocyclization of Sulfoximimes **153** Using DIB/KI Combination

A common methodology involves combining DIB with various additives to generate new active species, which then react with the substrate.^485−508^ In particular, it has been reported that the addition of halide anion sources to PhI(OAc)_2_ results in the formation of hypervalent iodine halide species PhI(OAc)X or PhIX_2_ which are further used for halogenation of various substrates in situ. These species can be used for halogenations of substrates with *sp*^*2*^ carbon,^485,488,489,491,494,507,508^*sp* carbon,^499,500,506^ and *sp*^*3*^ carbon atoms.^492^ It has been reported that halogenation-cyclization reactions of alkenes can be used for the synthesis of various cyclic products.^486,493,497,498,501^ For example, Bolm and co-workers have developed a method for halocyclizations of S-alkenylsulfoximines. Treatment of unsaturated NH-sulfoximines **153** with a combination of DIB **24** and potassium iodide leads to the formation of *S*-oxides of dihydro isothiazoles **154** (*n* = 1) or tetrahydro-1,2-thiazines **154** (*n* = 1) in good yields with high diastereoselectivities and regioselectivities (Scheme 55).

**Scheme 56:**
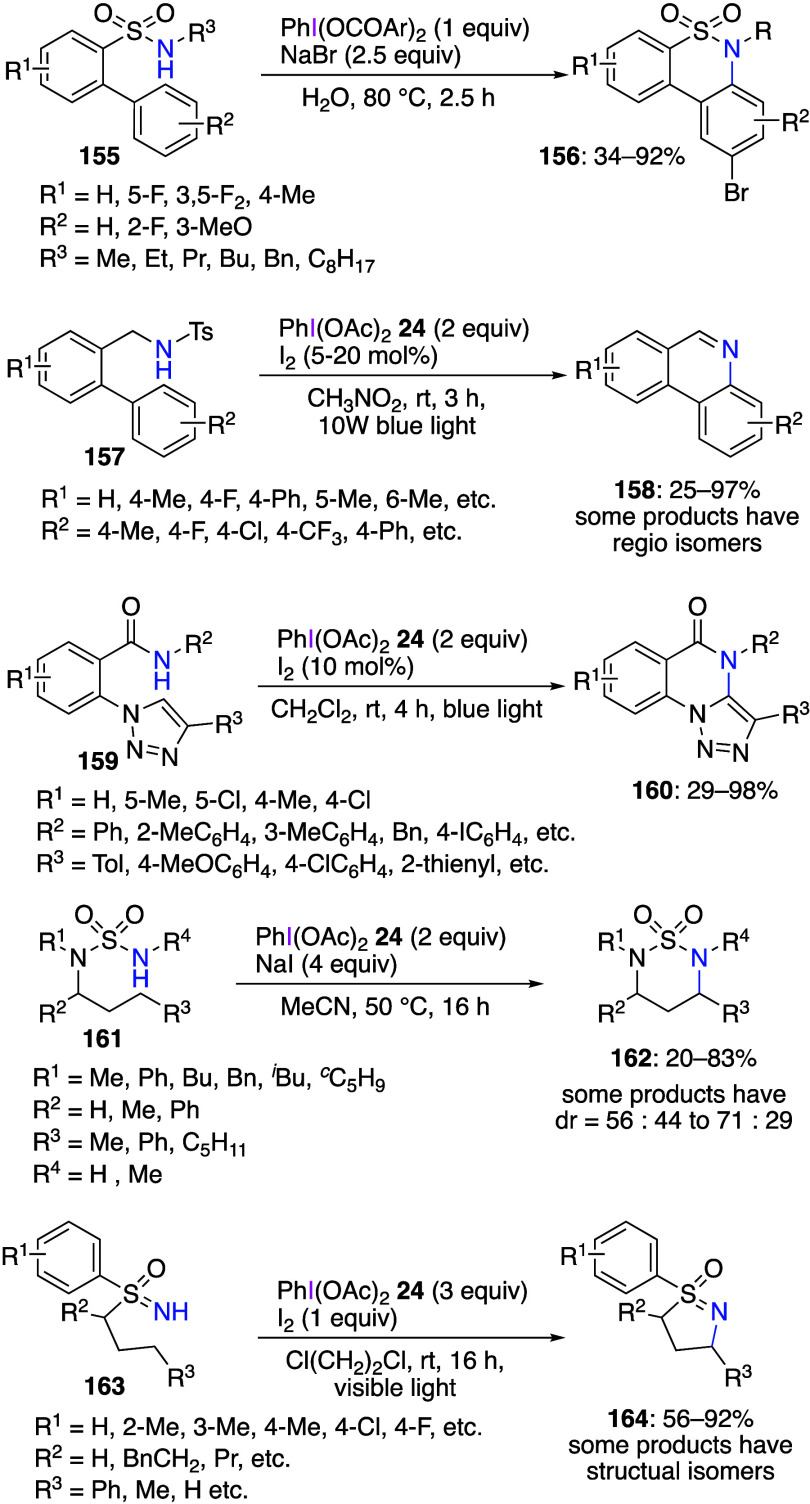
Cyclizations of Amino Derivatives Using DIB **24** with an Additive

This methodology has also been used for halogenation at the nitrogen atom. The generated nitrogen-halogen bond is relatively unstable and quickly breaks by a radical pathway, resulting in inter-^509,510^ or intramolecular amination reactions.^511−520^ Some representative examples of intramolecular amination reactions are shown below (Scheme 56). These amination reactions can proceed at the *sp*^*2*^ or the *sp*^*3*^ carbon atom. It has also been reported that this reaction can be carried out efficiently under photochemical conditions such as visible light and LEDs. DIB and halogen additives can react with various amides, e.g., **155**, **157**, **159**, **161**, and **163**, to obtain the corresponding cyclization products **156**, **158**, **160**, **162**, and **164** in low to high yields. Proposed mechanism of these reactions generally includes homolytic cleavage of the initially formed halogen-nitrogen bond to form a nitrogen radical, which then undergoes an intramolecular HAT followed by the cyclization reaction.

**Scheme 57:**
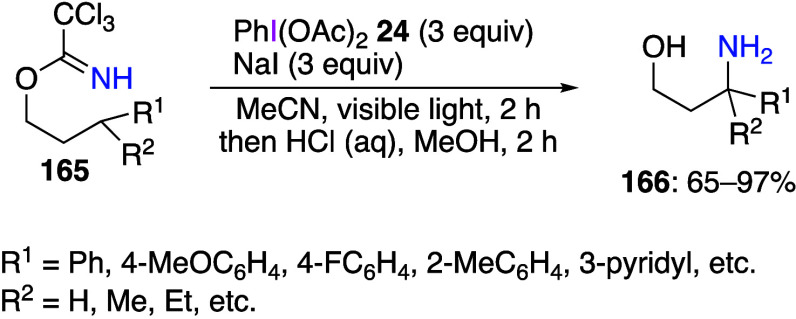
β C–H Amination of Imidate **165**

**Scheme 58:**
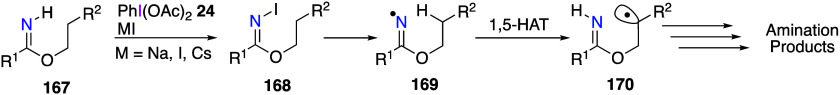
Generation of Carbon Radicals **170** via HAT Reaction

The Nagib group reported various amination reactions utilizing imidate derivatives prepared from nitriles and alcohols.^521−525^ In these reactions, DIB and NaI are added to imidates to form oxazoline products in the mixture, which are then treated with HCl to obtain the desired β C–H amination products (Scheme 57).^521^ The authors found that imidates derived from various alcohols can be used as substrates in this reaction. The same group has also reported that a similar reaction proceeds using a catalytic amount of I_2_ instead of a stoichiometric amount of NaI.^523^ They have also found that the reactions using stoichiometric amounts of CsI yield oxazole products in moderate to high yields.^524^ In these reactions, an N–I intermediate **168** is formed from imidate **167**, DIB **24**, and an iodine additive, followed by homolytic cleavage of the N–I bond to give nitrogen radical species **169** which then undergoes an intramolecular 1,5-HAT reaction, leading to the carbon radical species **170** and the final amination products (Scheme 58). In addition, the authors have also performed a Hammett plot study using 2-arylethanol imidates for the HAT reaction, and the negative slope of the Hammett equation indicated that the reaction proceeded faster for the electron-donating group than for the electron-withdrawing group.^523^ This result suggests that the electron-donating group at the *para*-position stabilizes this transition state, while the electron-withdrawing group has the opposite effect.

In 1998–2005, Kirschning and co-workers reported the preparation and synthetic application of several polymer-supported halogenate(I) complexes by the DIB-promoted oxidation of polystyrene-bound halides.^223,224^ Recently, these reagents were used for the generation of polymer-supported azides which were utilized as reagents in photochemical azidation reactions.^526−528^

**Scheme 59:**
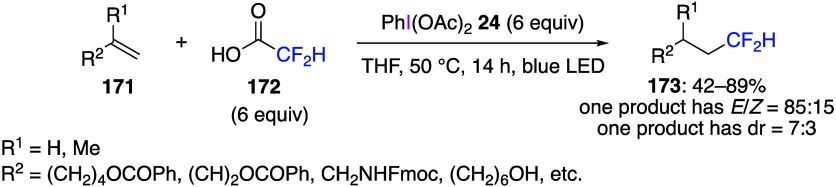
Hydrodifluoromethylation of Alkenes **171**

A reaction utilizing a carboxylic acid as the additive has also been reported. In this reaction, the ligand exchange between DIB and carboxylic acid produces iodine(III) species with a new acyloxy group ligand, which reacts with the substrate as a reagent to perform the desired reaction.^529−533^ The objective of this exchange reaction is not to introduce a new acyloxy group to the substrate, but to add a specific alkyl radical to the reaction substrate, which is generated by decarboxylation of the corresponding acyloxy group under reaction conditions. For example, when DIB **24**, alkenes **171**, and difluoroacetic acid **172** reacted under blue LED irradiation, hydrodifluoromethylated products **173** were obtained (Scheme 59). In this reaction, the iodine(III) reagent produced from DIB and difluoroacetic acid is decarboxylated during the reaction under blue LED irradiation to form difluoromethyl radicals, which further react with alkenes to give the desired product.^529^ The reaction solvent, THF, is used as a hydrogen atom source for the target product (Scheme 59). An example of a similar reaction producing alkyl radicals using sodium alkylsulfinate as an additive has been reported.^534−537^

The other examples of reagent combinations DIB/additive leading to various reactive species in the reaction mixture include the following: DIB/NaN_3_,^538,539^ DIB/K(Na)SCN,^540−542^ and DIB/CF_3_(HCF_2_)SO_2_Na.^534,535^

**Scheme 60:**
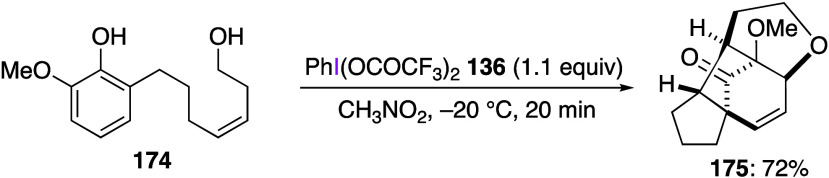
PIFA-Mediated Cascade Reaction of Phenolic Compound **174**

Phenyliodine bis(trifluoroacetate) (PIFA) is a reagent with a generally similar DIB reactivity. Like DIB, this reagent is used as an efficient oxidant for various organic substrates. For example, the oxidation of phenols using PIFA has been reported in numerous publications.^346,363,543−546^ Treatment of a phenolic compound **174** with PIFA **136** affords bicyclo[3.2.1]octane derivative **175** in 72% yield (Scheme 60).^543^ This reaction involves an intramolecular [5 + 2] cyclization of the initial product of dearomatization of phenol **174** with PIFA **136**, leading to final product **175**. The introduction of substituents with electronic and steric effects at the 4- and 5-positions of the substrate yielded the desired products in moderate yields. It has also been reported that the yield of product **175** is slightly lower when DIB is used instead of PIFA in this reaction.

**Scheme 61:**
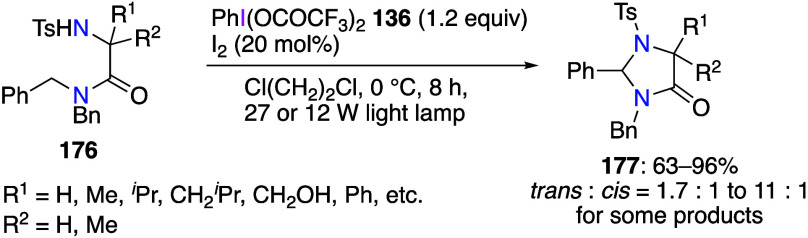
PIFA- and I_2_-Meditated Cyclization of Amines **176**

The use of PIFA for cyclization reactions of various substrates has been reported.^547−556^ Shibata’s group reported that the reaction of amino compounds **176** with PIFA and a catalytic amount of I_2_ under photoirradiation affords cyclization products **177** in moderate to high yields (Scheme 61).^552^ It was demonstrated that PIFA under photoirradiation conditions was the only effective reagent in this cyclization. When DIB was used instead of PIFA, only trace amounts of product **177** were obtained, and that the desired product was not obtained at all without light. The authors suggest that the generated from PIFA and I_2_ trifluoroacetyl hypoiodite IOCOCF_3_ represents the active species in this reaction.

**Scheme 62:**
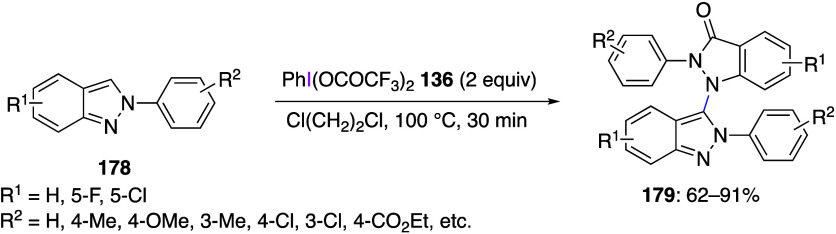
PIFA-Meditated Oxidative Dimerization of 2*H*-Indazole **178**

PIFA can be used as an intermolecular coupling reagent.^557−564^ The dimerization of substrates by oxidative dearomatization with PIFA was reported by Hajra and co-workers.^564^ The reaction of 2*H*-indazole **178** as a substrate with PIFA afforded *N*-1-indazolyl indazolones **179** in moderate to high yields (Scheme 62). The authors suggest that the trifluoromethyl acetate ligand of PIFA serves as the oxygen source in product **179**. They also believe that this reaction proceeds by SET mechanism.

In addition to these reactions, numerous oxidations with PIFA have been reported in the previously published review articles.^565,566^

**Scheme 63:**
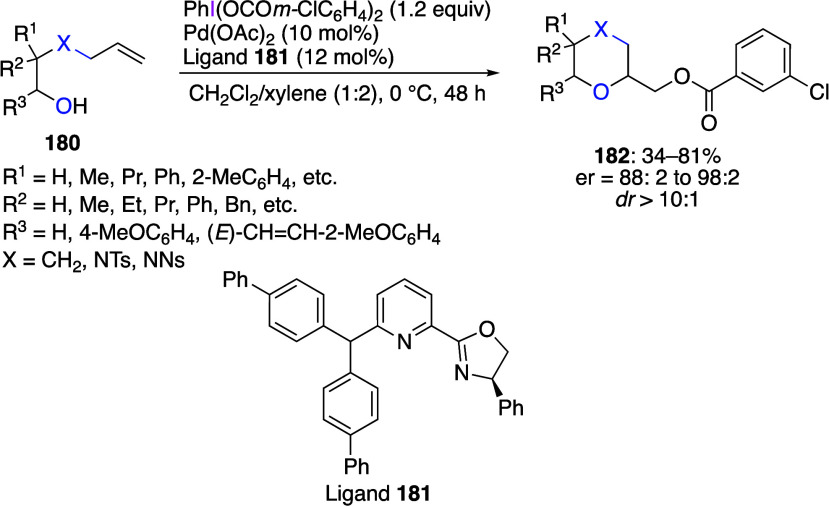
Palladium-Catalyzed Cyclization of Alkenols **180**

DIB, PIFA, and other [bis(acyloxy)iodo]arenes are commonly used as oxidizing reagents in the presence of various transition metal caatalysts.^125,152,567,568^ In the past few years, numerous oxidations catalyzed by compounds of transition metals such as palladium,^569−595^ copper,^470,596−602^ silver,^603,604^ gold,^605−610^ nickel,^611−613^ cobalt,^614,615^ manganese,^616−619^ rhodium,^620,621^ platinum^466,622,623^ vanadium,^624^ rhenium,^625^ ruthenium,^626^ and iron^627−629^ have been reported. Beccalli and co-workers reported palladium-catalyzed enantioselective and regioselective oxidative cycloaddition reactions of alkenol compounds.^588^ In this reaction, the addition of iodine(III) reagent and palladium catalyst to alkenol **180** in the presence of a catalytic amount of pyridinyl oxazoline ligand **181** gives the corresponding six-membered ring product **182** enantioselectively (Scheme 63). The addition of the ligand is important in this reaction. In the absence of ligand, product **182** is obtained in a low yield in a complex mixture of by-products.

**Scheme 64:**
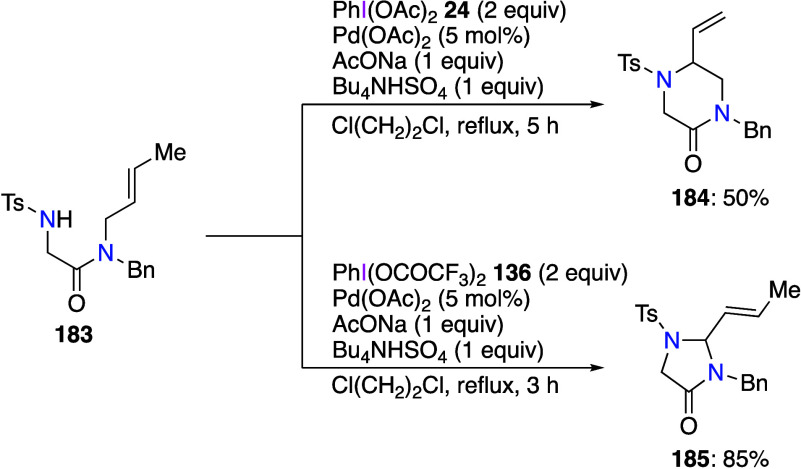
Palladium-Catalyzed Cyclizations of Benzylamide **183**

The palladium(II)-catalyzed reactions of DIB or PIFA with the same substrate but leading to different products were reported by Broggini and Poli.^578^ For example, the reaction of *N*-tosylglycine *N*′-crotyl-*N*′-benzylamide **183** with DIB and catalytic amounts of Pd(OAc)_2_ gave 5-vinylpiperazinone **184** in 50% yield (Scheme 64). The same substrate reacts with PIFA under the same conditions to afford 2-vinylimidazolidinone **185** in 78% yield. The authors have used this difference in reactivity with DIB and PIFA to synthesize several new products.

**Scheme 65:**
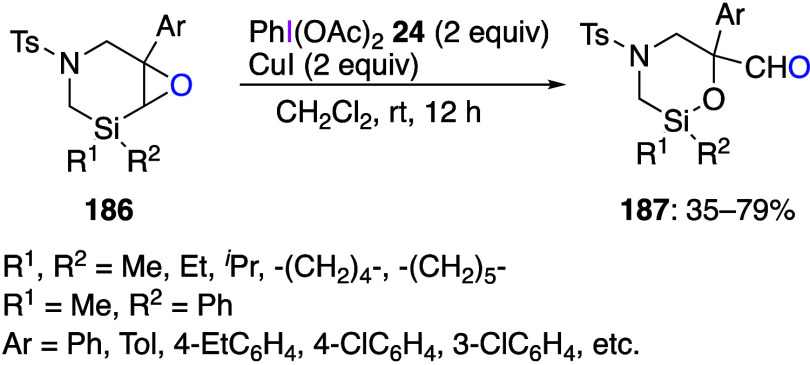
Ring Rearrangement of 1,3-Azasilinyl-4-Epoxides **186**

Gao and Song reported that the reaction of bicyclic epoxides with DIB in the presence of a copper catalyst leads to rearranged products.^601^ The reaction of 1,3-azasilinyl-4-epoxides **186** with DIB and CuI yielded ring-restructured aldehydes **187** (Scheme 65). Additional experiments have demonstrated that this reaction is specific for silicon compounds. According to ^13^C NMR and ESI-MS measurements, this reaction in the presence of ^18^O-water yielded products with Si–^18^O bond, which confirms that the oxygen atom in the aldehyde product was oxygen from epoxide. Based on the control experiments, the authors suggest that the in situ generated Cu(II) intermediates are possible active species in this catalytic reaction.

**Scheme 66:**
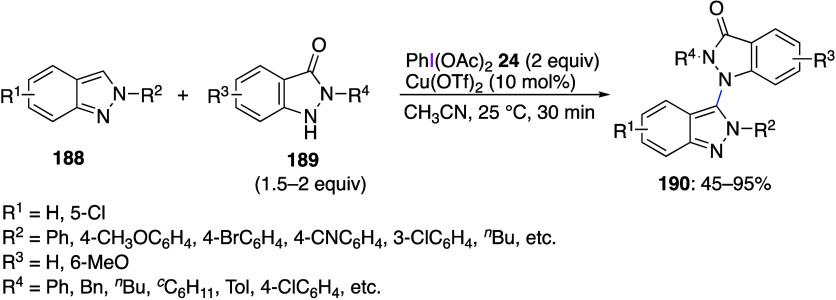
Copper-Catalyzed Coupling Reaction Between **188** and **189**

Copper-catalyzed C–H amination reactions were reported by Cai and Xia.^602^ In the reaction of 2*H*-indazole **188** and indazol-3(2*H*)-one **189** with DIB **24** and a catalytic amount of copper(II) triflate, product **190** aminated at the C3 position of 2*H*-indazole was obtained (Scheme 66). In the presence of a radical scavenger such as BHT, an adduct of indazol-3(2*H*)-one **189** and BHT was isolated, implying that radicals derived from indazol-3(2*H*)-one were involved in the reaction. The role of DIB in this reaction is in the reoxidation of Cu(I) to Cu(II) active species.

**Scheme 67:**
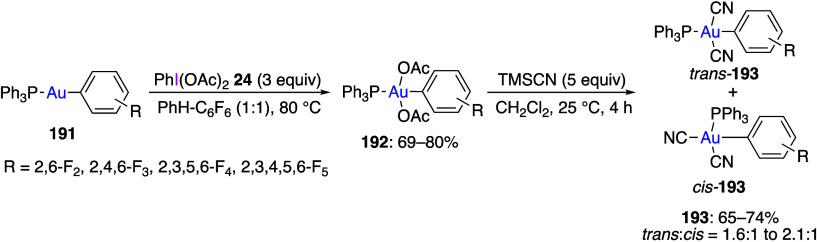
Preparation of Gold(III) Complexes from **191** Using DIB **24**

Nevado’s group reported a successful synthesis of an Au(III) complex from Au(I) compound using the oxidizing power of DIB.^610^ The reaction of DIB with polyfluoroaryl gold(I) compounds **191** gave *trans*-diacetoaryl gold(III) complexes **192** in moderate to high yields (Scheme 67). Reaction of these gold(III) complexes **192** with excess amounts of TMSCN led to ligand exchange reactions, yielding a mixture of *trans*- and *cis*-type gold(III) complexes **193** with cyano ligand. The authors have also performed a reductive elimination reaction of the *trans*-gold(III) complex by heating in 1,4-dioxane solution to afford the corresponding polyfluorinated benzonitriles in high yield.

**Scheme 68:**
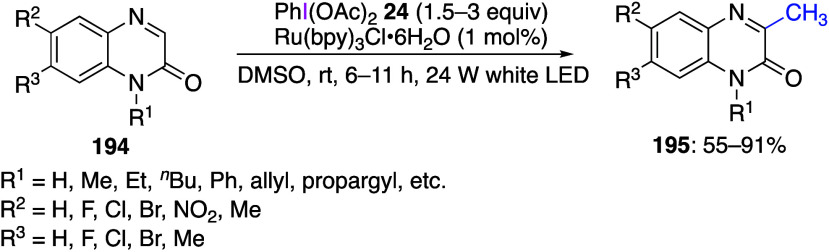
Photocatalyst-Mediated Decarboxylative Methylation of **194**

Photocatalytic reactions of DIB have also been reported.^10,630−635^ The proposed mechanism of these reactions involves the initial interaction of a photocatalyst with light to generate excited chemical species. The excited species are then oxidized by an iodine(III) reagent to form active species. The active species reacts with the substrate and returns to the original catalytic species to initiate the next cycle. For example, it has been reported that DIB **24** and quinoxaline-2(1*H*)-one **194** react under 24 W LED irradiation in the presence of a photocatalyst to yield product **195** with a methyl group introduced at the C3 position of quinoxaline-2(1*H*)-one (Scheme 68).^632^ DIB serves as the source of the methyl group in this reaction, and when a different carboxylic acid is added, a product with an alkyl group of the carboxylic acid introduced instead of a methyl group is obtained. Products **195** were not obtained in the presence of TEMPO, which indicated that radical species were involved in the reaction mechanism.

#### 3.2.2 Iodosylarenes

Iodosylbenzene and other iodosylarenes have been widely used as effective oxidizing reagents for over a century.^221−224^ Iodosylarenes in general have a polymeric structure and are insoluble in common organic solvents.^116,183^ In the presence of a polar protic solvent such as an alcohol, iodosylbenzene dissolves, forming new hypervalent iodine species with an alkoxy group as a ligand. The structure of some of these alkoxy-substituted species was confirmed by X-ray analysis;^636^ however, these compounds are unstable and quickly revert back to iodosylbenzene. Mckenzie and co-workers reported the crystal structure of iodosylbenzene using a combination of various analytical instruments and crystallographic approaches.^637^ Based on the structural analysis, the authors consider that not only the intermolecular interactions of O–I···O–I but also the C–H···π interactions between adjacent phenyl groups contribute to the insolubility of PhIO. Some iodosylbenzene derivatives can dissolve in common organic solvents due to the presence of additional intramolecular interactions.^638^ The soluble iodosyl compounds have a basic oxygen atom and can form complexes with acidic alcohol such as HFIP; the structure of such a complex was characterized by NMR and X-ray crystallography.^639^ Iodosyl compounds with pseudocyclic structure that form the complex with TFA have also been reported.^640^ Recently, it has been reported that iodosyl compounds form complexes with various metals, and their structures have been further investigated by X-ray crystallography.^639,641−647^ A common synthesis of iodosylbenzene involves the treatment of (diacetoxyiodo)arenes with a base. In a recently reported method, iodosylarenes are conveniently prepared by the reaction of iodoarenes with sodium hypochlorite under CO_2_ conditions.^648^

**Scheme 69:**
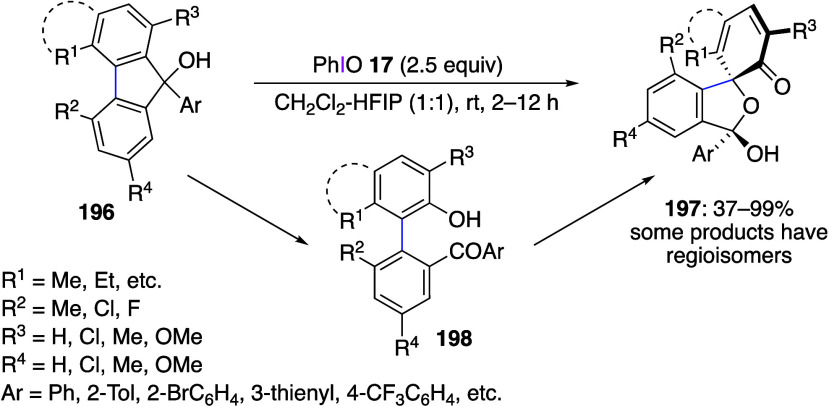
Preparation of Oxo-Spiro Compounds **197** from **196** Using PhIO **17**

Iodosylarenes, similar to (diacetoxyiodo)arenes, can be used as efficient oxidizing reagents. Oxidations of phenols,^649,650^ anilines,^651−653^ alcohols and enolates^654−657^ have been recently reported. For example, Gu and co-workers reported that the reaction of 9*H*-fluorene-9-ols **196** with iodosylbenzene **17** gave the corresponding oxo-spiro compounds **197** (Scheme 69).^656^ The resulting compounds could be converted to biphenyl products by acid treatment. The authors suggested that a phenolic compound **198** is initially formed from the starting material **196** and PhIO **17**, which is further converted to final product **197** by oxidative dearomatization.

**Scheme 70:**
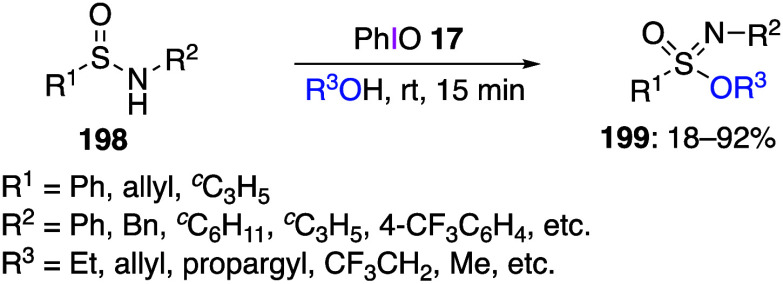
Synthesis of Sulfonimidates **199** from Sulfinamides **198** and PhIO in the Presence of Alcohols

Stockman and co-workers reported synthesis of sulfonimidates from sulfinamides using PhIO as an oxidant.^658^ Sulfinamides **198** react with PhIO in an alcohol solution to form alkoxy-substituted sulfonimidates **199** in moderate to high yields (Scheme 70). Weakly nucleophilic alcohols and unsaturated alcohols can be used in this reaction. The resulting products **199** can be converted to sulfoximines by reaction with Grignard reagents.

**Scheme 71:**
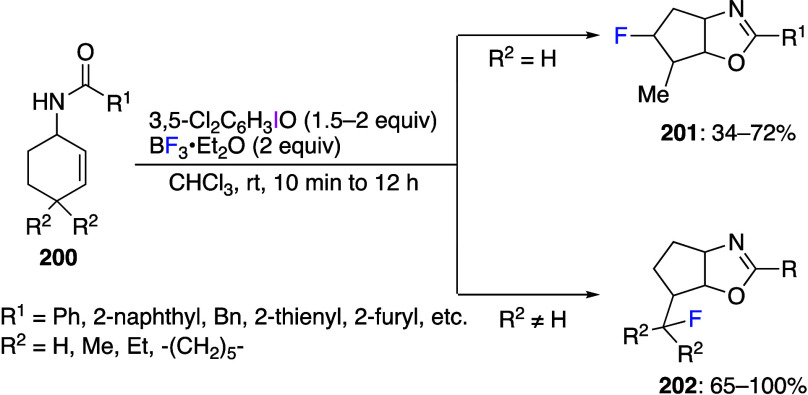
Preparation of Monofluorinated Five-Membered Ring-Fused Oxazolines

Iodosylarenes are commonly utilized as an oxidant in the construction of heterocyclic molecules. It is especially useful for intramolecular cyclizations of various unsaturated precursors.^659−666^ It has been reported that the reaction of *N*-cyclohexenylamide **200** with iodosylarene and BF_3_ yields monofluorinated five-membered fused oxazolines **201** and **202** (Scheme 71).^661^ In this reaction, BF_3_ not only activates the ArIO but also serves as a fluorine source. The position of fluorine introduction depends on the substituents in the substrate. When substituent R^2^ = H at the 4-position of the cyclohexenyl moiety in substrate **200**, fluorine is introduced into the ring of product **201**, and when substituent R^2^ ≠ H, fluorine is introduced outside the ring of the product **202**. In such regioselective fluorination reactions, the stability of the intermediate carbocation is believed to be important. When acetonitrile was used as the reaction solvent, the Ritter-type reaction proceeded and the acetamide group was inserted instead of fluorine, indicating that carbocation was formed during reaction.

**Scheme 72:**
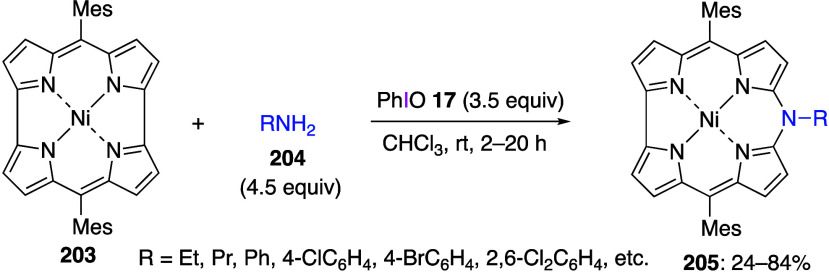
Synthesis of 10-Azacorroles **205**

Iodosyl compounds are useful reagents not only for intramolecular reactions but also for intermolecular reactions. For example, the reaction of 1,3-dicarbonyl compounds in the presence of carboxylic acids leads to α-acyloxylated products.^667^ Recently, Dong and co-workers reported that the reaction of a 1,3-dicarbonyl compound, β-oxoamide, with a cyclic amidine gave a polysubstituted imidazolidin-2-one.^668^ Chmielewski and Li have reported the insertion reaction of amines into conjugated macrocycles using PhIO.^669^ When the nickel-centered macrocycle **203** was treated with amine **204** and an excess amount of PhIO **17**, 10-azacorroles **205** were obtained (Scheme 72). Structures of several products **205** were established by X-ray analysis.

**Scheme 73:**
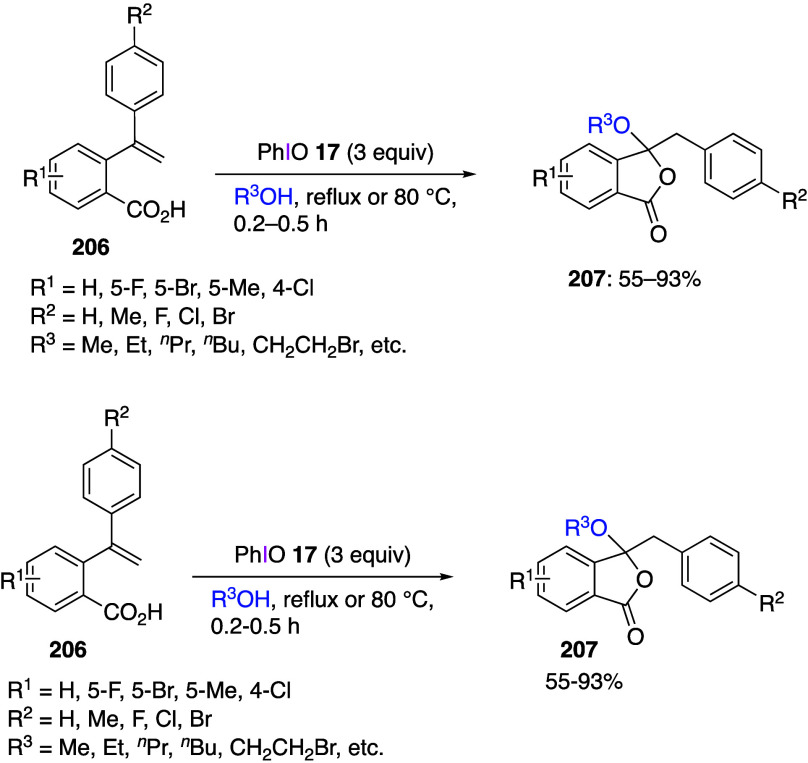
Lactonization Reaction of **206** with PhIO in Alcohol Solutions

Iodosyl compound can be converted to new hypervalent iodine active species in the presence of appropriate additives. For example, in alcohol solutions, alkoxy-substituted hypervalent iodine species are generated.^670,671^ Du and Zhao reported a lactonization reaction promoted by these iodine(III) species.^671^ When 2-(1-phenylvinyl)benzoic acid **206** is reacted with PhIO in alcohol as a solvent under heating, the corresponding lactones **207** are obtained (Scheme 73). In this reaction, the generated dialkoxyiodine(III) species react with the alkene moiety of the starting material, followed by 1,2-migration of the aryl group and alkoxylation, leading to final products. The reactions utilizing iodine(III) species generated in the presence of such additives as diselenide,^672,673^ iodine source,^215,674^ AlX_3_ (X = NO_3_, Cl, Br),^675−678^ carboxylic acid,^679^ and fluoride source^238,244,680−685^ have been reported.

**Scheme 74:**
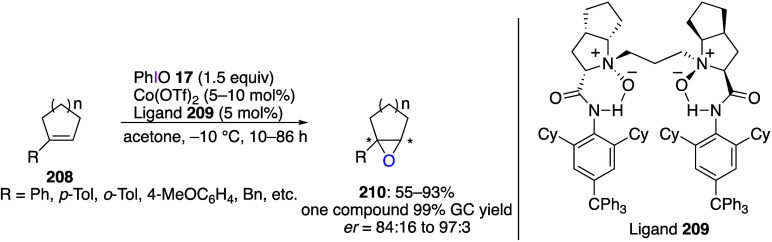
Asymetric Epoxidation of Alkenes **208**

Reactions of PhIO in the presence of metal compounds such as Mn,^686−692^ Cu,^693^ Fe,^694^ Ru,^695^ and Co^696^ as catalysts have been reported. For example, Feng and Liu reported the asymmetric epoxidation of alkenes using Co catalysts with chiral ligand.^696^ When cyclic trisubstituted alkenes **208** react with PhIO **17** and Co catalysts in the presence of chiral ligand **209**, the corresponding epoxy products **210** are obtained with high enantioselectivity (Scheme 74). The reaction also proceeds with acyclic alkenes. The authors have detected the generation of intermediate Co–O species from PhIO and Co catalyst during this reaction by ESI mass spectrometry and have investigated the enantioselectivity by DFT calculations.

### 3.3 Iodine(III) Compounds with Nitrogen Ligands

#### 3.3.1 [(Diamido)iodo]arenes

**Scheme 75:**
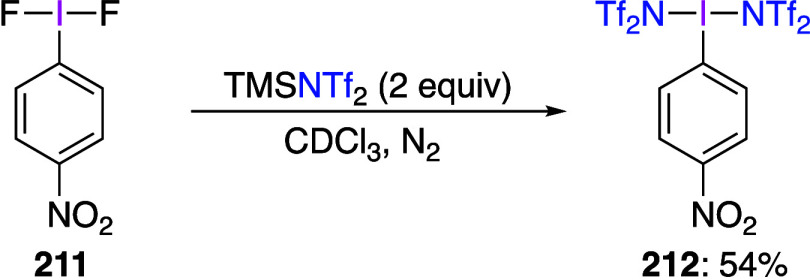
Synthesis of 4-NO_2_C_6_H_4_I(NTf_2_)_2_**212**

[(Diamido)iodo]arenes are efficient reagents for introducing amido group into various organic substrates.^82^ These compounds are generally prepared by a ligand exchange reaction between (diacetoxyiodo)arenes and a trimethylsilyl-substituted amide or imide. Several (diamidoiodo)arenes have been investigated by X-ray structural analysis.^697^ Compounds with bis(trifluoromethanesulfonyl)imide group as a ligand were also synthesized, but their structures were not reliably confirmed in earlier works.^698^ Recently, Dutton and co-workers have reported X-ray structure of *para*-nitro-substituted (diamidoiodo)arene **212**, which was prepared by the reaction (difluoroiodo)arene **211** with TMSNTf_2_ in CDCl_3_ (Scheme 75).^187,212^ The X-ray structure revealed that the I–N bond distance of this compound is longer than other previously reported I–N bonds. The authors have shown that compound **212** has better oxidizing power than 4-NO_2_C_6_H_4_I(OTf)_2_. For example, 3-methylcyclohexene is oxidized to toluene by 4-NO_2_C_6_H_4_I(NTf_2_)_2_ but not by 4-NO_2_C_6_H_4_I(OTf)_2_. The high oxidation potential of 4-NO_2_C_6_H_4_I(NTf_2_)_2_ is also confirmed by calculations using the B3LYP/6-311+G(d,p) method.

**Scheme 76:**
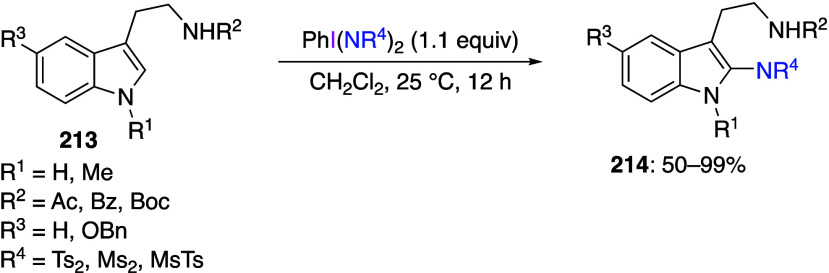
Amination of Indole Compounds **213**

Muñiz and co-workers reported that the reaction of indole compounds **213** with PhI(NTs_2_)_2_ gave products **214** aminated at the 2-position of the indole (Scheme 76).^699^ When PhI(NMs_2_)_2_ or PhI(NTsMs)_2_ is used instead of PhI(NTs_2_)_2_, the corresponding products **214** are also obtained in good yields.

**Scheme 77:**
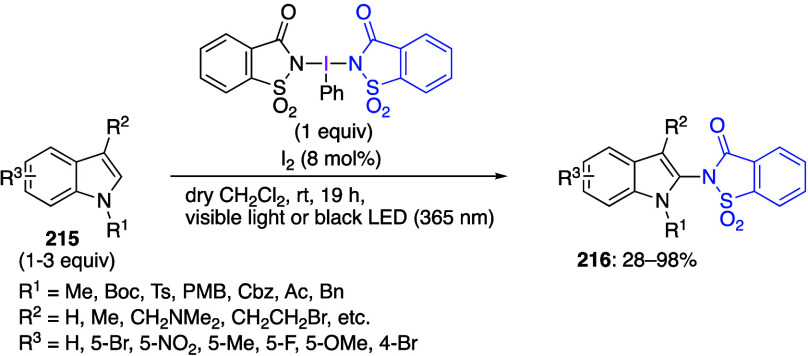
Selective C–H Amidation of Indole **215**

A saccharin analogue of PhI[N(SO_2_R)_2_]_2_ has been prepared and investigated.^697^ It has been demonstrated that this reagent can react with indole derivatives **215** in the presence of a catalytic amount of I_2_ under light irradiation to produce compound **216** with saccharin moiety introduced at the 2-position of the indole (Scheme 77). This iodine(III) reagent serves as an oxidant and a nitrogen source in this reaction. Under similar reaction conditions, it can be used for regioselective introduction of saccharin moiety into various other heterocyclic compounds.

#### 3.3.2 [Acyloxy(amido)iodo]arenes

**Scheme 78:**
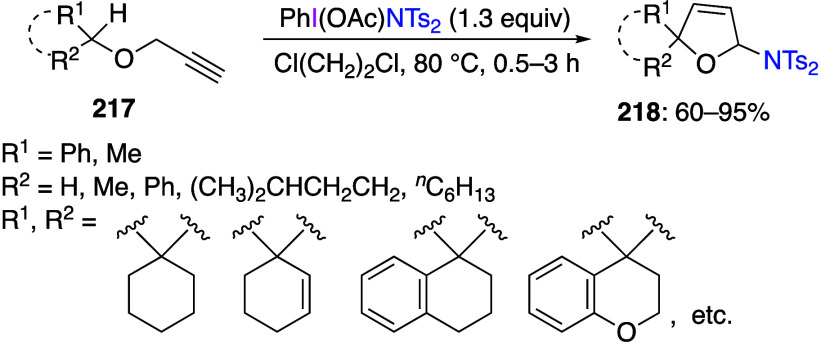
Preparation of NTs_2_-Substituted 2,5-Dihydrofuran **218**

**Scheme 79:**
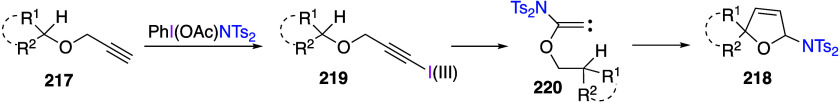
Proposed Reaction Mechanism for the Synthesis of **218** from **217**

[Acyloxy(amido)iodo]arenes can be generally synthesized by adding an equal amount of a trimethylsilyl-substituted amide or imide to (diacetoxyiodo)arene. The structure of these compounds was confirmed by X-ray crystallography.^82,700^ [Acyloxy(amido)iodo]arene can be further converted to [(diamido)iodo]arene by adding a second equivalent of trimethylsilyl-substituted amide. [Acyloxy(amido)iodo]arenes are useful as the amidation reagents similar to [(diamido)iodo]arenes. For example, it has been reported that the reaction of alkoxyalkynes **217** with PhI(OAc)NTs_2_ under heating efficiently affords NTs_2_-substituted 2,5-dihydrofuran **218** (Scheme 78).^701^ The proposed reaction mechanism involves the reaction of PhI(OAc)NTs_2_ with alkyne **217** to form intermediate alkynyliodine(III) species **219**. Subsequent Michael addition of the NTs_2_ anion generates alkylidene carbene **220**, which is further converted to final product **218** by intramolecular 1,5-C–H insertion (Scheme 79).

**Scheme 80:**
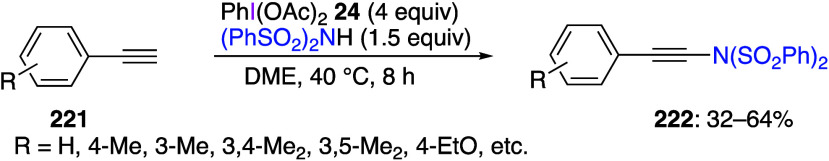
Preparation of (PhSO_2_)_2_N-Substituted Alkynes **222** from Alkynes **221**

PhI(OAc)N(SO_2_R)_2_ reagents can be be generated and utilized in situ.^702^ For example, the reaction of aromatic alkynes **221** with DIB **24** and dibenzenesulfonimides provides the corresponding ynamides **222** in moderate yields (Scheme 80).^703^ In this reaction, alkylidene carbene is also generated as a key intermediate, but because aryl-substituted alkynes are used, the common 1,2-rearrangement occurs, leading to alkynes **222**.

**Scheme 81:**
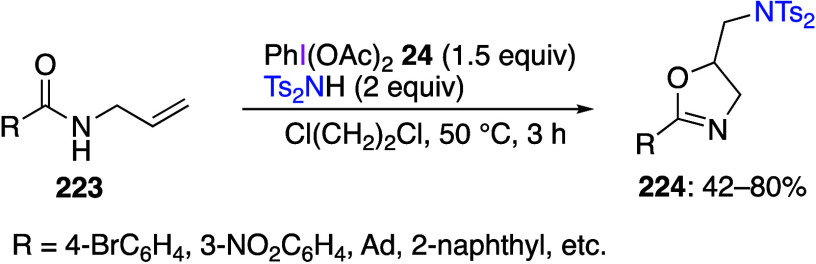
Oxidative Cyclization of *N*-Allylamides **223**

An example of the formation and use of the PhI(OAc)NTs_2_ reagent in situ has been reported for the cyclization of *N*-allylamides.^704^ The reaction of DIB **24** and bis-tosylimide with *N*-allylamides **223** gives the corresponding oxazolines **224** (Scheme 81). Under similar conditions, the reaction of *N*-allylthioamides afforded thiazolines. In these reactions, the generated PhI(OAc)NTs_2_ species activate the alkene moiety, initializing the cyclization.

#### 3.3.3 Iodonium Imides

**Scheme 82:**
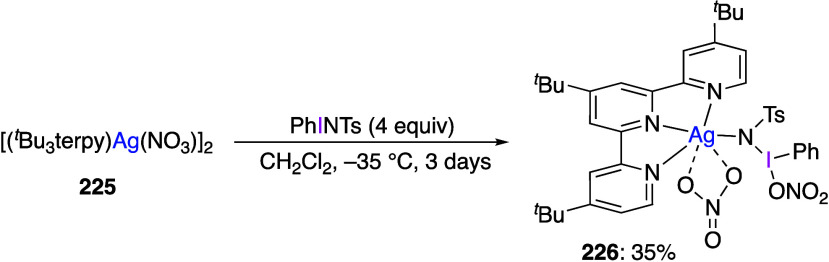
Preparation of Ag(II) Complex **226**

Iodonium sulfonimides, PhI = NSO_2_R, can be generally synthesized from DIB and sulfonamides under basic conditions and are known to be insoluble in common organic solvents due to the polymeric structure.^85^ It has been demonstrated that the introduction of a coordinating substituent in the *ortho*-position of the phenyl ring improves the solubility and stability of iodonium imides and improves their reactivity. The most common synthetic application of these reagents is the transfer of the imino group to the substrate, but it is also known that they can be used as oxidants.^705^ Typical imino group transfer reactions are represented by the oxidative iminination of various transition metal compounds, such as complexes of Cu,^706^ Fe,^707−709^ and Ag.^710^ For example, Campbell and co-workers reported that the addition of PhINTs to a Ag(I) complex **225** gave an Ag(II) complex **226** (Scheme 82), whose structure was determined by X-ray crystallography.^710^ In a similar reaction of Ag(I) complex **225** with an excess amount of PhINSO_2_Ph, the corresponding Ag(II) complexes containing N(SO_2_Ph)_2_ were obtained in high yields and confirmed by X-ray analysis. Furthermore, the authors have confirmed by NMR that a Ag(III) complex can be generated in the system from an excess amount of iminiodane with Ag(I) complex, and they suggested that this is the active species in the silver-catalyzed aziridination of alkenes by PhINTs.

**Scheme 83:**
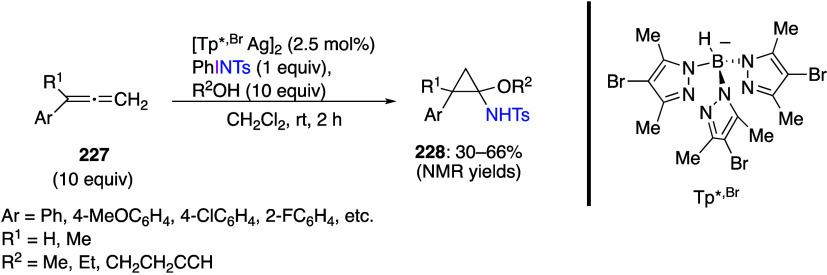
Synthesis of Aminocyclopropanes **228** from Allenes **227**

Typical metal-catalyzed reactions of iminoiodanes include aziridination of alkenes, C–H amination, and transamination toward heteroatoms.^85^ In recent years, aziridination reactions utilizing various metal catalysts have been reported.^711−715^ There have also been numerous reports on the methodologies based on generating iodonium imide species in situ to perform these reactions.^716−723^ In addition to aziridination of olefins, aminocyclopropanation reactions of allenes are also known. Pérez and co-workers reported that the reaction of allenes **227**, alcohols, and iminiodanes in the presence of silver catalysts gave the corresponding aminocyclopropanated compounds **228** in moderate yields (Scheme 83).^713^ The structures of the products have been determined by X-ray crystallography. The authors have also reported that, under similar conditions, the reaction without the alcohol yields azetidine when the substrate is an aromatic allene, while the reaction with an aliphatic allene yields methylene aziridine.

**Scheme 84:**
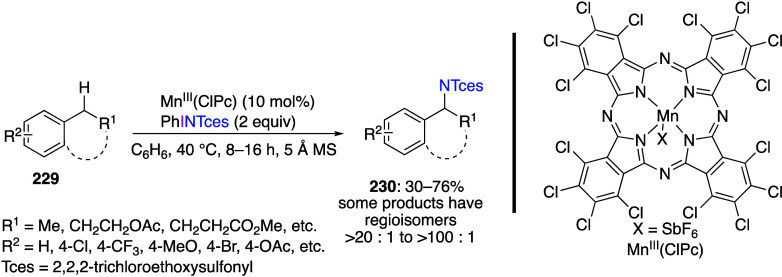
Selective Benzylic C–H Amination of **229**

Several examples of C–H amination reactions using iodonium imides with various metal catalysts have been investigated, and stereoselective and regioselective aminations have been reported.^724−728^ For example, White and co-workers reported a benzyl position selective C–H amination reaction utilizing Mn catalyst.^726^ The reaction of aromatic compounds **229** proceeds in the presence of Mn catalyst and iodine(III) reagent to give the corresponding C–H amination products **230** (Scheme 84). This reaction can also be used for C–H amination of compounds with various natural product skeletones. A characteristic feature of the selectivity of this amination is that when the substrate has an electron-rich benzyl position and an electron-deficient benzyl position, the electron-rich benzyl position is selectively aminated, and in amination reactions to secondary and tertiary benzyl positions, amination proceeds in a secondary position regioselectively. According to the results of deuterium experiments, the authors suggest that the rate-limiting step in this reaction is the C–H bond cleavage step. Recently, in addition to these C–H amination reactions, Pérez’s group has also reported Si–H amination reactions of silane compounds.^729^

**Scheme 85:**
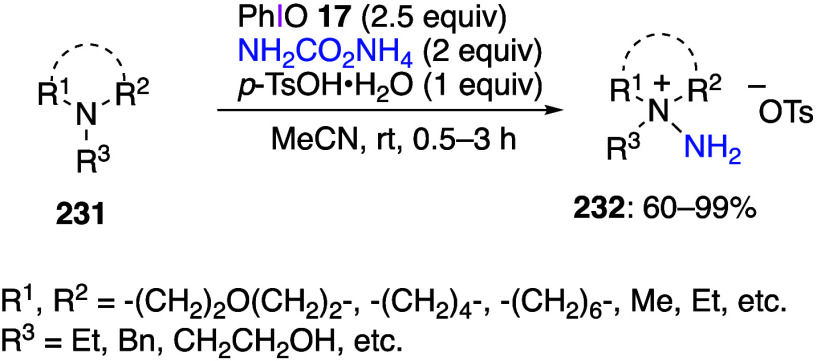
Preparation of Hydrazinium Tosylate Salts **232**

Transimination reactions have been investigated previously, and a variety of new methods have been reported very recently.^730−732^ Numerous reports on the imination reactions of sulfur atom using the in situ generated iodonium imide species from NH_2_CO_2_NH_4_,^733−739^ (NH_4_)_2_CO_3_,^740,741^ and NH_3_^742,743^ have recently been published. The generated PhI = NH species could exist for only a short time but has been successfully detected by HRMS. For example, Bull and Luisi reported that the reaction of a tertiary amine compound **231** with PhIO **17**, NH_2_CONH_4_, and TsOH efficiently produced a quaternary ammonium product **232** in which the NH_2_ group from NH_2_CONH_4_ was attached to the nitrogen atom of the amine (Scheme 85).^738^ The reaction is chemoselective and proceeds nitrogen-atom-selectively even if the substrate contains an oxygen atom or double bond. The authors believe that this reaction involves intermediate formation of PhI = NH species in the reaction system.

**Scheme 86:**
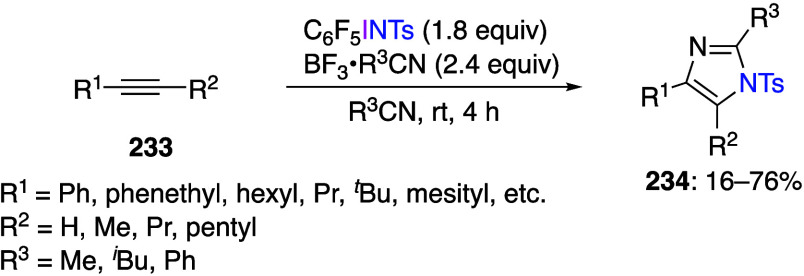
[2 + 2 + 1] Cycloaddition Reaction Using C_6_F_5_INTs

Reactions utilizing iminoiodane without the use of metal catalysts are known, and C–H amination and *trans*-imination reactions have been reported.^744−748^ Saito and co-workers reported the cycloaddition reaction using iminoiodanes without metal catalysts.^749^ This reaction was performed by adding C_6_F_5_INTs to an alkyne **233** in the presence of BF_3_ in nitrile solvent to give the corresponding imidazole **234** (Scheme 86).^749^ The authors synthesized a highly electrophilic iminoiodane, C_6_F_5_INTs, and found that this reagent has enhanced reactivity in this reaction.

**Scheme 87:**
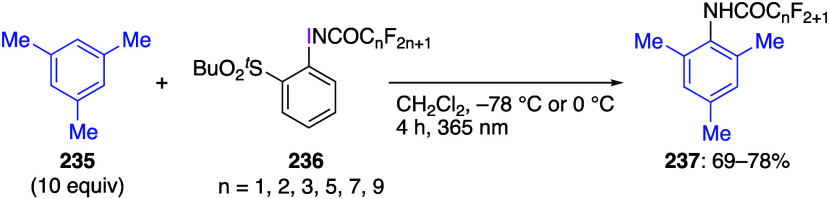
Photoinduced C–H Amination of Mesitylene **235** Using Reagent **236**

Reactions using iminoiodanes under light irradiation conditions^750−754^ or with photocatalysts^755^ have recently been reported. For example, Kobayashi and Takemoto reported the amination of various substrates by activation of iminoiodanes under photoirradiation.^750,751,754^*N*-Acyliminoiodanes and *N*-sulfonyliminoiodanes were used as the iodine reagents in the reaction. The *N*-acyliminoiodanes were previously reported to be unstable compounds,^756^ but the authors found that they could be stabilized by introducing a coordinating substituent at the *ortho*-position, and their structures were confirmed by X-ray crystallography. In the reaction with *N*-acyliminoiodane, mesitylene **235** reacted with the iodine(III) reagent **236** (*n* = 1) under photoirradiation at 365 nm to give the corresponding *N*-acylated compound **237** (*n* = 1) in 77% yield (Scheme 87).^751^ This reaction also proceeded with iodine reagents bearing perfluoroalkyl groups of different chain lengths to give the respective products in moderate yields. The reaction proceeds efficiently with anisoles and heteroarenes, including some bioactive compounds as substrates, while reactions with toluene, bromobenzene, and chlorobenzene did not afford the products **237**.

### 3.4 μ-Oxo-iodine(III) Compounds

**Scheme 88:**
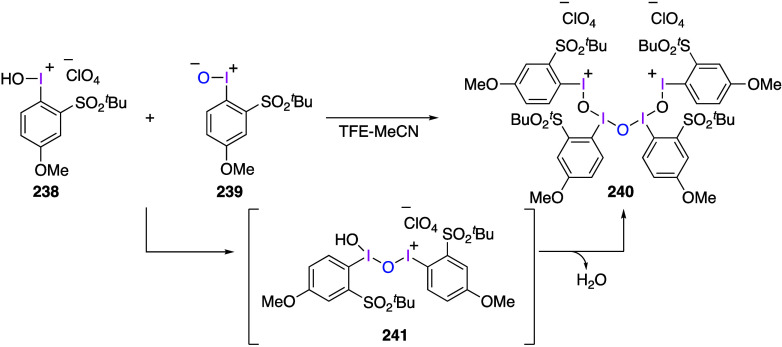
Steps in the Formation of Tetrameric Molecule **240**

Numerous representatives of μ-oxo-iodine(III) compounds, which are formed by bridging two hypervalent iodine moieties with oxygen atoms, have been synthesized and structurally investigated.^183^ In general, these compounds are more electrophilic and more reactive than their monomeric analogues because of the presence of the bridging oxygen.^757,758^ The general method of their synthesis consists of adding water to a monomeric hypervalent iodine reagent under stirring.^759^ Since 2016, several new iodine(III) compounds with μ-oxo structure have been synthesized, and their structures have been determined by X-ray crystallography.^258,343,760,761^ Recently, Powers and co-workers have found that crystallization of equal amounts of iodine(III) compounds **238** and **239** from a TFE-MeCN mixture yielded yellow crystals of μ-oxo-iodine(III) compound **240** with a tetrameric structure confirmed by X-ray crystallography (Scheme 88).^761^ This structure is formed by the elimination of water from dimer **241**, which is initially formed by condensation of **238** and **239**. The formation of **241** was confirmed by mass spectrometric analysis.

**Scheme 89:**
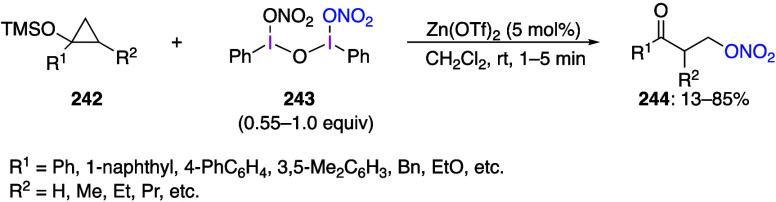
Nitrooxylation of Cyclopropyl Trimethylsilyl Ether **242**

Deng’s group has successfully synthesized μ-oxo-iodine(III) compounds with a nitrooxy group as a ligand, and their structures have been confirmed by X-ray.^258^ Two methods for synthesizing these compounds have been reported: (1) the reaction of DIB with nitric acid and (2) the reaction of silver nitrate with ArICl_2_; both methods yielded the corresponding μ-oxo-iodine(III) compounds in moderate to high yields. The authors have also investigated the reactivity of these compounds and reported that cyclopropyl trimethylsilyl ether **242** reacts with reagent **243** in the presence of a zinc catalyst to afford β-substituted ketones **244** in which the carbon–carbon bond in the original cyclopropane ring was cleaved (Scheme 89). When TEMPO is added as an additive, the yield of the target product is low and the product due to the combination of the substrate and TEMPO is formed. From these results, the authors suggest that the radical species may be involved in this reaction. They also evaluated the reaction mechanism by computational methods. In the reaction using β-ketoesters and β-keto amides as substrates, the corresponding α-nitrooxylated compounds were efficiently obtained.

### 3.5 Iodine(III) Compounds with Sulfonyloxy Ligands

#### 3.5.1 [Hydroxy(sulfonyloxy)iodo]arenes

**Scheme 90:**
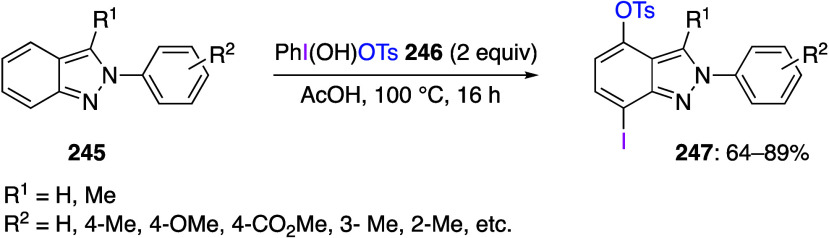
Difunctionalization of 2*H*-Indazole **245**

[Hydroxy(sulfonyloxy)iodo]arenes can be easily synthesized by ligand exchange reactions from [bis(acyloxy)iodo]arenes such as DIB and the corresponding sulfonic acid, most commonly, *p*-toluenesulfonic acid.^24,762^ These reagents are not only efficient oxidants for various substrates^763−768^ but also useful sulfoxyloxylating agents.^769−771^ Furthermore, a difunctionalization reaction of iodination-sulfoxyloxylation has recently been reported.^772^ The reaction of 2*H*-indazole **245** and [hydroxyl(tosyloxy)iodo]benzene (HTIB) **246** in acetic acid solvent under heating affords the corresponding disubstituted 2*H*-indazole compound **247**, which is C-4-sulfonyloxylated and C-7-iodinated (Scheme 90). This reaction proceeds with very high regioselectivity, producing products **247** in generally high yields. Similar reactions of iodine(III) reagents with various other sulfonyloxy groups produce respective products in moderate to high yields.

**Scheme 91:**
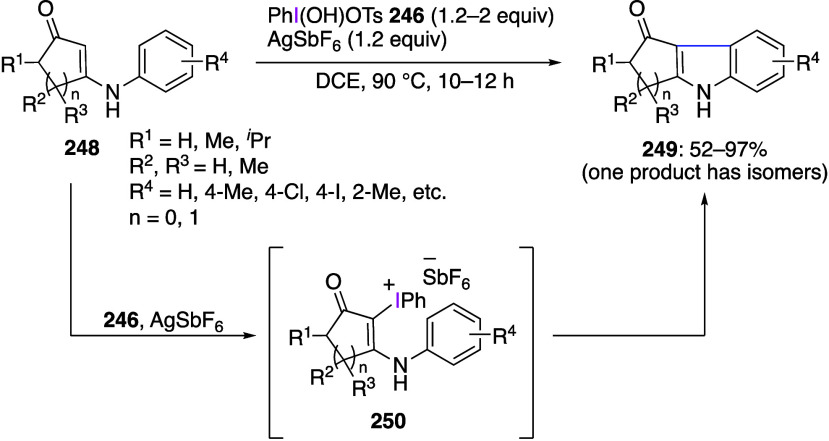
Synthesis of Carbazolones **249** from Exocyclic β-Enaminone **250**

Intramolecular cyclization reactions of HTIB leading to the formation of carbon-heteroatom^773−776^ and carbon–carbon bonds^777,778^ have been reported. For example, Das and co-workers reported that the reaction of exocyclic β-enaminones **248** with HTIB **246** in the presence of AgSbF_6_ leads to the formation of the corresponding carbazolones **249** via intramolecular carbon–carbon bond formation (Scheme 91).^776^ In the reaction of an exocyclic β-enaminone with 2-aminopyridine as a substituent, an intramolecular carbon-heteroatom bond formation occurs to yield the corresponding imidazo[1,2-*a*]pyridine. The authors suggest that the rate-limiting step in this reaction is not the aromatic C–H bond cleavage because the deuterium labeling experiments did not reveal any significant kinetic isotope effects. The presence of AgSbF_6_ is important because the generated iodonium salt **250** with the hexafluoroantimonate counterion acts as a photoinitiator and initiates a free radical reaction via the radical-cation pathway.

**Scheme 92:**
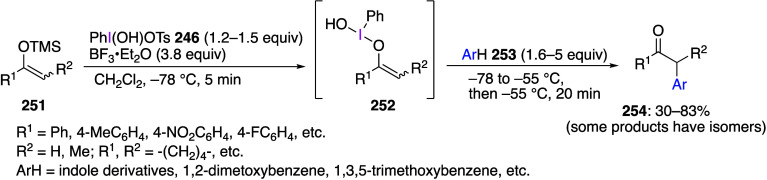
α-Arylation of Ketones **254** via Enolonium Species **252**

Szpilman’s group has reported the reaction of various substrates with enolonium species generated from mixing silyl enol ethers with HTIB reagents.^779−782^ For example, silyl enol ether **251** reacts with HTIB **246** in the presence of BF_3_·Et_2_O to form enolonium species **252**, and then an aromatic compound **253** was added to the reaction to give the corresponding α-arylated ketone **254** (Scheme 92).^781^ When one more molecule of silyl enol ether was added instead of the aromatic compound, the corresponding 1,4-diketone was obtained. The authors confirmed the presence of enolonium species formed in this reaction by NMR studies.^782^ The same group also reported that the reaction of vinyl azide as the starting material instead of silyl enol ether with HTIB leads to the formation of an analogue of the enolonium species, the azide-enolonium species, which reacts with a nucleophile to give the corresponding α-keto compound.^783^

#### 3.5.2 [Cyano(trifluoromethylsulfonyloxy)iodo]arenes

[Cyano(trifluoromethylsulfonyloxy)iodo]benzene, PhI(CN)OTf, can be conveniently synthesized by reacting iodosylbenzene or DIB with TMSOTf and TMSCN at low temperature.^51,784^ This reagent was originally reported more than 30 years ago, but its structure was not reliably established. Recently, Dutton and co-workers have revealed its X-ray structure.^186^ In particular, the authors found that the I–O bond distance in PhI(CN)OTf is longer than that of the known partially ionic PhI(OAc)OTf. In addition, ^19^F NMR studies indicated that the triflate signal in this compound is close to the signal of triflate anion, suggesting that PhI(CN)OTf is an ionic compound.

**Scheme 93:**
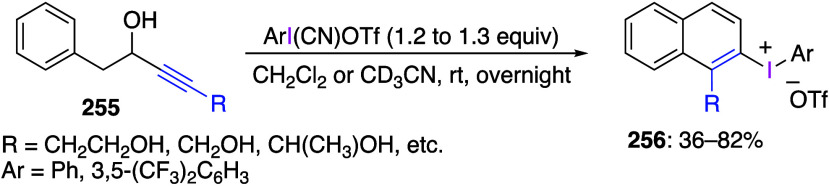
Cyclization of Propargylic Alcohols **255** to Naphthyl(aryl)iodonium Triflates **256**

ArI(CN)OTf reagents are commonly used in the synthesis of various iodonium salts.^785,786^ For example, Hinkle and Pike have reported the cyclization reaction of propargylic alcohols **255** with ArI(CN)OTf, leading to the corresponding naphthyl(aryl)iodonium thiflates **256** in low to good yields (Scheme 93).^786^ The authors found from X-ray structural analysis that the hydroxyl group of the fragment R in product **256** is coordinated to the iodine center.

**Scheme 94:**
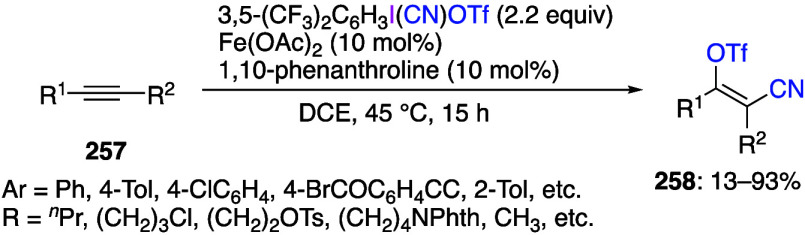
Cyanotriflation of Alkynes **257**

[Cyano(trifluoromethylsulfonyloxy)iodo]arenes can also be used as cyano-trifluorosulfonyloxylating reagents.^787^ Studer’s group has reported that the reaction of alkynes **257** with 3,5-(CF_3_)_2_C_6_H_3_I(CN)OTf in the presence of iron catalyst can regioselectively afford cyano-triflated products **258** in low to good yields (Scheme 94). These reactions are synthetically useful because they can convert disubstituted alkynes to tetrasubstituted alkenes in one step, and the OTf moiety of the obtained products can be further converted by appropriate treatment.

#### 3.5.3 [Bis(sulfonyloxy)iodo]arenes

**Scheme 95:**
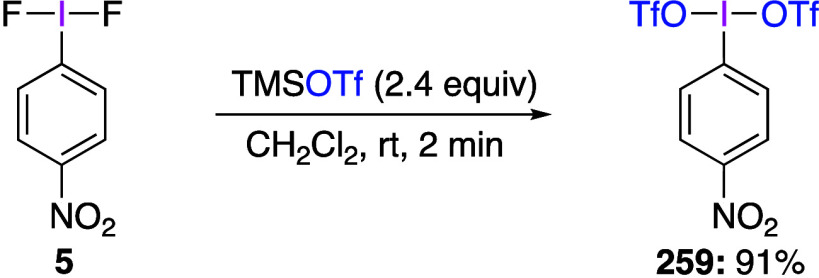
Preparation of *p*-NO_2_C_6_H_4_I(OTf)_2_**259**

The existence of [bis(sulfonyloxy)iodo]arenes was suggested long ago; however, their structures were not confirmed because of the high reactivity and low stability of these compounds.^187,191^ In particular, the generation of PhI(OTf)_2_ in situ has been utilized in many papers, but no identification data was provided.^453,788^ Recently, Dutton and co-workers have reported the first successful preparation of *p*-NO_2_C_6_H_4_I(OTf)_2_**259** from *p*-NO_2_C_6_H_4_IF_2_**5** and 2 equiv of TMSOTf (Scheme 95), and its structure was established by X-ray crystallography.^189^ The length of the I–O bond was found to be shorter than that of the reported I-OTf of PhI(OAc)OTf. The authors also found that *p*-NO_2_C_6_H_4_I(OTf)_2_**259** peaks at a downfield compared to PhI(OAc)(OTf) in the fluorine NMR, suggesting that the I–O bond of *p*-NO_2_C_6_H_4_I(OTf)_2_**259** is more associative than that of PhI(OAc)(OTf). The compound was highly unstable and decomposed at room temperature, but not under a nitrogen atmosphere at 150 K.

**Scheme 96:**
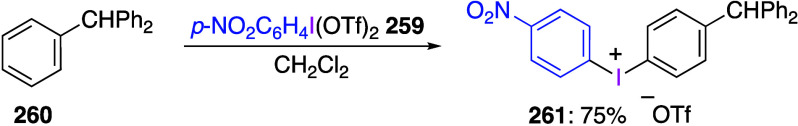
Reaction of Triphenylmethane **260** with *p*-NO_2_C_6_H_4_I(OTf)_2_**259**

The same authors have found that the reaction of compound **259** with triphenylmethane **260** yields diaryliodonium triflate **261** in 75% yield (Scheme 96).^789^ The control reaction using PhI(OAc)(OTf) instead of **259** did not proceed, indicating the higher reactivity of *p*-NO_2_C_6_H_4_I(OTf)_2_**259**. According to NMR studies, the reaction of compound **259** with cycloheptatriene yields tropylium triflate and the reaction with adamantane gives adamantyl triflate. Furthermore, the reaction of **259** with 1-fluoroadamantane affords *p*-NO_2_C_6_H_4_I(F)(OTf). From this result, the authors also suggested that compound **259** can act as a Lewis acid.

### 3.6 Iodine(III) Compounds with Carbon Ligands

#### 3.6.1 Iodine(III) Compounds with Trifluoromethyl Ligand

**Scheme 97:**
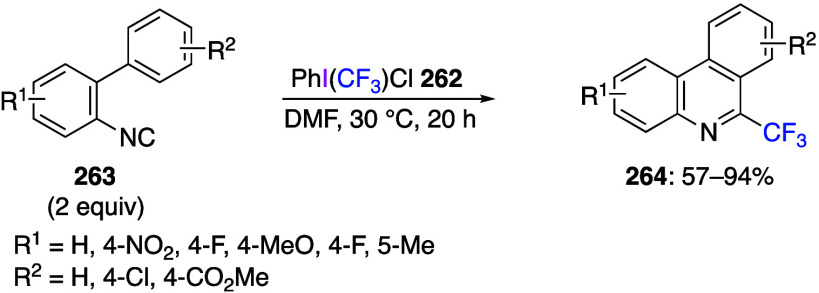
Trifluoromethylation-Cyclization of Isocyanides **263** with PhI(CF_3_)Cl **262**

Acyclic hypervalent iodine compounds with trifluoromethyl groups as ligands are usually unstable at room temperature. The formation of [PhICF_3_]^+^-species in solution was confirmed by mass spectrometry,^790^ and X-ray structures of pseudocyclic trifluoromethyl-substituted iodanes were previously reported.^791^ More recently, in 2018, Wang and co-workers reported the preparation of PhI(CF_3_)Cl in high yields by adding TMSCF_3_ to PIFA and treating with NaCl.^792^ X-ray structural data indicated a significant ionic character of the I–Cl bond in this compound. This compound can work as a CF_3_-introducing reagent for various organic substrates.^793−800^ For example, PhI(CF_3_)Cl **262** reacts with aromatic isocyanides **263**, forming trifluoromethylation and cyclization products **264** in good yields (Scheme 97).^800^

**Scheme 98:**
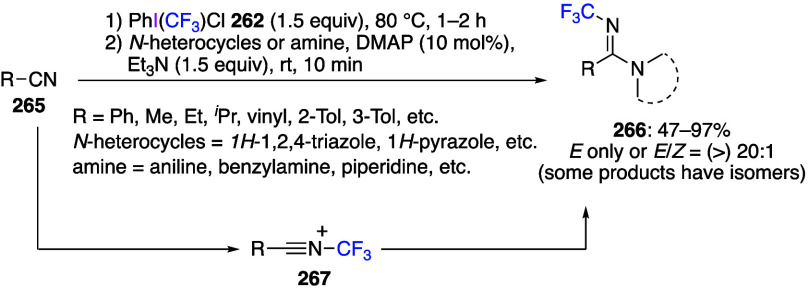
Synthesis of *N*-Trifluoromethyl Amidines **266** from Nitriles Using PhICF_3_Cl **262**

It has been reported that this reagent can transfer CF_3_ to a carbon atom as well as to a nitrogen atom. For example, the reaction of PhI(CF_3_)Cl **262** with nitriles **265** and a nitrogen nucleophile leads to *N*-trifluoromethylation and amination of the nitrile to give the corresponding amidine products **266** (Scheme 98).^794^*N*-Heterocycles as well as cyclic and acyclic amines can be used as nitrogen-based nucleophiles in this reaction. Other than nitrogen nucleophiles, alcohols and thiols can also be used as nucleophiles; in this case, the corresponding imidate and thioimidate products are obtained. It was suggested that these reactions involve initial interaction of nitrile **265** with the iodine reagent to form *N*-CF_3_ nitrilium ions **267**, followed by the reaction of a nucleophile to convert it to the final product. It has been recently reported that when methylene isocyanides are used as nucleophiles, the trifluoromethylation of the nitrile is followed by a cyclization reaction to yield the corresponding *N*-CF_3_ imidazoles.^793^

**Scheme 99:**
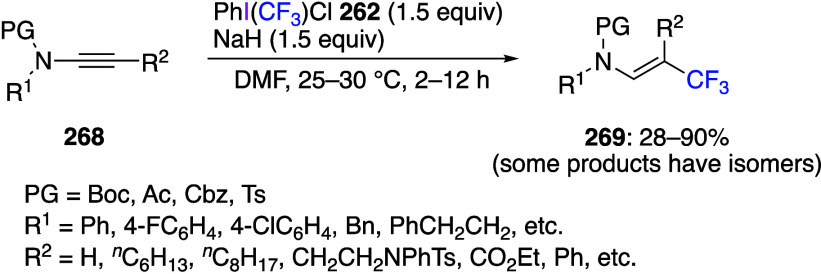
Hydrotrifluoromethylation of Ynamides **268**

PhI(CF_3_)Cl can also be used for the addition of trifluoromethyl groups to alkynes and alkenes. For example, it has been reported that the reaction with alkynes **268** with PhI(CF_3_)Cl **262** in the presence of NaH leads to the formation of alkenes **269** with *syn* addition of hydrogen and a trifluoromethyl group (Scheme 99).^795^ In the presence of TEMPO this reaction did not yield alkenes **269**, which suggests that radicals are involved in the mechanism. Deuterium experiment indicated that the hydrogen atom for the conversion of alkynes to alkenes comes from DMF as the reaction solvent.

**Scheme 100:**
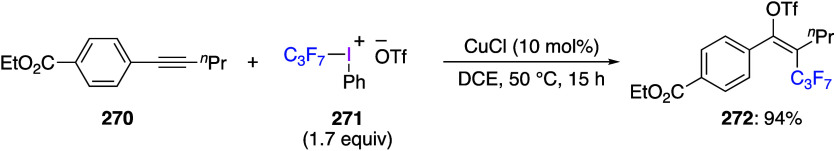
Regio- and Stereoselective Perfluoropropylation of Alkyne **270**

Reactions utilizing perfluoroalkyl-substituted iodanes have also been reported. For example, Studer and co-workers reported that perfluoropropyl-substituted hypervalent iodine reagent **271** reacts with aromatic alkyne **270** in the presence of a copper catalyst to give alkene **272** as a result of *anti*-addition (Scheme 100).^801^ A similar reaction can also be efficiently performed with iodine reagents having perfluorohexyl or perfluorooctyl group as a ligand. The authors sugested that this reaction proceeds via initial generation of perfluoroalkyl radical from the iodine reagent and copper catalyst.

#### 3.6.2 Iodine(III) Compounds with Diazomethyl Ligand

**Scheme 101:**
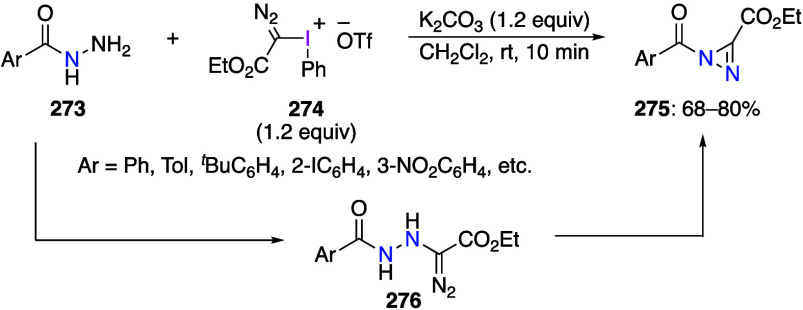
Diazirination of Carbohydrazides **273**

Hypervalent iodine compounds with a diazomethyl group as the ligand were originally synthesized nearly 30 years ago, and their structure was investigated by X-ray.^802^ These compounds can be used as reagents for transfer of the diazomethyl group to various substrates.^803^ For example, Singh and co-workers reported that the reaction of carbohydrazides **273** with reagent **274** under basic conditions yields diazirine compounds **275** (Scheme 101).^804^ Aromatic and aliphatic carbohydrazides can be used as substrates in this reaction. The reaction also proceeds with iodine reagents having ester moieties with various substituents. The authors suggested that the nitrogen atom of the carbohydrazide **273** initially reacts with reagent **274** to yield intermediate product **276** in which the diazomethyl group is transferred to the nitrogen atom, followed by intramolecular cyclization and oxidation to give the final product **275**. In addition to this reaction, transfer reactions of the diazomethyl group to nitrogen atom with various substituents have also been reported.^805,806^ Furthermore, transfer reactions to sulfur^807−809^ and phosphorus atoms^808^ are also known.

**Scheme 102:**
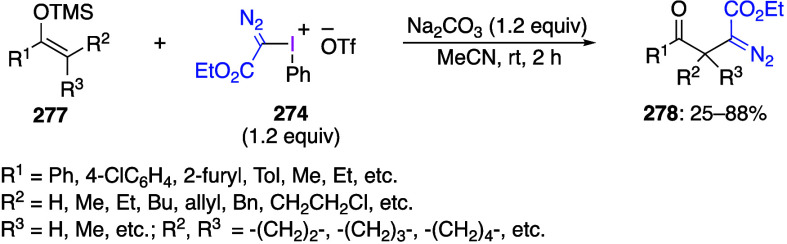
α-Diazomethylation of Silyl Enol Ethers **277**

The transfer reaction of the diazomethyl group to the carbon atom has also been reported. Suero and co-workers reported that reactions of silyl enol ethers **277** with reagent **274** under basic conditions afford β-diazocarbonyl compounds **278** (Scheme 102).^810^ They found that the reaction proceeds even when natural products and pharmaceuticals were used as substrates. The obtained β-diazocarbonyl compounds undergo stereoselective intramolecular cyclization using a rhodium catalyst to selectively afford 1,3-C–H-inserted cyclized compounds.

**Scheme 103:**
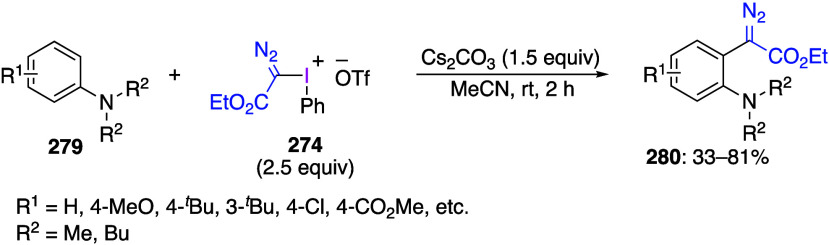
*ortho*-Diazomethylation of Aromatic Tertiary Amines **277**

Li and co-workers have also reported the reaction of aromatic tertiary amines **279** with reagent **274** to introduce the diazomethyl group at the *ortho*-position of the nitrogen (Scheme 103).^811^ The resulting products **280** can be converted to the corresponding indole compounds using a rhodium catalyst. The authors suggest that the diazomethyl radical species are generated in this reaction via the formation of an EDA complex between the amine and the iodine reagent. A method for generating diazomethyl radical species with a photocatalyst and iodine(III) reagent under LED irradiation is known, and reactions using these conditions have been reported.^812^

#### 3.6.3 Iodonium Ylides

**Scheme 104:**
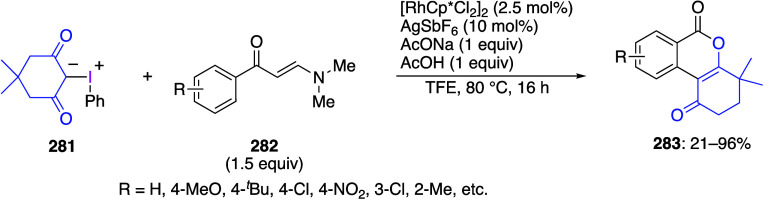
[3 + 3] Annulation of Enaminones **282** Using Iodonium Ylide **281**

Iodonium ylides are carbon analogues of iodonium imides with the carbon atom bonded to the iodine atom.^104,198^ Electron-withdrawing groups such as carbonyl and sulfonyl are usually used as the stabilizing substituents. Iodonium ylides can be synthesized by reacting DIB with a diketone or disulfonyl compound under basic conditions. Recently, Yoshino and Matsunaga reported a procedure in which aryl tin or germanium compounds are reacted with I(CO_2_CF_3_)_3_ to generate PIFA species in the reaction system, followed by the addition of a Meldrum’s acid derivative to produce the corresponding ylide.^813^ The same group also reported the synthesis of ylides using iodine triacetate.^814^ Structures of several new ylides were investigated by X-ray diffraction studies.^815−818^ The negatively charged carbon ligands in ylides in general have low nucleophilicity; however, they may act as nucleophiles toward highly electrophilic substrates.^819^ Numerous reactions involving the carbon ligand of iodonium ylide have been well investigated, including cyclopropanation,^820−824^ spirocyclization,^825−827^ and ylide transfer reactions.^828,829^ In recent years, heterocyclic ring annulation reactions utilizing iodonium ylides and various substrates have been reported. For example, Gao and Li reported a [3 + 3] cyclization reaction using iodonium ylide **281** and enaminone **282** in the presence of rhodium and silver catalyst, resulting in the corresponding isocoumarin products **283** (Scheme 104).^830^ Interestingly, this reaction does not proceed with acyclic iodonium ylides. The authors have suggested that the transition state in this reaction involves a C–H cleavage based on deuteration experiments. Similar reactions leading to isocoumarins have been reported by other groups.^831−833^ Hung and Yu reported that when ruthenium catalyst was used instead of rhodium catalyst in a similar reaction, a [3 + 2] cyclization occurs to afford the corresponding 3a,7a-dihydroxyhexahydro-4*H*-indol-4-one.^834^ Heterocyclic ring annulation reactions utilizing various substrates and iodonium ylides and resulting in [3 + 3],^835−839^ [3 + 2],^840−845^ [4 + 1],^846^ and [4 + 2]^835,847−855^ cyclizations have also been reported.

**Scheme 105:**
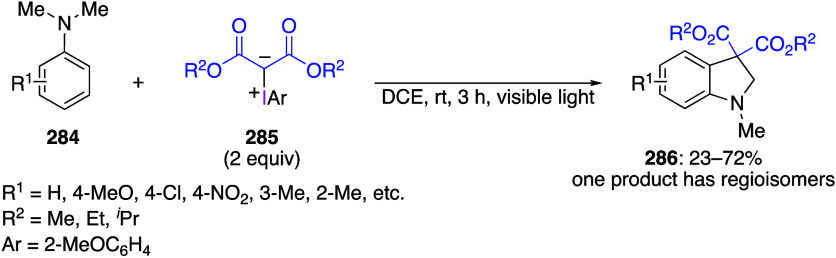
Cyclization of Aromatic Tertiary Amines **284** Using Iodonium Ylide **285**

While most of these reactions require transition metal catalysts, several publications report heterocyclic synthesis using single electron transfer methodology that does not require transition metal catalysts.^856−858^ For example, it has been reported that the reaction of acyclic aromatic tertiary amines **284** with iodonium ylide **285** in the presence of visible light irradiation proceeds as the [4 + 1] cyclization to give the corresponding indoline derivatives **286** (Scheme 105).^856^ It is also known that when cyclic aromatic tertiary amine compounds were used, *N*-phenylpyrrolidines could be obtained. In these reactions, the addition of TEMPO does not yield the desired product, while the addition of CCl_4_ yields a chlorinated compound derived from CCl_4_, indicating that radicals are involved in the reaction. The authors hypothesize that EDA complexes are formed with iodine(III) and amine reagents in the early stages of the reaction, and these complexes induce single-electron transfer under visible light. As well as cyclization reactions, such reactions using single-electron transfer reactions have also been used in three-component condensation reactions.^859,860^

**Scheme 106:**
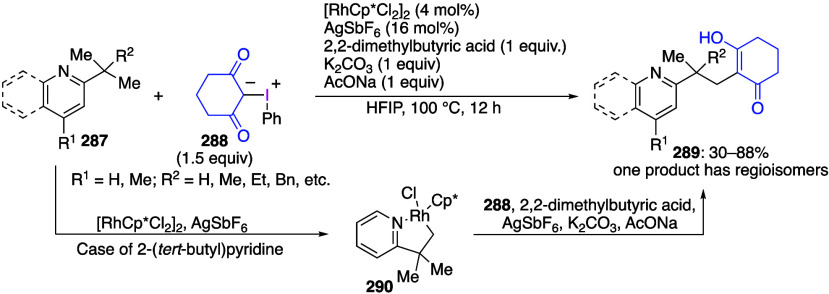
Unactivated C(*sp*^*3*^)–H Bond Insertion Reaction of Pyridines **287**

The insertion of iodonium ylide carbon ligands into C–H bonds has been investigated previously,^104^ and several new reports have been published recently.^861−864^ Jiang and Liu reported that the reaction of a pyridine compounds **287** with an inactive C(*sp*^*3*^)–H bond with a cyclic iodonium ylide **288** in the presence of rhodium and silver catalysts gives products **289** in which the ligand of the iodonium ylide is inserted into the C(*sp*^*3*^)–H bond (Scheme 106).^862^ The obtained products could be further converted into various derivatives under appropriate conditions. The authors found that the reaction of 2-(*tert*-butyl)pyridine with rhodium and silver catalysts in the absence of iodonium ylide produced a cyclic pyridine-rhodium complex compound **290**. The reaction of the obtained complex **290** with iodonium ylide **288** gave the desired product, implying that the pyridine-rhodium complex is an important key intermediate in the reaction.

**Scheme 107:**
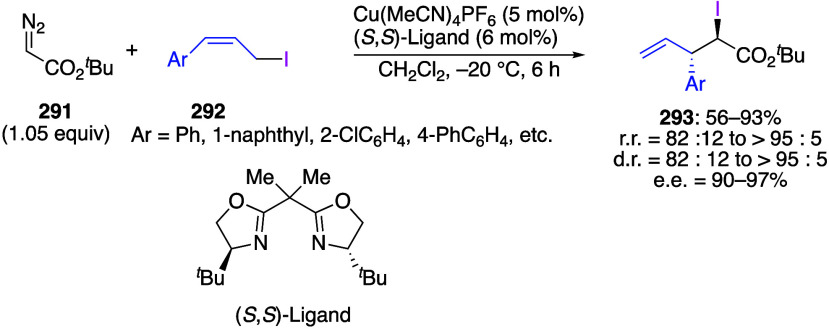
Copper-Catalyzed [2,3]-Sigmatropic Rearrangement

Several reactions based on the generation and utilization of iodonium ylides in situ have been reported.^865−868^ Ylide species can be generated by reacting an aliphatic monovalent iodine compound with a diazo compound. Tambar’s group has reported sigmatropic rearrangement reactions using this approach. Addition of *tert*-butyl α-diazo ester **291** to aryl-substituted allylic iodides **292** in the presence of copper catalysts leads to a [2,3]-sigmatropic rearrangement affording the corresponding terminal alkene products **293** with high regioselectivity (Scheme 107). This reaction proceeds efficiently even with polysubstituted allyl iodides.

**Scheme 108:**
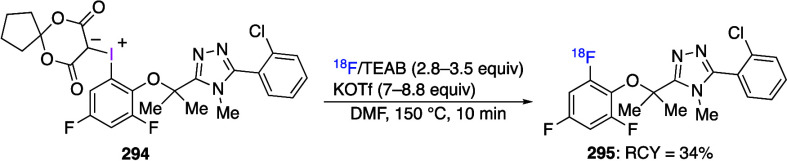
Radiofluorination of Iodonium Ylide **294**

The reactions of iodonium ylides resulting in nucleophilic substitution of the iodonium moiety in an aromatic ring have been reported. In particular, iodonium ylides are commonly used to introduce fluorine-18 radiolabeling into unactivated aromatic rings.^102,869,870^ In recent years, numerous syntheses of iodonium ylides with various substituents in the aromatic ring followed by fluorine-18 radiolabeling on the aromatic ring have been reported.^871−880^ For example, Li and Cai reported the synthesis and radiofluorination reaction of iodonium ylide **294** to give fluorinated product **295** with a radiolabeled *ortho*-position (Scheme 108). Reaction with iodonium ylide having an alkoxy group in the *para*-position also yields compounds with ^18^F in the *para*-position. Product **295** can be used as a radioactive tracer suitable for medical imaging of 11β -HSD1 in the primate brain. Several similar reactions leading to the functionalization of the aromatic ring of iodonium ylides have been reported.^881−883^

#### 3.6.4 Iodonium Salts

Iodonium salts are generally defined as hypervalent iodine(III) compounds with two carbon ligands, and their chemistry has been summarized in numerous review articles.^32,54,60,88,165,884,885^ The most typical reactions of iodonium salts result in a transfer of the carbon ligand to various nucleophilic organic substrates. Recent examples of the reactions of iodonium salts bearing alkynyl, alkenyl, alkyl, and aryl groups as the ligands are discussed in this section.

**Scheme 109:**
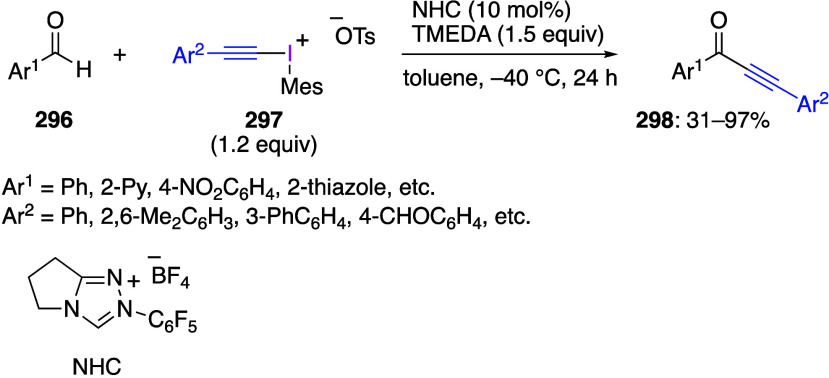
NHC-Catalyzed Synthesis of Ynones **298** from Alkynyliodonium Salts and Aldehydes

Alkynyliodonium salts can be conveniently synthesized by ligand exchange reactions between alkynyltin compounds and hypervalent iodine(III) reagents.^886^ Reactions of alkynyliodonium salts with various nucleophiles lead to a transfer of the alkynyl ligand yielding the corresponding substituted alkynes.^887−890^ The C–H bond insertion reactions using alkynyliodonium salts are also known.^891^ For example, Kalek and co-workers reported that the reaction of aldehydes **296** with alkynyliodonium salts **297** in the presence of an NHC catalyst yields ynones **298** in which the C–H bond of the aldehyde is replaced by the alkynyl group (Scheme 109). Based on ^13^C-labeling experiments and DFT calculations, the authors suggest that the alkynyl group insertion into the C–H bond of the aldehyde proceeds by direct substitution of iodine via a Breslow intermediate that occurs at the α-acetylenic carbon.

**Scheme 110:**
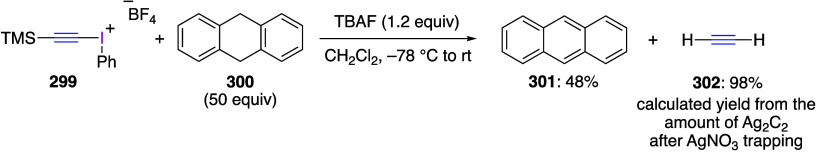
C_2_ Generation and Trapping Reaction in the Presence of 9,10-Dihydroanthracene **300**

**Scheme 111:**
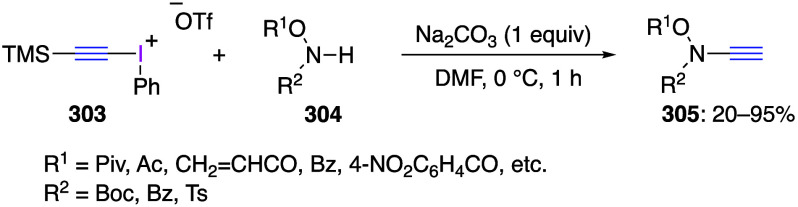
Reaction of Alkynyliodonium Salt **303** with *N*-(Acyloxy)sulfonamides **304**

Recent studies have demonstrated that alkynyliodonium salts can be used for the generation of diatomic carbon (C_2_). Miyamoto and Uchiyama reported that upon treatment of alkynyliodonium salt **299** with TBAF in the presence of 9,10-dihydroanthracene **300**, C_2_ is generated and trapped to form anthracene **301** and acetylene **302** (Scheme 110).^892^ They also reported that nanocarbons such as graphite, carbon nanotubes, and C_60_ can be produced by reacting alkynyliodonium salt **299** with a source of fluoride anion. The mechanism of C_2_ formation has been investigated by computational chemistry.^893−895^ Recently, a reaction utilizing this mehod of C_2_ generation was reported by Saito’s group.^896^ They reported that the reaction of alkynyl iodonium triflate **303** with *N*-(acyloxy)sulfonamide **304** under basic conditions gave the corresponding *N*-(acyloxy)-*N*-alkynylamide **305** (Scheme 111). The authors have also found that, in the presence of TEMPO or galvinoxyl, product **305** is not formed, which supports the reaction mechanism involving C_2_ reactive species.

**Scheme 112:**
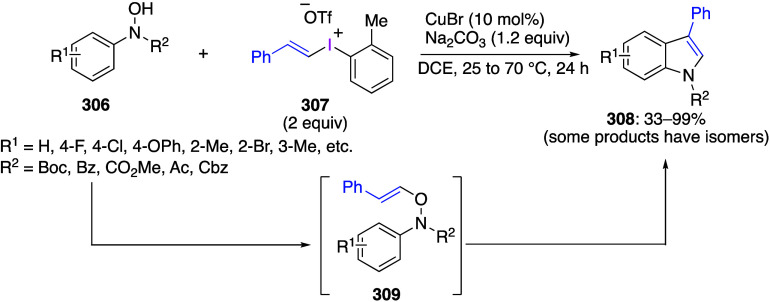
Synthesis of Indoles **308** from Arylhydroxylamines **306** and Vinyliodonium Salt **307**

Vinyliodonium salts are useful reagents for vinyl group transfer. Recently, a convenient synthesis of vinyliodonium salts by reactions of alkynes with iodine(III) reagents such as DIB or PhIO has been reported.^681,788,897^ The preparation of vinyliodonium salts from alkynyliodonium salts by addition reaction has also been reported.^898,899^ Vinyliodonium salts can transfer the vinyl group from iodine to carbon atoms^900^ and various heteroatoms.^785,901−906^ It has also been reported that these salts can be used for the transfer reactions to radioactive atoms.^907,908^ Gao and co-workers reported the vinyl group transfer reaction to oxygen atoms of arylhydroxylamines.^902^ The reaction of arylhydroxylamines **306** with vinyliodonium salt **307** under basic conditions and in the presence of a copper catalyst affords the corresponding indoles **308** (Scheme 112). The authors suggest that the reaction proceeds by the vinylation to oxygen atoms of arylhydroxylamines to give *O*-vinyl-*N*-arylhydroxylamines **309**, followed by several steps involving [3,3]-sigmatropic rearrangements leading to final product. The resulting compounds **308** can be converted to other indole derivatives by appropriate treatment. In addition to the vinyl group transfer reactions to various atoms, vinyl group insertion reactions into C–H bonds have also been reported.^909−913^

**Scheme 113:**
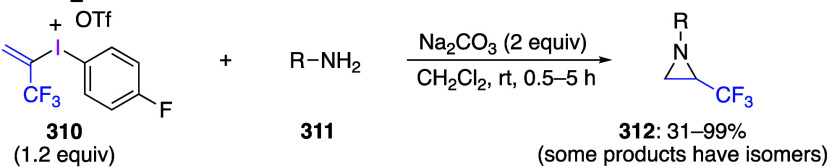
Aziridination of Amines **311** Using Vinyliodonium Salt **310**

It has been reported that vinyliodonium salts can be used for cyclization reactions as well as the addition of vinyl groups to substrates. For example, Stirling and Novák reported an efficient two-step synthesis of vinyliodonium salts from vinyl iodides and the aziridination reaction using them.^914^ In this reaction, reagent **310** and an amine **311** are combined under basic conditions to give the corresponding aziridine **312** (Scheme 113). The authors have also proposed the reaction mechanism. The suggested mechanism involves initial nucleophilic addition of amine to the β carbon of the vinyl moiety, followed by cyclization to the aziridine ring.

**Scheme 114:**
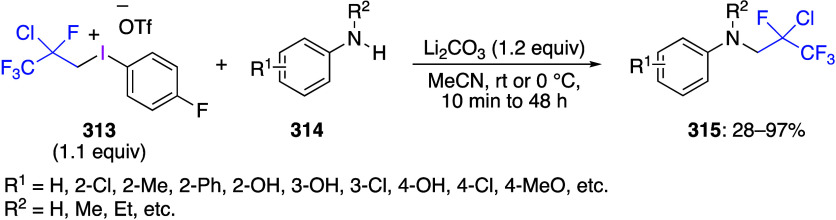
*N*-Alkylations of Anilines **314** Using Alkyliodonium Salt **313**

Several representatives of alkyliodonium salts stabilized by electron-withdrawing substituents on the alkyl group were previously known, and several new such compounds have been synthesized and investigated recently.^885^ Various reactions of alkyliodonium salts have been studied, including the transfer of alkyl groups to various heteroatoms^915−917^ and the insertion of alkyl groups into C–H bonds.^918−921^ For example, Stirling and Novák reported the synthesis of a new alkyliodonium compound **313** from 3-chloro-1,1,1,2-tetrafluoro-2-iodopropane via a dyotropic rearrangement. and its structure was established by X-ray crystallography.^922^ The authors have also found that the reaction of compound **313** with anilines **314** under basic conditions gave the corresponding alkylanilines **315** (Scheme 114). In addition to anilines, the authors have also reported *N*-alkylation reactions of heterocyclic compounds and C-alkylation reactions of indoles.

Diaryliodonium salts represent one of the most common classes of hypervalent iodine compounds with many practical uses. A broad variety of symmetric, nonsymmetric, and heterocyclic diaryliodonium compounds have been synthesized and utilized as reagents in organic synthesis.^32,54,60,98,884,923−925^ Diaryliodinium salts with various functional groups have been recently synthesized using two main approaches: (1) the reaction of aromatic compounds with hypervalent iodine(III) reagents or (2) the in situ oxidation of aryl iodides in the presence of aromatic compounds.^926−940^ Various counteranions have been introduced in the structure of diaryliodinium salts.^196,941−944^ Structures of numerous new diaryliodinium salts have been established by X-ray crystallography. The reactivity of these compounds as Lewis acids depending on the substituents in the aromatic ring has been investigated.^945,946^ Diaryliodonium salts are widely used as reagents for the arylation of nucleophilic carbon^947−958^ or heteroatoms^959−973^ in various substrates. The reactions of diaryliodonium salts with the corresponding nucleophiles are often used for the introduction of a radioactive label into an aromatic ring, such as the introduction of radioactive fluorine,^974−979^ bromine,^980^ and carbon.^981^

**Scheme 115:**
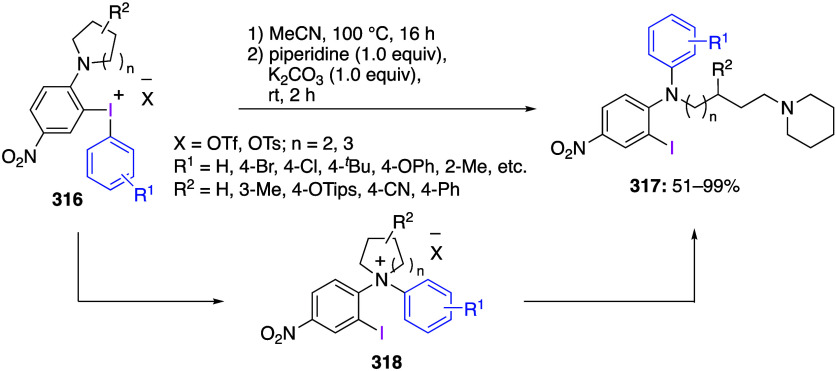
Synthesis of Anilines **317** from Diaryiodonium Salts **316**

Several rearrangement reactions resulting in the transfer of an aryl group in diaryliodonium species to a heteroatom at the *ortho*-position of the second aryl substituent have recently been reported.^982−985^ Olofsson and co-workers have reported that heating of a diaryliodonium salts **316** bearing a cyclic amino group in the *ortho*-position, followed by the addition of piperidine as a nucleophile under basic conditions, affords the corresponding anilines **317** (Scheme 115).^984^ The initial product of this reaction, quaternary ammonium salt **318** resulting from the rearrangement of iodonium salt **316**, can be isolated. Ammonium salts **318** can be efficiently converted to the final aniline products by adding piperidine or other nitrogen, halogen, and oxygen nucleophiles.

**Scheme 116:**
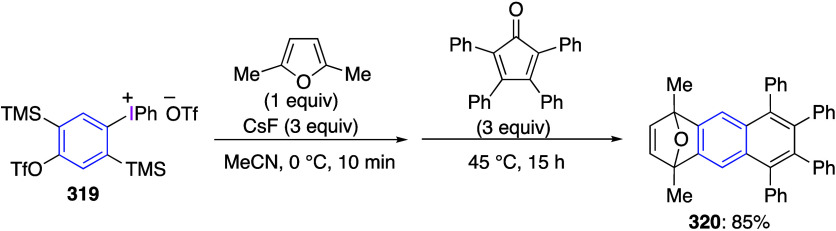
1,4-Double Cycloaddition of Diaryliodonium Triflate **318**

Diaryliodonium salts are commonly used as benzyne or aryne precursors.^100^ Recently, numerous examples of such reactions have been reported.^166,923,986−992^ Kitamura and his group have reported the synthesis and reactions of diaryliodonium salt that can consequently generate benzyne species at the 1- and 4-positions.^986^ For example, the reaction of [2,5-bis(trimethylsilyl)-4-(trifloxy)phenyl](phenyl)iodonium triflate **319** in the presence of CsF with 2,5-dimethylfuran as a first arynophile and tetraphenylcyclopentadienone as a second arynophile leads to a double cycloaddition reaction to give the corresponding product **320** in high yield (Scheme 116). The same group has also succeeded in synthesizing diaryliodonium salt, which can generate benzyne species at both the 1- and 3-positions, and reported the double cycloaddition reaction using this salt.

## 4 Cyclic Iodine(III) Compounds as Reagents

Cyclic hypervalent iodine compounds in general have higher thermal stability and modified reactivity compared to their acyclic analogues. The high thermal stability and lower reactivity of these compounds is explained by the link between apical and equatorial ligands at the iodine center, which restricts pseudorotation and reductive elimination of the iodoarene fragment.^993,994^ The vast majority of practically important cyclic hypervalent iodine reagents are based on the heterocyclic systems of benziodoxole or benziodazole. In recent years, various iodine-substituted benziodoxole derivatives have been prepared and utilized as reagents for transfer of the substituent on hypervalent iodine to organic substrate. The chemistry and synthetic applications of benziodoxoles and other hypervalent iodine heterocycles have been summarized in numerous recent reviews.^5,7,19,106,107^

### 4.1 Cyclic Iodine(III) Compounds with Halide Ligands

#### 4.1.1 Fluorobenziodoxoles and Fluorobenziodazoles

**Scheme 117:**
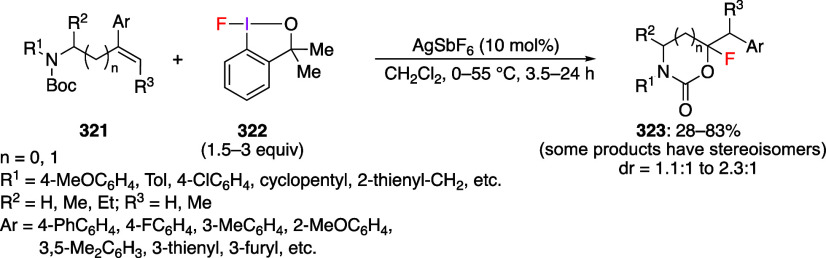
Intramolecular Fluorination of Carbamates **321** Using Fluorobenziodoxole **322**

Cyclic hypervalent iodine compounds with fluorine atoms as ligands belong to a relatively new class of iodine reagents originally synthesized about 10 years ago.^49^ A new synthesis of fluorobenziodoxoles from the corresponding aryl iodides and AgF_2_ was recently reported.^225^ Fluorobenziodoxoles are useful fluorinating reagents, similar to acyclic difluoroiodoarenes. In particular, numerous examples of fluorocyclizations of olefinic substrates using fluorobenziodoxoles have been reported.^995−1006^ Li and Lu have found that the reaction of fluorobenziodoxole **322** with an unsaturated carbamate **321** leads to an intramolecular fluorocyclization yielding fluorinated oxazolidin-2-ones **323** (Scheme 117).^1003^ The addition of TEMPO to the reaction reduces the yield of the products **323**, and the addition of BHT yields a BHT-substituted products, which suggest that radical species are involved in the reaction mechanism. Fluorobenziodoxole with a radioactive fluorine ligand can also be used in this reaction to introduce radioactive fluorine atom into the product.

**Scheme 118:**
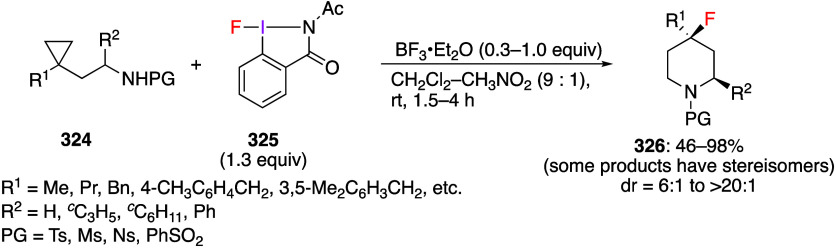
Ring Expansion Fluorination of Cyclopropanes **324** Using Reagent **325**

When a diazocarbonyl compound, instead of an olefin, is used as a substrate in the reaction with a hypervalent iodine reagent in the presence of an oxygen nucleophile, an intermolecular oxyfluorination reaction proceeds, yielding the corresponding fluorinated product.^1007−1009^ It has also been reported that, in the reaction of diazonium salts with a catalytic amount of iodine reagent and catalytic amount of BF_3_·Et_2_O, Baltz-Schemann reaction proceeds efficiently and the corresponding aromatic fluorinated compounds are obtained.^234^ Several other fluorination reactions of hypervalent iodine fluorides have been reported.^1010,1011^ For example, Zhang and co-workers reported that the reaction of cyclopropane derivative **324** with fluorobenziodazole **325** in the presence of BF_3_·Et_2_O leads to a ring-expanding fluorination reaction, resulting in the corresponding six-membered cyclic products **326** (Scheme 118).^1010^ The authors suggested that the active iodine species in this reaction is the complex of the iodine reagent and BF_3_, which was observed by NMR and mass spectrometry. The reaction mechanism was proposed using DFT calculations.

**Scheme 119:**
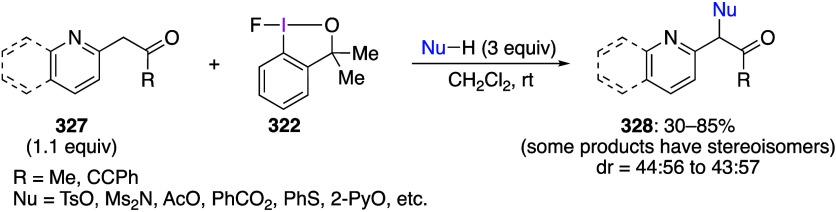
α-Functionalization of 2-Pyridyl Ketones **327**

Several reactions of fluorobenziodoxoles resulting in addition–elimination of fluorine have been reported.^999,1012,1013^ For example, Gulder and co-workers reported a reaction in which the addition of fluorobenziodoxole **322** in the presence of a nucleophile to 2-pyridyl ketones **327** leads to α-functionalized pyridyl ketone **328** (Scheme 119).^1013^ In this reaction, the α-position of the pyridyl ketone is first fluorinated, followed by a substitution reaction with a nucleophile to obtain the final product. In the absence of a nucleophile, the reaction yields the product of α-fluorination of the pyridyl ketone. The presence of the nitrogen atom in position 2 of the pyridyl ketone is essential; the product cannot be obtained by using a ketone substrate with a benzene ring instead of a pyridine ring or an isomer with a different position of nitrogen of the pyridine ring.

#### 4.1.2 Chlorobenziodoxoles and Chlorobenziodazoles

**Scheme 120:**
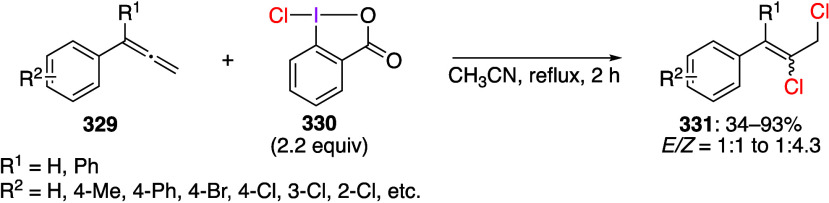
Dichlorination of Aromatic Allenes **329** Using Chlorobenziodoxole **330**

Chlorobenziodoxoles, cyclic iodine(III) derivatives with a chlorine atom ligand, are important chlorinating reagents for various organic substrates.^49^ Several new chlorination reactions using chlorobenziodoxoles have been reported recently.^1014−1018^ Murphy and co-workers reported that the reaction of aromatic allenes **329** with chlorobenziodoxole **330** yields products of dichlorination of the terminal double bond **331** with predominant (*Z*)-selectivity (Scheme 120). In the reaction of an aromatic allene with an alkyl group R^1^ at the α-position of the allene, a mixture of dichlorinated and monochlorinated products is formed. A similar reaction of an allene with (dichloroiodo)benzene is not selective, leading to several isomers of dichlorinated products.

**Scheme 121:**
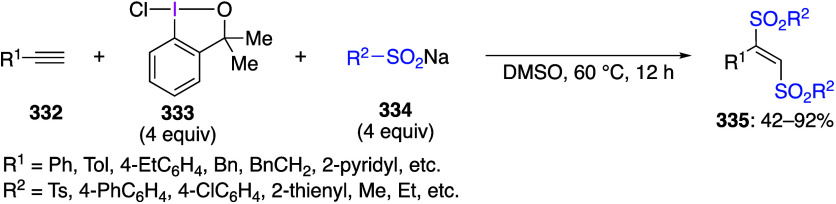
Disulfonation of Terminal Alkynes **332**

Chlorobenziodoxoles can also be used as selective oxidants of organic substrates.^1019−1022^ For example, the reaction of alkynes **332** with chlorobenziodoxole **333** and sodium sulfonates **334** efficiently yields 1,2-disulfonylethenes **335** with high (*E*)-selectivity (Scheme 121).^1020^ The authors believe that radicals are involved in this reaction because in the presence of TEMPO product **335** is not formed. They also assume that the initial products of ligand exchange between chlorobenziodoxole with sodium arylsulfinates are the active species in this reaction, as confirmed by ESI-MS spectrometry.^1021^

**Scheme 122:**
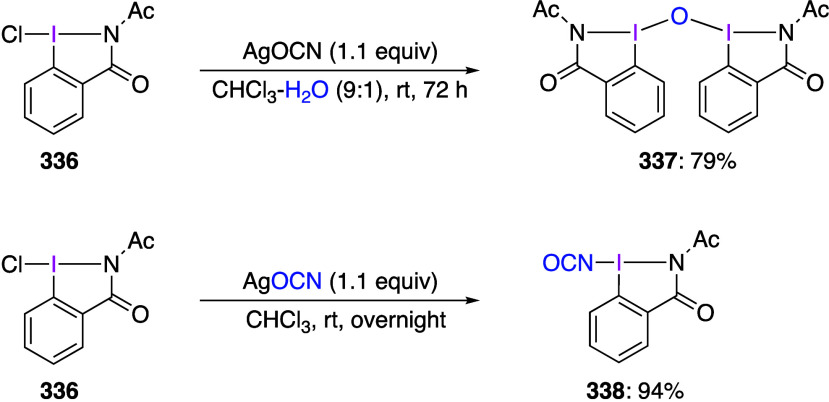
Ligand Exchange Reactions of Chlorobenziodazole **336**

Du and co-workers have found that the reaction of chlorobenziodazole **336** with silver cyanate in water and chloroform as cosolvents formed μ-oxo compound **337** (Scheme 122).^1023^ The authors have also reported that, in the absence of water, compound **338** with isocyanate ligand was obtained. Both structures were determined by X-ray crystallography.

### 4.2 Cyclic Iodine(III) Compounds with Oxygen Ligands

#### 4.2.1 Alkoxybenziodoxoles

**Scheme 123:**
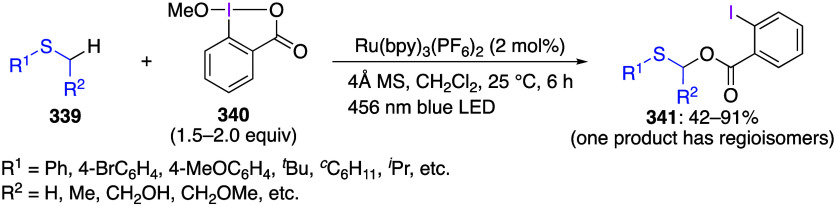
C–H Acyloxylation of Sulfides **339**

The acyclic hypervalent iodine compounds with alkoxy group ligands are relatively unstable and difficult to handle, whereas the cyclic derivatives, alkoxybenziodoxoles, are stable compounds. Alkoxybenziodoxoles can be prepared by a ligand exchange reaction of hydroxy- or acetoxybenziodoxoles with the corresponding alcohol,^1024^ and their structure has been established by X-ray analysis.^636,1025^ Recently, these compounds have been used as the reagents for C–H oxidation reactions.^213,1024,1026−1029^ For example, Chen and co-workers reported that sulfides **339** react with methoxybenziodoxole **340** in the presence of a photocatalyst under LED irradiation to form 2-iodobenzoic esters **341** via C–H acyloxylation reaction (Scheme 123).^1026^ This reaction proceeds efficiently not only with aryl- and alkyl-substituted substrates **339** but also with sulfur compounds with amino acid fragments as substituents. The C–H bond cleavage is the rate-determining step in this reaction, as confirmed by the reaction of deuterium-substituted substrate. The reaction most likely has a radical mechanism as supported by the experiments in the presence of TEMPO or BHT.

**Scheme 124:**
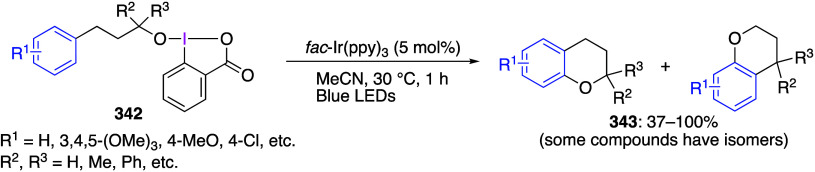
Photocatalyst-Induced Intramolecular Cyclization of **342**

Wallentin and co-workers prepared numerous alkoxybenziodoxoles by ligand exchange reactions between various alcohols and acetoxybenziodoxole, and reported C–H oxidation with these reagents.^1024^ When these alkoxybenziodoxoles **342** were treated with blue LED irradiation in the presence of a photocatalyst, an intramolecular cyclization reaction proceeded, resulting in the chromane products **343** (Scheme 124). In this reaction, the photocatalytic one-electron reduction of compound **342** generates the corresponding alkoxy radicals, followed by radical cyclization to yield the final products **343** along with 2-iodobenzoic acid as a by-product. This radical cyclization proceeds by competitive 1,5- and 1,6-addition reactions, resulting in the formation of two structural isomers.

**Scheme 125:**
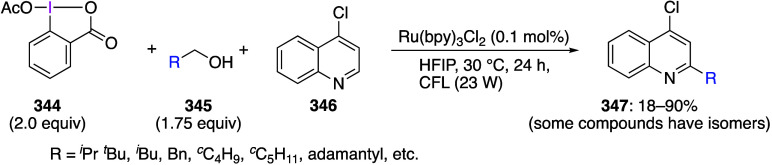
Photocatalyst Mediated C–H Alkylation of 4-Chloroquinoline **346**

A C–H alkylation reaction of heteroaromatic rings using in situ generated alkoxybenziodoxole has been reported.^1030^ In this reaction, cyclic acetoxybenziodoxole **344** reacts with various alcohols **345** and 4-chloroquinoline **346** in the presence of a photocatalyst and under irradiation with compact fluorescent lamps to form products **347** in which the 2-position of the quinoline ring is alkylated (Scheme 125). The reaction is also applicable to the substrates derived from natural products. The authors were able to isolate the initially formed alkoxybenziodoxole and demonstrated that it can be directly used in this reaction.

#### 4.2.2 Acyloxybenziodoxoles

**Scheme 126:**
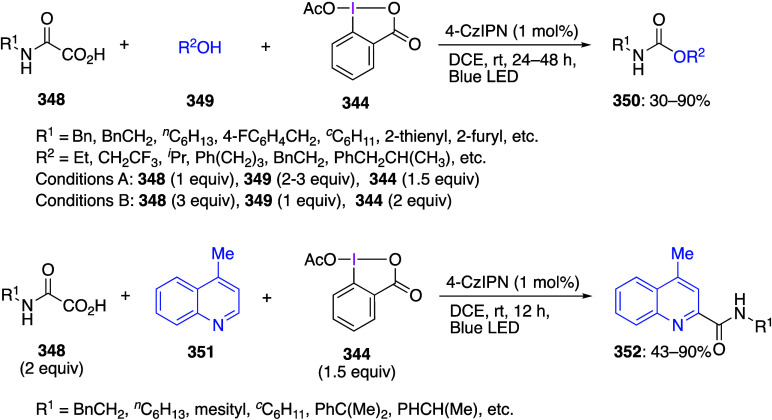
Radical Reactions of Oxamic Acid **348** Using Acetoxybenziodoxole **344**

Acyloxybenziodoxoles are usually prepared by ligand exchange reactions between hydroxybenziodoxoles or acetoxybenziodoxoles and carboxylic acids, and the structures of several acyloxybenziodoxoles have been determined by X-ray crystallography.^1030,1031^ Preparation of numerous acyloxybenziodoxoles using various carboxylic acids has been reported.^1031−1043^ Acyloxybenziodoxoles can be formed in situ in the reaction system by ligand exchange reactions and used for the generation of acyloxy radicals via radical cleavage of the I–OCOR bond under reaction conditions. Further decarboxylation of the acyloxy radicals can produce alkyl radicals. The radical species generated in this process then undergoes various inter- and intramolecular reactions. As an example of the intermolecular reaction, Cramail and Landais reported that acetoxybenziodoxole **344** reacts with oxamic acid **348** and alcohol **349** under blue LED irradiation in the presence of a photocatalyst, leading to a decarboxylation reaction of oxamic acid and forming urethane products **350** (Scheme 126).^1037^ It was reported that the addition of amines instead of alcohols yields the corresponding ureas. The proposed mechanism of this reaction involves initial formation of the carbamoyl radicals followed by their reaction with alcohols or amines to give final products. This mechanism was confirmed by additional experiments with TEMPO additives. The same group reported that when 4-methylquinoline **351** was used instead of alcohol in this reaction, products **352** with a carbamoyl group introduced at the 2-position were obtained.^1036^ Other than 4-methylquinoline heterocycles can be used in this reaction to give the corresponding products regioselectively and in high yield. Recently, the same group has reported an example of a similar reaction using Os(bptpy)_2_(PF_6_)_2_ instead of 4-CzIPN as a photocatalyst under near-infrared light irradiation.^1034^

**Scheme 127:**
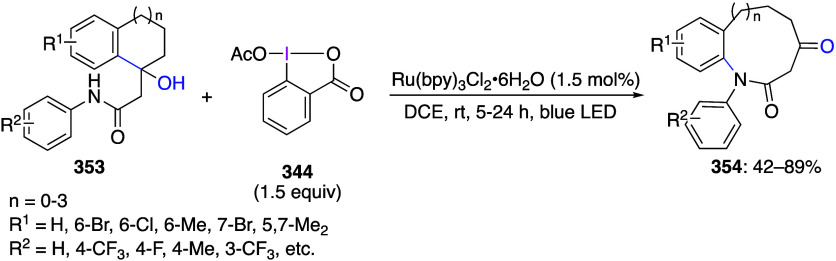
Intramolecular Cyclization of Cyclic Alcohols **353**

**Scheme 128:**
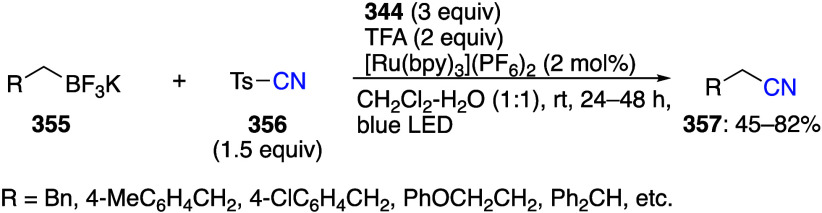
Cyanation of Alkyltrifluoroborates **355**

Several intra-^1044,1045^ and intermolecular reactions^1046−1048^ have been reported using one-electron transfer process between excited photocatalyst and acetoxybenziodoxole. An intramolecular cyclization occurs in the reaction of acetoxybenziodoxole **344** with cyclic alcohols **353** bearing an anilide moiety in the presence of [Ru(bpy)_3_Cl_2_·6H_2_O] as a photocatalyst under blue LED irradiation and leading to the medium-sized ring lactams **354** (Scheme 127).^1045^ This reaction also proceeds with heterocycle-substituted alcohols as substrates, resulting in the corresponding lactam products. Additional experiments in the presence of TEMPO suggested that this reaction proceeds via amidyl radicals. As an example of an intermolecular reaction utilizing a similar combination of a photocatalyst and acetoxybenziodoxole, a reaction of primary alkylborates **355** with TsCN **356** leading to cyanoalkanes **357** was reported (Scheme 128).^1048^ It was also reported that the reaction of alkylborates with pyridine-*N*-oxides instead of TsCN yields alkylated pyridine-*N*-oxides.^1046^

Acyloxybenziodoxoles and their derivatives have also been used as coupling reagents for various substrates.^1049−1051^

### 4.3 Cyclic Iodine(III) Compounds with Nitrogen Ligands

#### 4.3.1 Azidobenziodoxoles and Derivatives

**Scheme 129:**
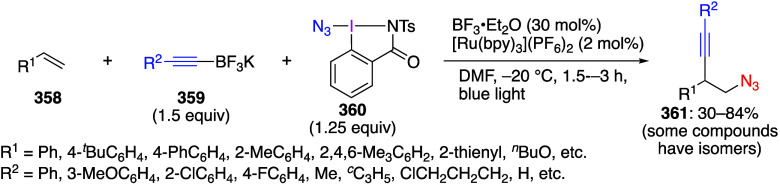
Azido-Alkynylation of Alkenes **359**

Preparation, structure, and reactivity of azidobenziodoxoles were originally reported about 30 years ago.^1052,1053^ The chemistry of these compounds was recently summarized in several specialized reviews.^7,19,111^ These compounds have been used as efficient reagents for transfer of the azido groups to various organic substrates.^7,107^ Some azidobenziodoxoles have low thermal stability and can be sensitive to friction and shock. To improve the stability of these reagents, substituents can be introduced into the aromatic ring,^1054^ or the iodoxole ring can be modified with various functional groups.^1055^ The stability of azidobenziodoxoles has also been investigated by computational analysis.^207,1056^ Typical reactions of azidobenziodoxoles include the following: azide addition to unsaturated compounds,^1057−1068^ azidation of C–H bonds,^1069−1082^ and application as HAT reagents utilizing the generated azido radical species.^1083−1085^ Waser and co-workers reported that reactions of alkenes **358** and alkynyl borates **359** with azidobenziodazole **360** under blue light irradiation in the presence of a photocatalyst yield products **361** in which the azido and the alkynyl groups are regioselectively bonded to the alkene (Scheme 129).^1057^ Proposed mechanism of this reaction includes initial generation of azido radicals by interation of the photocatalyst excited by blue light reacting with the iodine reagent, followed by regioselective addition to the olefin. When diphenyl phosphate is used in place of the alkyne, the corresponding product is obtained in moderate yield. The authors also noted that the azide moiety of the obtained compound can be efficiently converted to an amine by reduction.

**Scheme 130:**
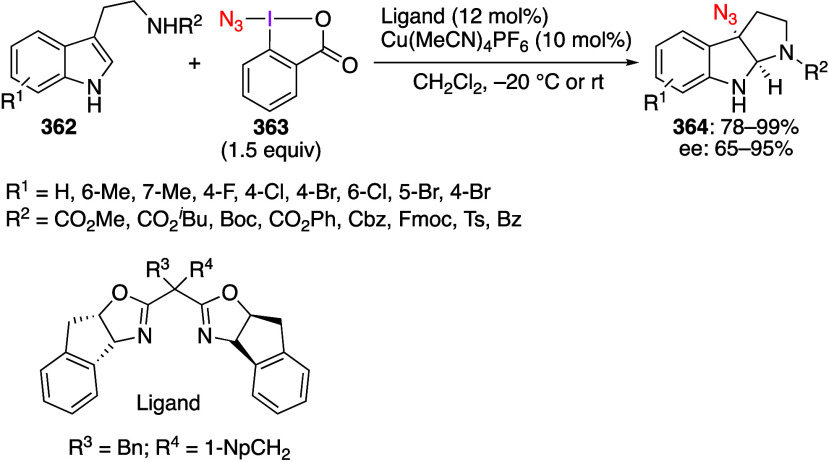
Dearomatic Azidation-Cyclization of Triptamines **362**

Azidobenziodoxoles can also be used in intramolecular azidation-cyclization reactions. For example, Cao and Deng reported that the reaction of tryptamines **362** with azidobenziodoxolone **363** in the presence of a copper catalyst and a catalytic amount of a chiral ligand leads to a dearomatic asymmetric azidation-cyclization reaction to afford the corresponding tricyclic products **364** with high enantioselectively (Scheme 130).^1066^ The authors believe that azide-Cu intermediates are initially formed from the iodine reagent and copper catalyst, and further reaction proceeds via radical addition of active azido radicals generated from these intermediates to tryptophan. Addition of various radical scavengers to the reaction suppressed the formation of products **364**, confirming the involvement of radical species in the reaction mechanism.

**Scheme 131:**
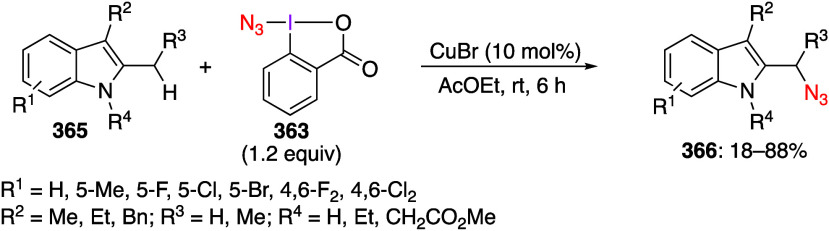
Selective C–H Azidation of Indoles **365**

Selective azidation of the alkyl group attached to the 2-position of the indole ring using azidobenziodoxoles has been reported. For example, the reaction of indole derivatives **365** with azidobenziodoxole **363** in the presence of a copper(I) catalyst provides products **366** that are selectively azidated in the alkyl group (Scheme 131).^1082^ A similar azidation occurs with tetrahydrocarbazole as a substrate.

**Scheme 132:**
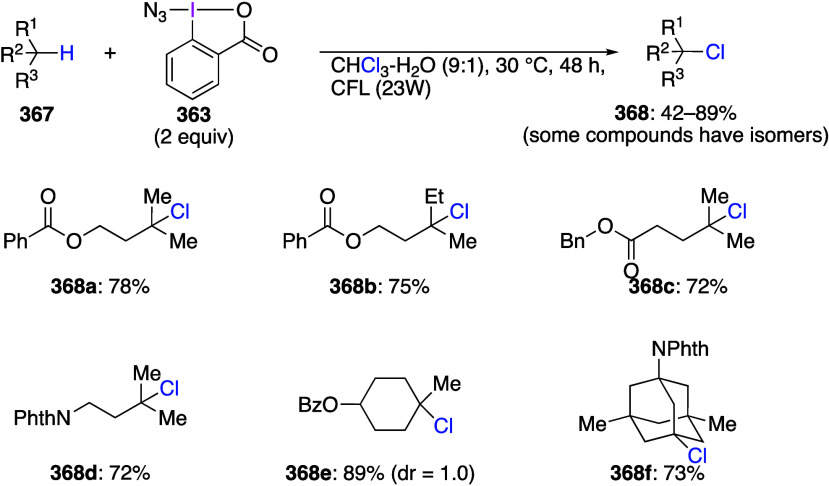
Selective C–H Chlorination of Tertiary Alkyl Compounds **367**

The azido radical species produced from azidobenziodoxole can also be used in hydrogen abstraction reactions of various substrates. For example, Chen and Wang reported that the reaction of compound **367** containing a tertiary alkyl group as a substrate with azidobenziodoxole **363** in a chloroform-water mixture under visible light irradiation yields product **368** in which the tertiary C–H is chlorinated (Scheme 132).^1085^ This reaction proceeds even when secondary alkyl groups are used as substrates, but the selectivity is slightly reduced. Bromoform can be used instead of chloroform to obtain the corresponding brominated compounds. In this reaction, light irradiation generates active azido radicals, which selectively abstract hydrogen from the substrate, producing carbon radical species, followed by chlorine or bromine abstraction from chloroform or bromoform to give final products.

#### 4.3.2 Amido-, Amino-, and Iminobenziodoxoles

**Scheme 133:**
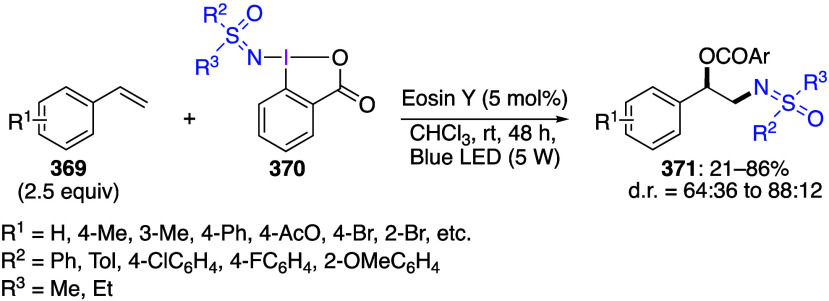
Bifunctionalization of Styrenes **369** Using 1-Sulfoximidoyl-1,2-benziodoxoles **370**

Numerous cyclic hypervalent iodine compounds with the amino, amido, or similar nitrogen functional groups as ligands have been synthesized, and their structures have been established by X-ray crystallography.^697,1086−1091^ Many of these compounds can be used as efficient nitrogen functional group transfer reagents to various substrates.^7^ For example, Bolm and co-workers reported that the reaction of styrenes **369** with 1-sulfoximidoyl-1,2-benziodoxoles **370** under blue LED irradiation in the presence of Eosin Y as a photocatalyst efficiently gave the bifunctionalized products **371** (Scheme 133).^1092^ The authors suggest that the hydrogen bonding ability of the phenolic hydroxyl and carboxyl moieties of the photocatalyst Eosin Y influences the intermediates involved in this reaction as well as the stereochemistry of the products. Hydrogen bonding is important in this reaction, and the reactions in the presence of Ru- or Ir-based photocatalysts do not show any significant diastereoselectivity.

**Scheme 134:**
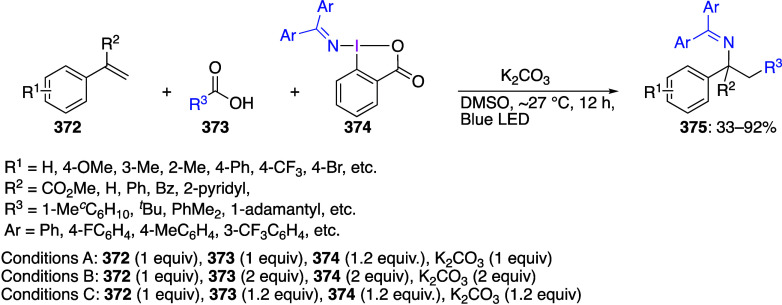
Alkylamination of Styrenes **372**

Kiyokawa and Minakata reported several reactions using benziodoxoles with (diarylmethylene)amino groups as ligands.^1090,1093,1094^ For example, in the reaction of styrenes **372** and carboxylic acids **373** with reagents **374** under basic conditions with blue LED irradiation, a bifunctional addition proceeds to give products **375** (Scheme 134).^1093^ This reaction proceeds via initial formation of diphenyl iminyl radical and *ortho*-iodobenzoyloxy radical generated from the iodine(III) reagent by blue LED irradiation.

**Scheme 135:**
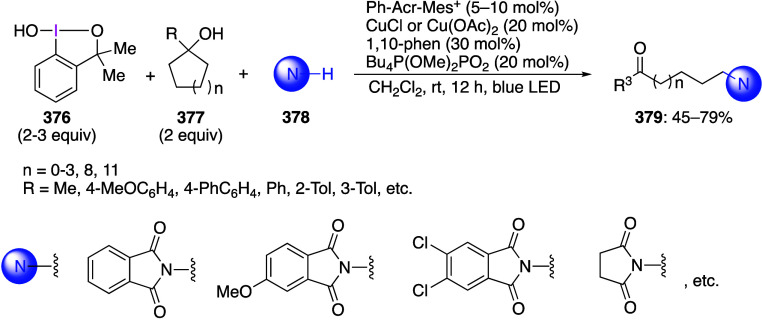
Preparation of Aminoketones **379** from Cyclic Alcohols **376** with Imides **378**

A reaction involving the combination of a benziodoxole and imido compounds to generate an I–N bonded iodine(III) reagent in situ has been reported. Xue and Chen’s group reported that the reaction of dimethylbenziodoxole **376** with cyclic alcohols **377** and imides **378** in the presence of a copper catalyst, a photocatalyst, and catalytic amounts of ligands under blue LED irradiation gave the corresponding imidoketones **379** (Scheme 135).^1086^ The authors detected the intermediate alkoxy iodine(III) active species in this reaction formed from dimethylbenziodoxole and alcohols and the iodine(III) species with I–N bonds from dimethylbenziodoxole and amino compounds by ^1^H NMR. The structure of one of the iodine(III) compounds with an I–N bond has been determined by X-ray crystallography. This reaction is applicable to bioactive molecules with complex skeletons such as cholesterol derivatives and Celecoxib as substrates to obtain the corresponding products. The authors have also investigated the mechanism of this reaction by DET calculations as well as UV–vis and fluorescence quenching experiments.

**Scheme 136:**
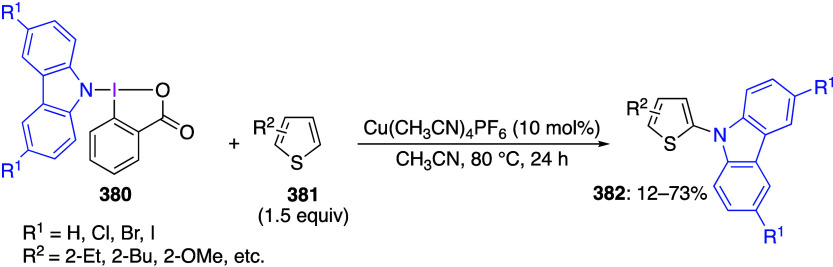
C–H Insertion Amination Reaction of Thiophenes **381**

Cyclic hypervalent iodine reagents with amino ligands are useful reagents for transfer of the amino group to organic substrates.^7^ For example, Liu and Chen reported that the carbazole-substituted benziodoxole **380** reacts with thiophenes **381** in the presence of a copper catalyst, resulting in a regioselective C–H insertion of the carbazole group forming products **382** (Scheme 136).^1089^ The C–H amination reactions of anilines using aminobenziodoxoles with cyclic amino group ligands,^1088^ and α-amination to β-ketoesters using aminobenziodoxoles with acyclic aliphatic amine ligands have also been reported.^1087^

### 4.4 Cyclic Iodine(III) Compounds with Carbon Atom

#### 4.4.1 Trifluoromethylbenziodoxoles and Derivatives

**Scheme 137:**
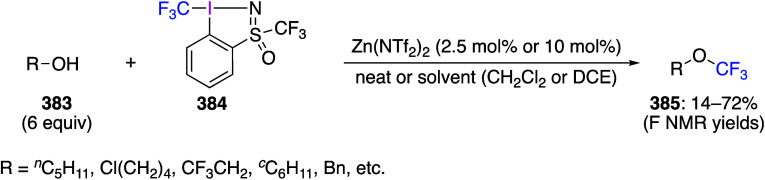
Trifluoromethylation of Alcohols Using Cyclic Iodine(III) Reagent **384**

Trifluoromethylbenziodoxoles and their analogues are cyclic iodine(III) compounds with a trifluoromethyl group as a ligand, which are useful trifluoromethylation reagents that can transfer the trifluoromethyl group to various heteroatoms, unsaturated bonds, and C–H bonds.^27,1095,1096^ Various trifluoromethyl-substituted cyclic iodine(III) compounds were synthesized, and their structures were investigated by X-ray crystallography.^1097−1101^ Trifluoromethylbenziodoxoles or their analogues are commonly used as the reagents for transferring trifluoromethyl group to heteroatoms such as oxygen,^1102−1104^ nitrogen,^1105^ and sulfur^1106−1108^ atoms. For example, Togni’s group reported the trifluoromethylation of alcohols using a cyclic iodine(III) reagent derived from 2-iodosufoximine.^1104^ When alcohols **383** were treated with reagent **384** in the presence of Zn(NTf_2_)_2_, the trifluoromethylation reaction proceeds to form the corresponding trifluoromethyl ether **385** (Scheme 137). When 1-trifluoromethyl-1,2-bendiodoxol-3(1*H*)-one is used in this reaction instead of **384**, the reaction does not proceed, suggesting that electron-withdrawing properties of the sulfoximine fragment in reagent **384** is important. If a mixture of primary and secondary alcohols is used in intermolecular competitive reaction, the trifluoromethylation proceeds in favor of the primary alcohol, and in the reactions of substrates with primary and secondary alcohol moieties in the same molecule, the trifluoromethylation of the primary alcohol occurs.

**Scheme 138:**
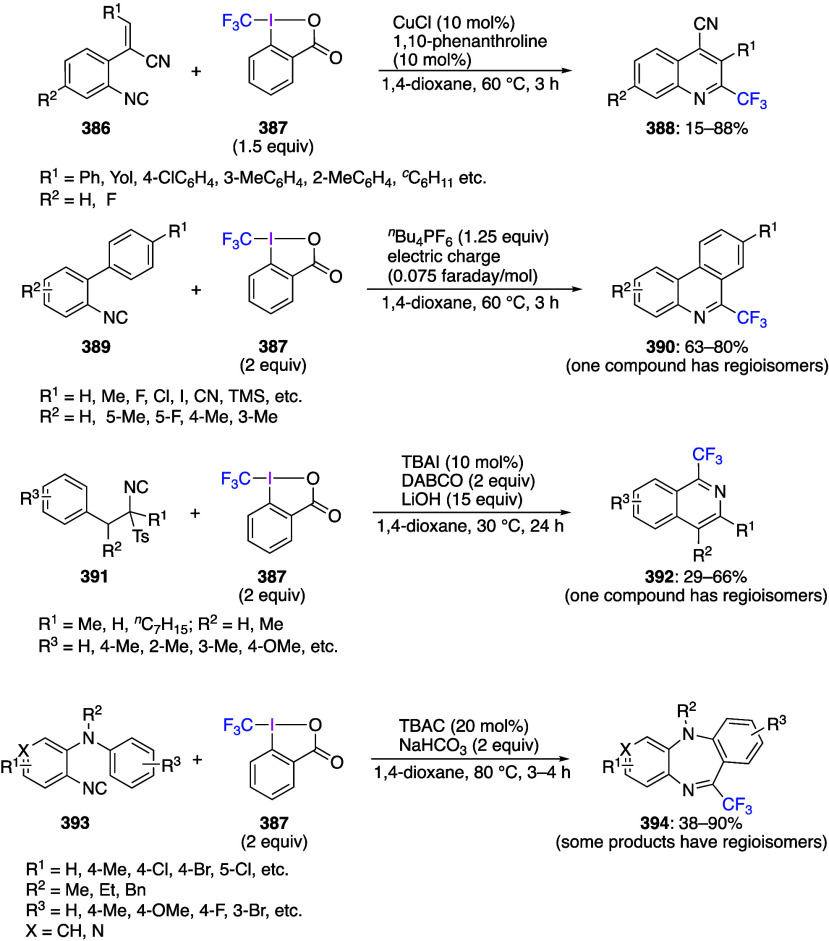
Cyclotrifluoromethylation of Isocyanate Substrates Using **387**

Reactions of some unsaturated substrates with trifluoromethylbenziodoxoles proceed as cyclization-trifluoromethylation, resulting in various cyclic compounds.^1109−1125^ Likewise, the reactions of substrates bearing an isocyanate group lead to the products of cyclization (Scheme 138).^1110,1118,1121,1123^ Wang and Li reported that the reaction of aromatic isocyanate compounds **386** with 1-trifluoromethyl-1,2-benziodoxole-3(1*H*)-one **387** gave trifluoromethylated quinolines **388**.^1118^ Studer’s group found that the reaction of biaryl isocyano compounds **389** with reagent **387** under electrochemical reaction conditions produced quinoline products **390**.^1121^ The reaction of α-benzylated tosylmethylisocyanides **391** yields the corresponding 1-trifluoromethylated isoquinoline derivatives **392**.^1123^ Furthermore, it has been reported that the reaction of *o*-isocyanodiphenylamines **393** as substrates provides trifluoromethylated dibenzodiazepine derivatives **394**.^1110^ In these reactions, trifluoromethyl radical species are generated from the iodine(III) reagent under reaction conditions, which further react with the isocyanate to generate imidoyl radical species, followed by intramolecular cyclization to produce the final products.

**Scheme 139:**
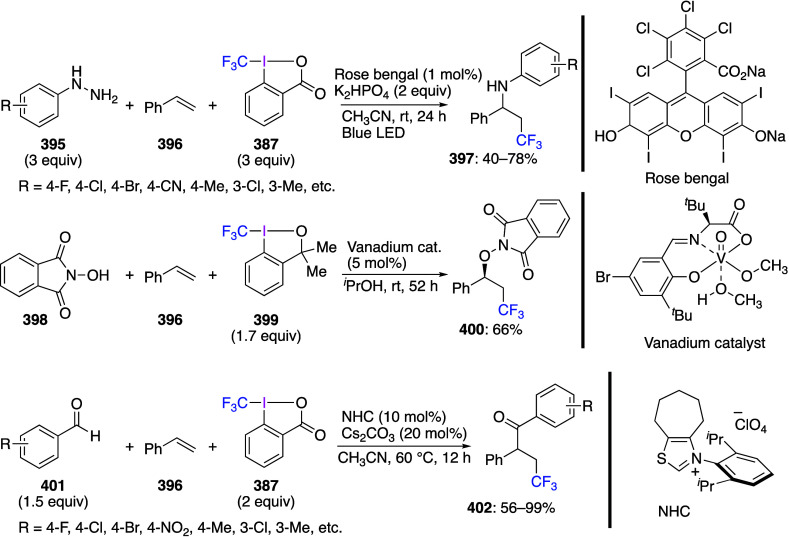
Bifunctionalization of Styrene **396**

Bifunctionalization reactions of unsaturated compounds with reagent **387** or its analogues in the presence of nucleophilic substrates have been reported. Various reactions proceed by the addition of trifluoromethyl radicals generated from reagent **387** to unsaturated bonds, and the radical species containing nitrogen,^1126−1129^ oxygen,^1008,1130−1132^ or carbon^1133−1144^ atoms have been utilized as the second radical species for the bifunctionalization reactions of unsaturated bonds. Bifunctionalization reactions of styrene using trifluoromethylbenziodoxoles have been reported (Scheme 139). For example, the reaction of arylhydrazines **395** with reagent **387** and styrene **396** under blue LED irradiation in the presence of rose bengal as a photocatalyst proceeds as aminotrifluoromethylation of styrene, resulting in the efficient formation of the corresponding amines **397**.^1129^ The addition of radical scavengers to this reaction decreased the yield of products **297**. The reaction progress stops when blue LED irradiation is stopped, which suggests that the reaction probably does not have a radical chain propagation mechanism. Reactions involving 1,2-oxytrifluoromethylation of styrene have also been reported. The reaction of *N*-hydroxyphthalimide **398** and styrene **396** with iodine reagent **399** in the presence of vanadium catalyst results in enantioselective 1,2-oxytrifluoromethylation to give the corresponding bifunctionalized product **400**.^1131^ The reaction proceeds enantioselectively for styrenes with various substituents. The enantioselectivity of this reaction has been analyzed by computational methods. Li and Han have also reported that the reaction of styrene **396** with aromatic aldehydes **401** in the presence of an NHC catalyst results in acylfluoroalkylation of styrene to give the corresponding products **402**.^1138^ The reaction proceeds with various aliphatic alkenes as well as styrenes with various substituents. Even indoles and complex biologically active compounds can be used in place of styrene in this reaction.

**Scheme 140:**
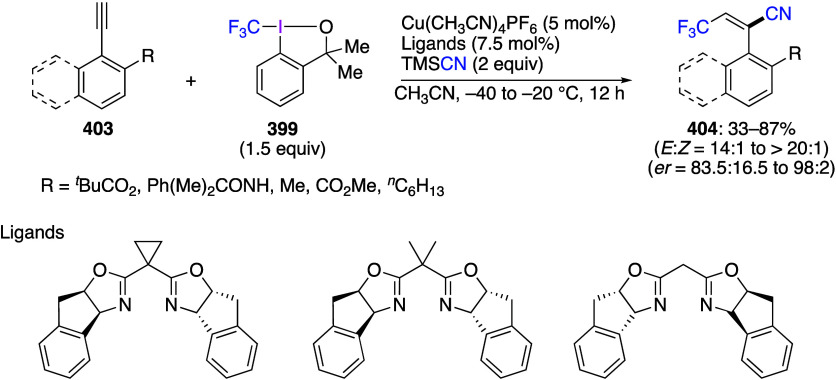
Bifunctionalization of Alkynes **403**

The bifunctionalization reactions are also possible for alkyne derivatives.^1145−1147^ For example, Liu and co-workers reported that reactions of aryl-substituted alkynes **403** with TMSCN and benziodoxole **399** in the presence of a copper catalyst lead to regioselective bifunctionalization, yielding the corresponding alkene derivatives with atroposelectivity (Scheme 140).^1145^ A similar reaction proceeds when TMSN_3_ is used instead of TMSCN to give the corresponding azides. The authors suggest that this reaction has a radical mechanism since adding CBr_4_ to the reaction competitively yields a bromine adduct, and using a radical clock substrate with a cyclopropyl group opens the ring to give the corresponding allene product. The selectivity of the reaction is also explained by DFT calculations.

**Scheme 141:**
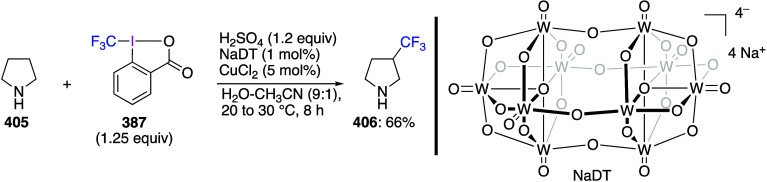
C–H Trifluoromethylation of Pyrrolidine **405**

The cyclic iodine(III) reagents with trifluoromethyl ligand can also be used for the trifluoromethyl group insertion into C–H bonds of various substrates.^1148−1150^ For example, MacMillan and co-workers reported that the reaction of pyrrolidine **405** with trifluoromethylbenziodoxole **387** under Kessi lamp irradiation in the presence of decatungstate and a copper catalyst led to regioselective trifluoromethylation to C–H, resulting in 3-(trifluoromethyl)pyrrolidine **406** in 66% yield (Scheme 141).^1150^ The reaction proceeds regioselectively for various aliphatic amines and benzylic position selectively for benzylic substrates. The authors have confirmed the presence of “Cu-CF_3_” species in various control experiments and suggest that this species may potentially be a catalytic intermediate in the reaction.

**Scheme 142:**
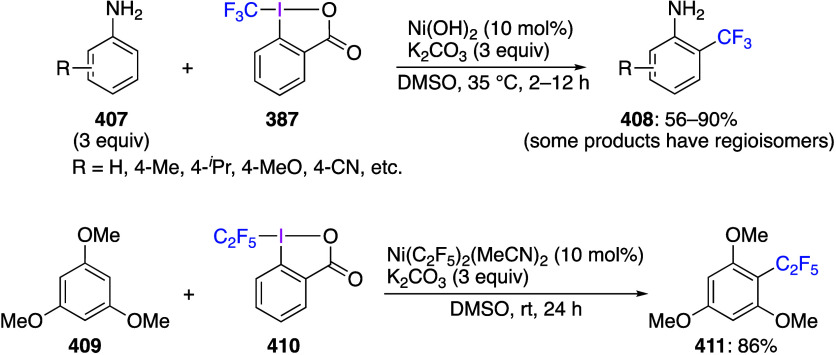
C–H Perfluoroalkylation of Aromatic Compounds **407** and **409**

Trifluoromethylation reactions at the C(*sp*^*2*^)–H bonds have also been reported.^1151−1160^ For example, it was reported that aniline compounds **407** react with trifluoromethylbenziodoxole **387** in the presence of a catalytic amount of Ni(OH)_2_ to give products **408** with trifluoromethyl group in the aromatic ring (Scheme 142).^1154^ This reaction proceeds efficiently with various substituted anilines. Khaskin and co-workers reported that the reaction of 1,3,5-trimethoxybenzene **409** with C_2_F_5_-substituted benziodoxole **410** in the presence of nickel catalyst gave fluoroalkylated product **411** in 86% yield.^1157^ This reaction efficiently proceeds with electron-rich aromatic compounds; however, a decrease in yield is observed for electron-deficient aromatic substrates. In the reactions of C_3_F_7_-substituted benziodoxole as the reagent, the analogous fluoroalkylated product was obtained in similar yields.

#### 4.4.2 Cyanobenziodoxoles

**Scheme 143:**
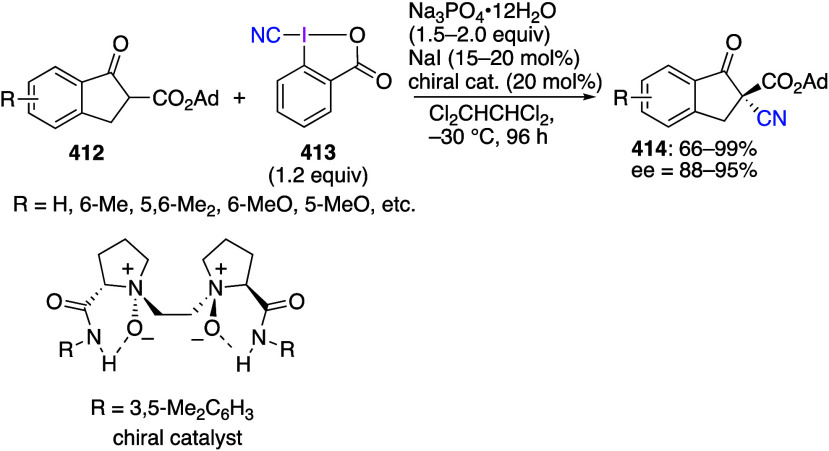
Asymmetric α-Cyanation of β-Keto Esters **412**

Cyclic iodine compounds with a cyano group as a ligand are used as reagents that can introduce a cyano group into various organic substrates.^7,106^ In contrast with the cyclic iodine reagents, the acyclic hypervalent iodine compounds are not effective cyano group transfer reagents. Reactions utilizing cyclic iodine reagents bearing a cyano group can be used to convert the N–H bond to a N–CN bond,^1161,1162^ and the acidic C–H bond to a C–CN bond.^1163,1164^ Feng and Liu reported that the reaction of the β-ketoester 1-indanone derivatives **412** with cyanobenziodoxole **413** in the presence of a chiral catalyst results in enantioselective α-cyanation to afford the corresponding products **414** (Scheme 143).^1164^ A similar enantioselective cyanation reaction proceeds with β-ketoamide compounds as the substrates. The reaction does not provide products of cyanation with acyclic β-ketoester or nonaromatic cyclic β-ketoester as the substrates.

**Scheme 144:**
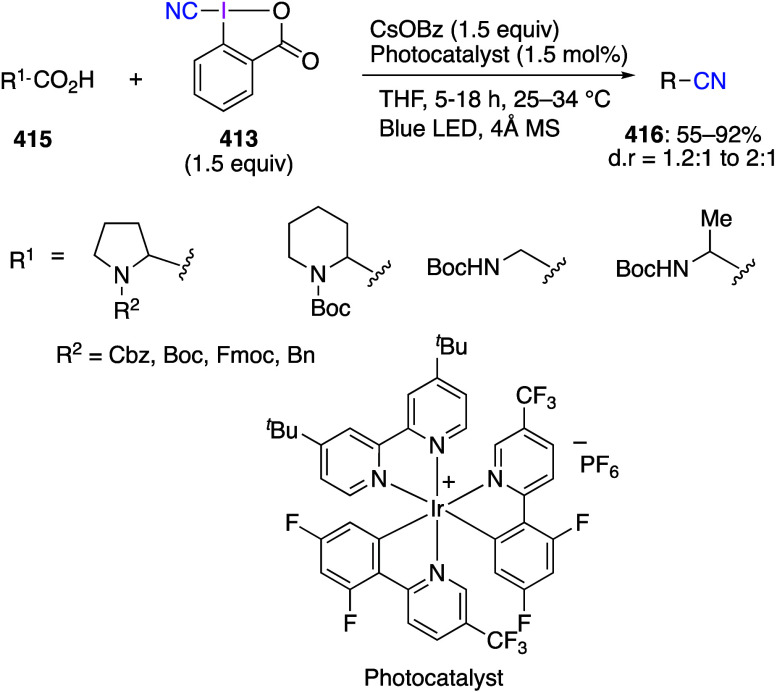
Decarboxylative Cyanation of Carboxylic Acids **415**

Reactions of carboxylic acids or their derivatives with cyanobenziodoxole **413** proceed as a decarboxylation-cyanation process.^1165−1168^ For example, Waser and co-workers reported that the reactions of an aliphatic carboxylic acids **415** with cyanobenziodoxole **413** under blue LED irradiation in the presence of a photocatalyst proceeds to a cyanation reaction via decarboxylation, efficiently yielding the corresponding cyano products **416** (Scheme 144).^1168^ This reaction does not work effectively with the acyclic hypervalent iodine reagents. The authors suggested that this reaction mechanism involves radicals since the reaction in the presence of TEMPO produces less product, while the reaction with a substrate having a cyclopropylmethyl group produces ring-opened products.

**Scheme 145:**
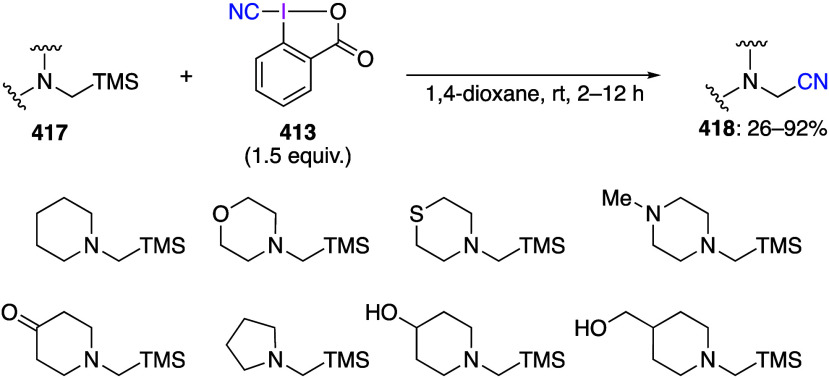
Desilylative Cyanation of Amines **417**

The TMS group can be substituted by the cyano group using cyclic hypervalent iodine reagents. Yuan and co-workers reported that α-silylated tertiary aliphatic amines **417** react with cyanobenziodoxole under blue LED irradiation to give the corresponding cyano compounds (Scheme 145).^1162^ The authors propose the formation of an EDA (electron donor–acceptor) complex between the iodine and the amine as a key intermediate in this reaction. The presence of the EDA complex was confirmed by UV–vis spectroscopy. The 1:1 ratio of iodine(III)/amine in this complex was found by the Job’s method.

#### 4.4.3 Cyclic Diaryliodonium Salts

**Scheme 146:**
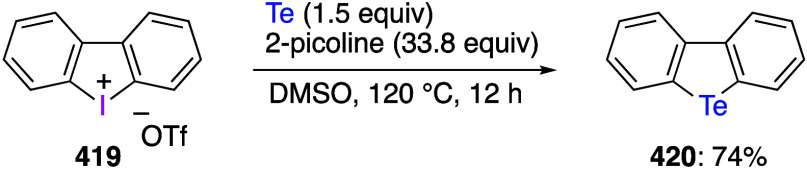
Synthesis of Dibenzotellurophene **420**

The chemistry and synthetic applications of cyclic aryliodonium salts were summarized in a comprehensive review.^161^ Very recently, a number of various polycyclic diaryliodonium compounds have been synthesized and investigated.^101,161,1169^ Similar to the acyclic diaryliodonium salts, these compounds have a relatively high stability. The iodonium atom in cyclic diaryliodonium salts can be substituted with various nucleophiles, forming new heterocyclic or carbocyclic rings, which has been utilized in the synthesis of the corresponding heterocyclic,^1170−1177^ triphenylene,^1178−1182^ and spirocyclic compounds.^1183,1184^ Cyclic diaryliodonium salts can also react with various nucleophiles to give the ring-opening products.^1185−1190^ Tan and Xu reported that the reaction of cyclic diaryliodonium salt **419** with tellurium powder in the presence of 2-picoline as a base gives dibenzotellurophene **420** in high yield (Scheme 146).^1172^ Cyclic diaryliodonium salts with various substituents can be used as substrates in this reaction. The authors have also found that tritellurasumanene can be synthesized from the corresponding cyclic diaryliodonium salt by using this methodology.

**Scheme 147:**
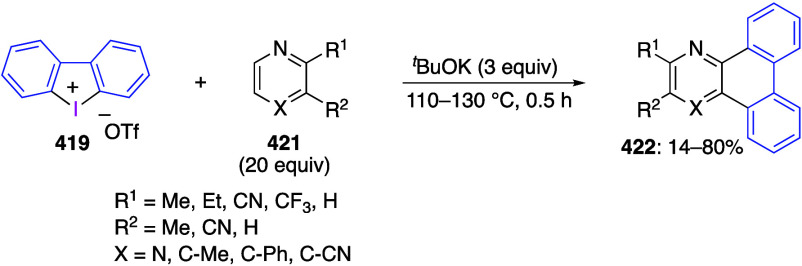
Preparation of Polycyclic *N*-Heteroarenes **422**

Rohde and Hong reported that the reaction of cyclic diaryliodonium salt **419** with unactivated heterocycles **421** under basic conditions yields the corresponding polycyclic heteroarenes **421** (Scheme 147).^1182^ In this reaction, the corresponding products could be efficiently obtained using substituted cyclic diaryliodonium salts and benzene instead of heterocycles **421**. Addition of TEMPO resulted in lower yields of the product, and the TEMPO adduct was detected by ESI-MS, leading the authors to suggest that radicals are involved in the reaction. In a competitive reaction involving a heteroaromatic compound mixed with its deuterated analogue, the kinetic isotope effect is close to 1, indicating that the deprotonation of the C–H of the heteroaromatic ring is not a rate- determining step.

**Scheme 148:**
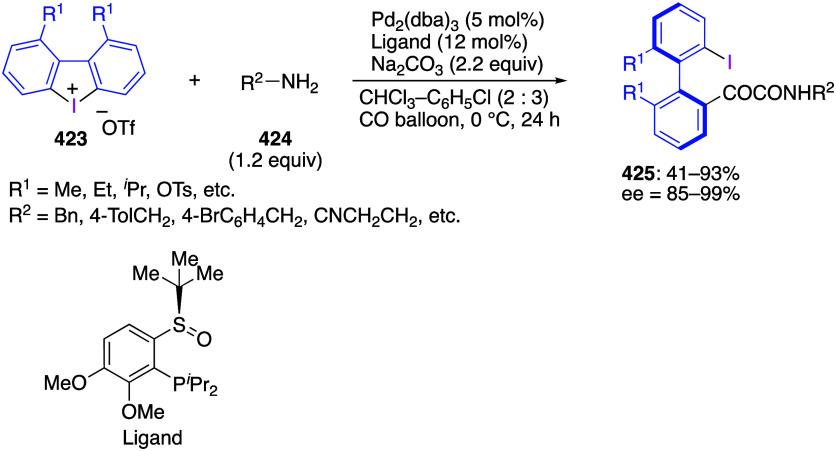
Palladium-Catalyzed Enantioselective Double Carbonylation

As an example of ring-opening reactions of cyclic diaryliodonium compounds **423**, Wu and Liao reported that the reaction with amines **424** in the presence of a catalytic amount of palladium and a ligand under a carbon monoxide atmosphere leads to the enantioselective double carbonylation, yielding the corresponding ketoamides **425** (Scheme 148).^1185^ In the reaction using aromatic amines instead of aliphatic amines, the monocarbonylation reaction proceeds and the corresponding amides are selectively produced. The authors evaluated the mechanism of these reactions by DET calculations. Active palladium(IV) species generated by the action of iodonium salt were detected by HRMS and ^31^P NMR.

**Scheme 149:**
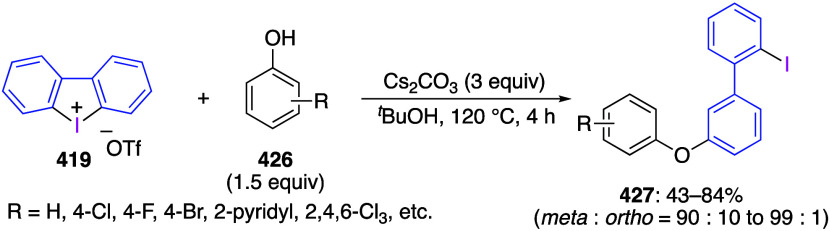
*meta*-Selective O-Arylation of Cyclic Iodonium Salt **419**

Similar to their acyclic analogues, cyclic diaryliodonium salts can produce benzyne species under basic conditions, which has been utilized in several recent works.^1191,1192^ Zhang and Wu reported that the reaction of cyclic iodonium salt **419** with phenolic compounds **426** in the presence of cesium carbonate as a base lead to a *meta*-selective *o*-arylation reaction, resulting in the efficient formation of the corresponding diaryl ethers **427** (Scheme 149).^1191^ The same research group has also reported a reaction using tetrabutylammonium halide instead of a phenol compound as a nucleophile, in which a *meta*-selective halogenation also proceeds, resulting in the corresponding dihalobiaryl products.^1192^ In the presence of furan instead of phenol or halide, the corresponding cycloadduct was obtained, supporting the generation of benzyne intermediates in this reaction.

#### 4.4.4 Alkynyl- and Alkenylbenziodoxoles

**Scheme 150:**
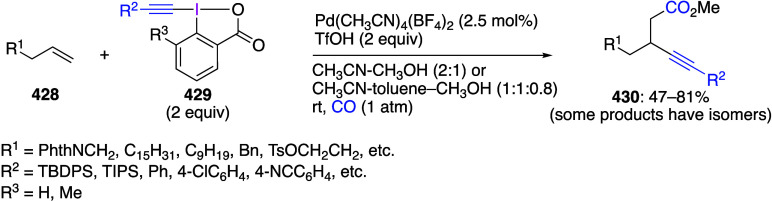
Alkynylcarbonylation of Alkenes **428**

The chemistry and synthetic applications of alkynyl- and vinylbenziodoxoles were summarized in recent comprehensive reviews.^13,130,172^ Cyclic hypervalent iodine derivatives with alkynyl ligands can be synthesized by ligand exchange reactions of the approporiate cyclic hypervalent iodine reagents with silylated or boronated alkynes. Recently, numerous new alkynyl derivatives of benziodoxole or benziodazole have been synthesized and structurally investigated by X-ray crystallography.^1193−1198^ These cyclic alkynyl iodine(III) derivatives have been used in a number of reactions as useful reagents for transfer of the alkynyl group to a variety of substrates.^106,1199,1200^ In particular, these reagents can be used for alkynylation of unsaturated substrates such as alkenes^1201−1205^ and alkynes.^1206,1207^ Chen and Liu reported that the reaction of aliphatic alkenes **428** with alkynylbenziodoxoles **429** under a carbon monoxide atmosphere in the presence of a catalytic amount of palladium proceeds as alkynylcarbonylation to form the corresponding esters **430** (Scheme 150).^1205^ The resulting compounds can be converted to a variety of heterocyclic products by appropriate treatment. The same group also reported that hydrogen-alkynation of alkenes can be achieved by a related reaction using (*n*-hex)_3_SiH as the hydrogen source instead of carbon monoxide.^1201^

**Scheme 151:**
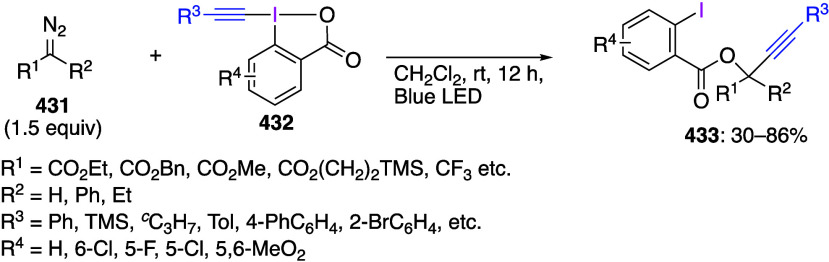
Synthesis of Propargylic Esters **433** from Diazo Compounds **431**

Reaction of diazo compounds **431** with alkynylbenziodoxoles **432** under blue light irradiation proceeds as oxy-alkynylation, yielding propargylic esters **433** (Scheme 151).^1208^ The authors assume that radicals are not involved in the mechanism of this reaction since the addition of TEMPO or BHT does not decrease the product yield. They suggested that this reaction may involve singlet carbenes generated from diazo compounds under blue LED conditions. It is also known that the oxy-alkynylation of diazo compounds to the corresponding propargylic esters can be achieved by the reaction with alkynylbenziodoxoles in the presence of a copper catalyst.^1209,1210^ Alcohols or amines can be introduced in this reaction instead of 2-iodobenzoic acid.^1211,1212^

**Scheme 152:**
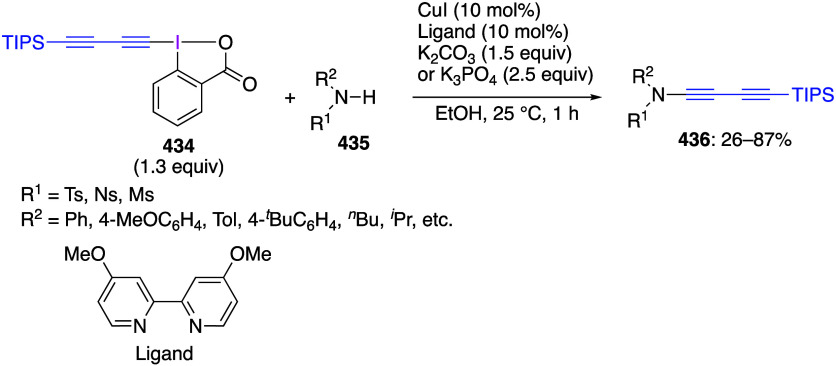
Preparation of Diynamides **436** from Amines **435** Using Reagent **434**

Alkynylbenziodoxoles have been used as alkynylating reagents toward heteroatoms as well as various C–H bonds.^1213−1221^ Furthermore, alkynylation-decarboxylation reactions of carboxylic acids in the presence of photocatalysts have been reported.^1165,1222−1225^ These reactions have been summarized in recent review articles.^13,130^ Recently, Tada and Ito have synthesized cyclic iodine(III) reagents with diyne ligands.^1226^ They reported that the reaction of reagent **434** with amines **435** in the presence of a copper catalyst and a ligand gave the corresponding diynamides **436** (Scheme 152). The resulting diynamide product can be further converted to ynamides via CuAAC (copper-catalyzed azide–alkyne cycloaddition) by appropriate treatment.

**Scheme 153:**
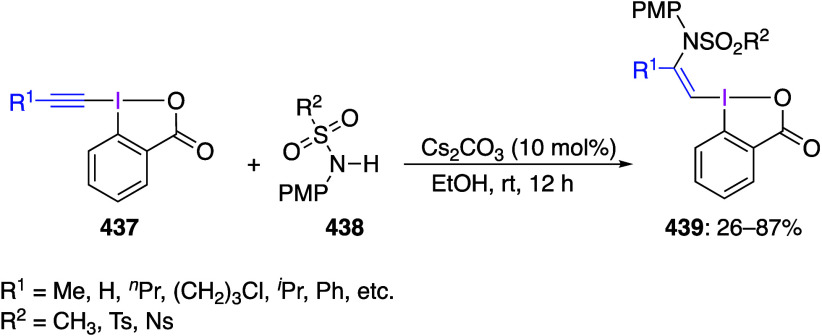
Synthesis of Alkenylbenziodoxoles **439** from Alkynylbenziodoxoles **437**

It has been reported that cyclic alkynyl iodine(III) compounds can be converted to cyclic alkenyl iodine(III) compounds by reaction with various nucleophiles.^172,1227−1235^ For example, Waser and co-workers reported that the reaction of alkynylbenziodoxoles **437** with sulfonamides **438** under basic conditions proceeds stereoselectively, resulting in *Z*-alkenylbenziodoxoles **439** (Scheme 153).^1233^ Phenolic compounds can also be used as nucleophiles in this reaction. The role of the base is to prevent the formation of the ynamide, which is a thermodynamically favorable by-product. In general, cyclic alkenyl iodine(III) compounds can be synthesized by two approaches: (1) by the in situ reaction of 2-iodobenzoic acids with an oxidant and then reacting with an alkene or alkyne followed by base treatment,^1236,1237^ or (2) by reacting the cyclic iodine(III) reagents with alkynes.^1238−1241^

**Scheme 154:**
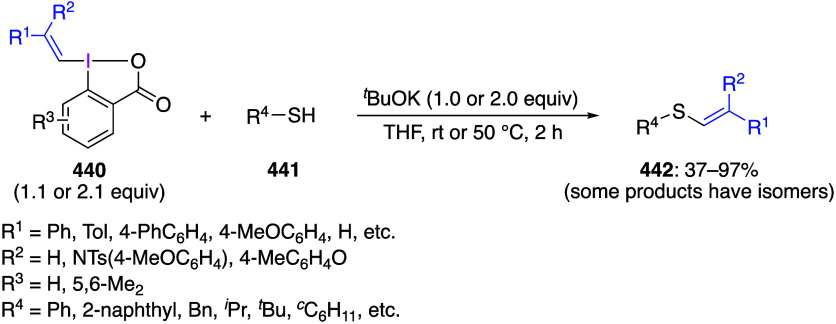
Alkenylation of Thiols **441** Using Alkenylbenziodoxoles **440**

Alkenylbenziodoxoles are used as reagents that can transfer alkenyl groups to various substrates.^1242−1248^ For example, Olofsson and co-workers reported that the reaction of alkenylbenziodoxoles **440** with thiols **441** proceeds stereoselectively under basic conditions to give *E*-alkenylthioethers **442** (Scheme 154).^1246^ Mercaptothiazoles can also be used as nucleophiles in this reaction.

## 5 Pseudocyclic Hypervalent Iodine(III) Reagents

**Scheme 155:**
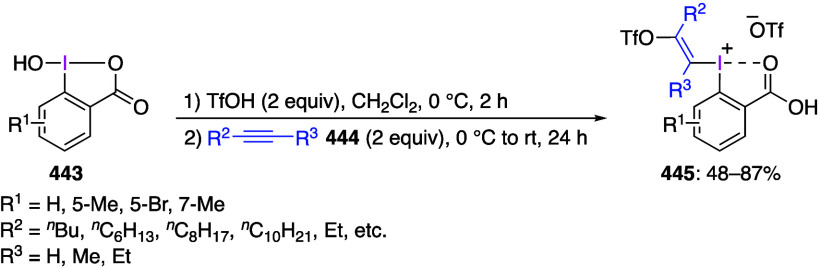
Preparation of Pseudocyclic Alkenyliodonium Triflates **445**

Hypervalent iodine compounds with appropriate substituents in the *ortho*-position of the aromatic ring have a pseudocyclic structure due to the intramolecular noncovalent interaction between iodine and the neighboring group. The presence of additional intramolecular coordination at the iodine(III) center leads to a significant improvement of solubility and stability, as well as to modified reactivity of a hypervalent iodine reagent.^1249^ Synthetic applications of pseudocyclic hypervalent iodine compounds were summarized in a comprehensive review.^114^ The coordinating group in the *ortho*-position of pseudocyclic hypervalent iodine compounds can contain oxygen atoms,^639,640,750,751,991,1250−1255^ such as carbonyl, ether, and hydroxyl groups, or nitrogen-containing functional groups.^1256−1258^ Two general methods for the synthesis of common pseudocyclic hypervalent iodine(III) reagents are known. The first method is to add acid to a cyclic hypervalent iodine(III) compound in order to open the ring and create a coordinating functional group. The second method is by the oxidation of iodoarenes with coordinating functional groups in the *ortho*-position. The structure of numerous pseudocyclic hypervalent iodine compounds has been confirmed by X-ray crystallography.^640,750,991,1250,1252−1258^ For example, the addition of TfOH to hydroxybenziodoxoles **443**, followed by the addition of alkynes **444**, efficiently synthesizes the corresponding pseudocyclic β-trifluorosulfonyloxy alkenyliodonium salts **445** (Scheme 155).^1250^ These products can also be obtained by adding Tf_2_O instead of TfOH.^1259^ The structure of compounds **445** was determined by X-ray crystallography. The obtained pseudocyclic compounds **445** react with sodium azide in the presence of crown ether by the addition-elimination mechanism with retention of configuration, resulting in the alkenylbenziodoxoles with an azide group at the β-position.

**Scheme 156:**
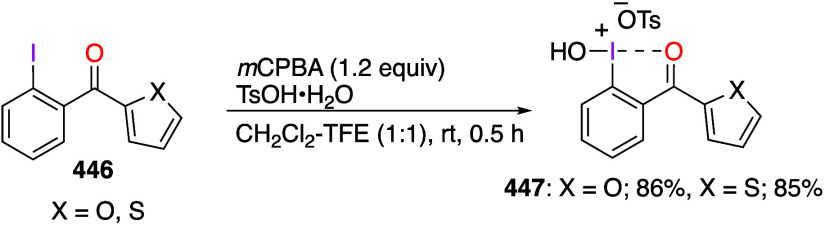
Synthesis of Pseudocyclic Iodine(III) Compounds **447**

Wirth and co-workers reported the synthesis of pseudocyclic hypervalent iodine compounds **447** by the oxidation of iodoarenes **446** with a heteroaryl carbonyl group at the *ortho*-position of the iodine atom (Scheme 156).^1255^ The structure of these compounds was confirmed by X-ray crystallography. The authors have demostrated that compounds **447** can be used as oxidants for various substrates. Compared to the analogous noncyclic iodine(III) reagent, [hydroxy(tosyloxy)iodo]benzene (HTIB), compounds **447** were generally more reactive in these reactions. In contrast, catalytic reactions using iodoarenes **446** as organocatalysts did not proceed efficiently.

**Scheme 157:**
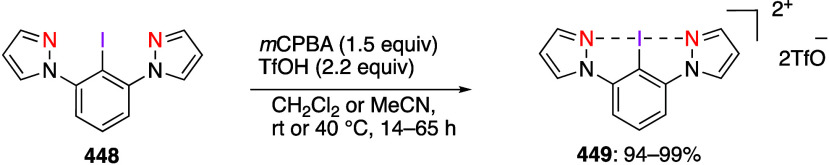
Synthesis of Pseudocyclic Iodine(III) Compound **449** with 1*H*-Pyrazole Rings

Nachtsheim and co-workers prepared pseudocyclic iodonium compound **449** by oxidation of iodobenzene **448** with 1*H*-pyrazole ring substituents at both *ortho*-positions (Scheme 157).^1258^ The authors have also synthesized pseudocyclic compounds from iodoarenes with *ortho*-substituents other than pyrazole *N*-heterocycles, such as triazoles, benzimidazoles, and benzoxazoles. Compound **449** was found to perform as a useful oxidant for a variety of substrates. Interestingly, structure **449** resembles a pincer complex typical of transition metal complexes.

**Scheme 158:**
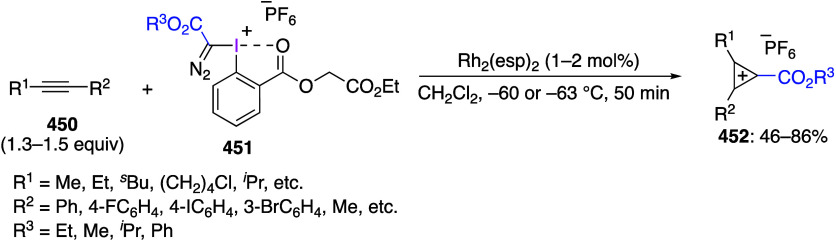
Preparation of Cyclopropenium Salts **452** from Alkynes **450**

Suero and co-workers found that reactions of alkynes **450** with pseudocyclic iodine reagents **451** acting as a carbyne source in the presence of rhodium catalyst produce cyclopropenium salts **452** (Scheme 158).^1260^ This reaction can be performd only by using pseudocyclic iodine reagents; the analogous reactions with cyclic or noncyclic iodine(III) reagents do not give products **452**. The authors suggest that this reaction mechanism involves initial formation of rhodium-carbynoid species, which reacts with an alkyne to yield final products. The resulting cyclopropenium salts **452** can be further reacted with appropriate nucleophiles to obtain the corresponding cyclopropenes as a single regioisomer.

**Scheme 159:**
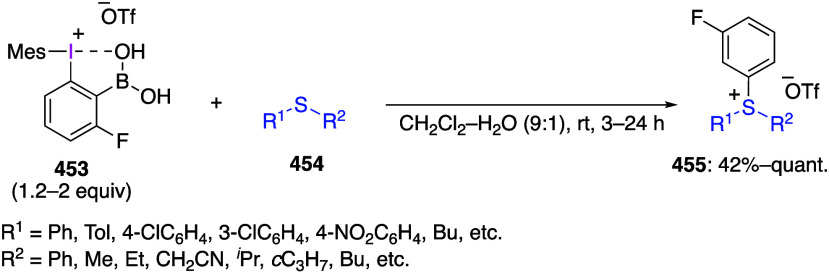
Synthesis of Arylsulfonium Triflates **455** from Sulfides **454**

Pseudocyclic diaryliodonium compounds with a boronic acid group at the *ortho*-position of the iodine atom are unique reagents for generating aryne species under aqueous conditions, which can further react with various substrates to yield the corresponding products.^100,991^ Very recently, it was reported that the reaction of a pseudocyclic diaryliodonium compound **453** with sulfur compounds **454** under aqueous conditions at room temperature yields the corresponding sulfonium salts **455** in high yield (Scheme 159).^1261^ Using sulfoxides instead of sulfides as substrates in this reaction leads to *ortho*-hydroxysulfonium compounds.

## 6 Catalytic Cycles Based on Iodine(III) Species

**Scheme 160:**
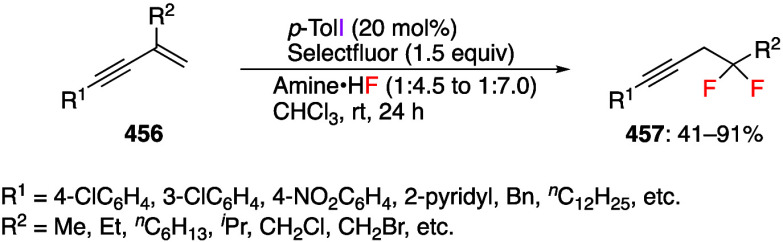
Catalytic Difluorination of Enynes **456**

Catalytic application of hypervalent iodine species is a hot topic in modern synthetic organic chemistry. An excellent book “Iodine Catalysis in Organic Synthesis” edited by Ishihara and Muñiz was published in 2022.^4^ Several recent reviews on this topic were also published.^28,29,118,1262,1263^ This section will provide an update on important development in this area published after 2021. Various catalytic reactions employing hypervalent iodine species generated from catalytic amounts of iodoarenes and stoichiometric oxidants have been reported since 2005.^1264,1265^ Numerous new iodoarenes were designed and utilized as organocatalysts under different reaction conditions. It should be noted that most of these reactions require a relatively large amount of iodoarene, up to 20–30%, which makes them different from the truly catalytic reactions of transition metals. *m*CPBA, Selectfluor, and Oxone are most commonly used as terminal oxidants to generate hypervalent iodine species from iodoarenes in these reactions.^28^ In recent years, reactions using oxygen as the terminal oxidant^127,1266,1267^ and electrochemical reactions have also been developed.^1268−1271^ Gilmor and co-workers have reported the reaction of different iodoarenes with Selectfluor as the oxidant to generate the corresponding aryliodofluoronium species utilized in various catalytic fluorination reactions.^1272−1276^ For example, enynes **456** react with Selectfluor (F-TEDA-BF_4_), and amine·HF in the presence of catalytic amounts of 4-iodotoluene to yield products of difluorination of the double bond (Scheme 160).^1272^ The resulting products **457** can be further converted to a variety of compounds by appropriate treatment. The authors propose that the initially generated hypervalent iodine(III) species in this system react with the double bond followed by alkynyl group rearrangement to give final products **457**. The same group also reported a difluorination reaction that utilizes an alkyl group shift instead of an alkynyl shift.^1277^

**Scheme 161:**
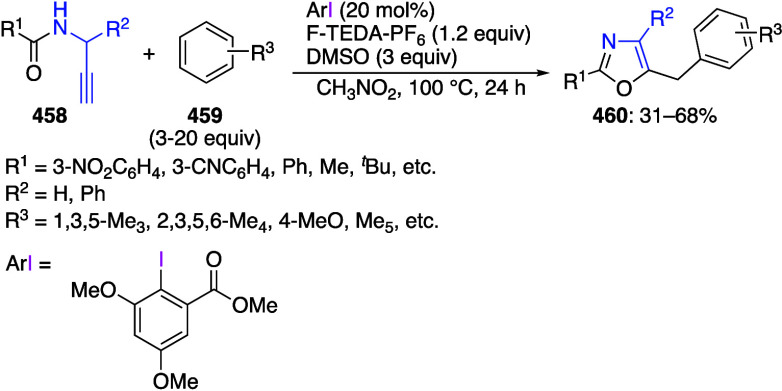
Cycloisomerization-Arylation of *N*-Propargyl Amides **458**

Saito’s group reported the cycloisomerization-arylation of propargylic amides using F-TEDA-PF_6_, an analogue of Selectfluor, as a terminal oxidant with catalytic amounts of iodoarene in the presence of arenes.^1278^ The reaction of propargylamides **458** with electron-rich arenes **459** and the oxidant F-TEDA-PF_6_ in the presence of catalytic iodoarene and DMSO affords oxazole compounds **460** (Scheme 161). The authors suggest a role for DMSO in the generation of sulfinyl fluoride active species (MeSOF) from DMSO and F-TEDA-PF_6_ and confirmed the presence of these species by ^1^H NMR. The same group has also reported the reaction using Bn_2_SO instead of DMSO and confirmed the presence of BnSOF active species by NMR.^1279^ More recently, the authors have succeeded in developing a catalytic system combining TFDA-PF_6_ with Bn_2_SO and NsCl, which was utilized in the synthesis of furans from 2-propargyl-1,3-dicarbonyl compounds.^1280^

**Scheme 162:**
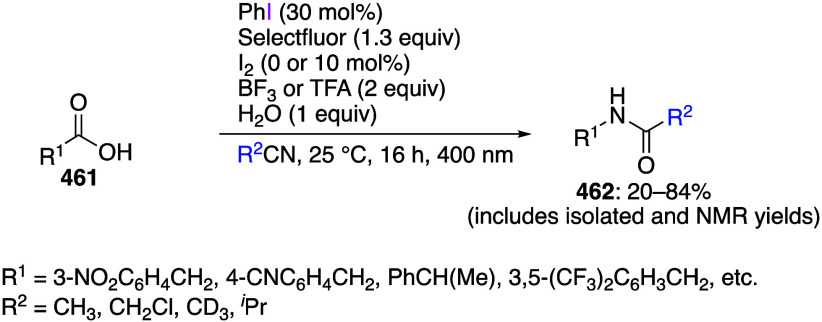
Decarboxylative Amination of Carboxylic Acids **461**

König and co-workers reported catalytic reactions utilizing the generation of the corresponding (diacyloxyiodo)arenes from iodoarenes and carboxylic acids and Selectfluor.^1281,1282^ For example, carboxylic acids **461** under light irradiation in the presence of catalytic amounts of iodobenzene and stoichiometric Selectfluor in the presence of water and BF_3_ experience a Ritter-type reaction involving decarboxylation and resulting in the corresponding amides **462** (Scheme 162).^1281^ Benzyl carboxylic acids and aliphatic carboxylic acids with primary, secondary, or tertiary carbon atoms can be used as substrates in this reaction. The authors assume that (diacyloxyiodo)arene is initially generated in the reaction system, which is then photolyzed to cleave the acyloxy groups and undergo decarboxylation to generate radical species, followed by conversion to cationic species, and finally trapping by nitrile to form final amide. The same authors have also reported a Ritter-type amination of benzylic compounds using radical species generated from (diacyloxyiodo)arene under similar reaction conditions.^1282^

**Scheme 163:**
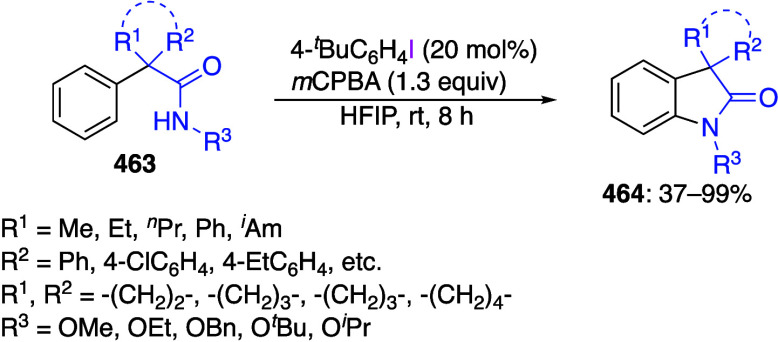
Hypervalent Iodine-Mediated Intramolecular Cyclization of **463**

*m*CPBA and iodoarenes are a classic and the most common combination for generation of hypervalent iodine species in the reaction system.^28,1283^ For example, when a catalytic amount of iodoarene and *m*CPBA as a terminal oxidant are added to phenylpropanamides **463**, an intramolecular cyclization reaction proceeds, resulting in oxyindole compounds **464** (Scheme 163).^1284^ This reaction in the presence of a chiral iodoarene yields the corresponding products with moderate enantioselectivity. The intramolecular cyclization in this reaction proceeds preferentially at the C–N bond rather than the C–O bond. On the other hand, when a substituent is introduced into the aromatic ring of the phenylpropanamide substrate, a competitive reaction involving the C–O and C–N bonds occurs, yielding two products.

**Scheme 164:**
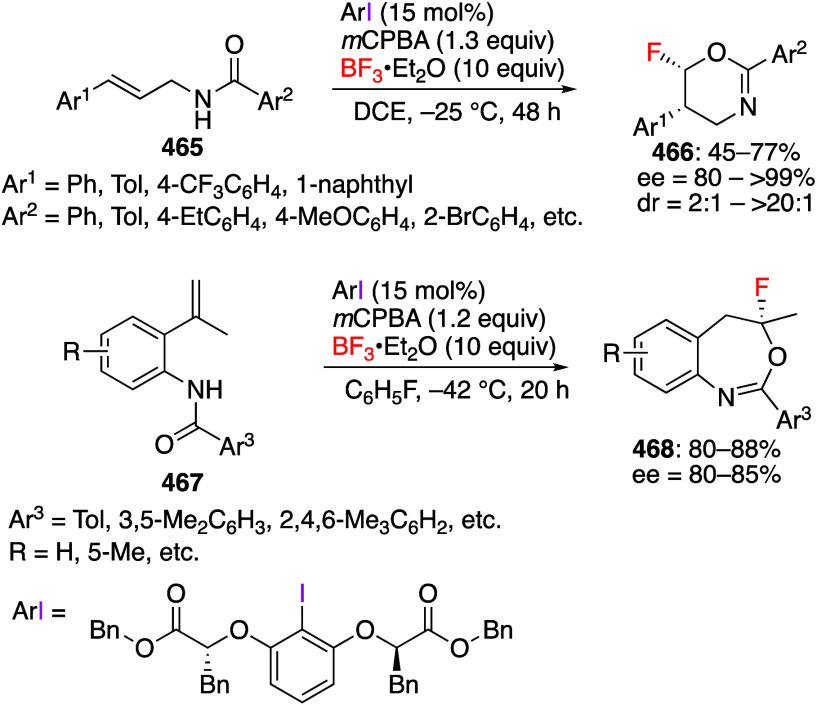
Catalytic Intramolecular Cyclofluorination of Benzamides **465** and **467**

Jiang and co-workers reported enantioselective cycloamino fluorination of cinnamylbenzamide using BF_3_ as a fluorine source.^1285^ The reaction using cinnamylbenzamides **465**, chiral iodoarene, *m*CPBA, and BF_3_ yields the 6-membered ring products **466**, while with the use of benzamides **467**, the corresponding seven-membered ring products **468** are obtained (Scheme 164). The authors demonstrated that the reaction is substrate-specific, as the desired product is not produced when the length of the alkyl group is extended or when the aromatic ring is removed from the substrate. In the reactions using Py–HF or Et_3_N–HF instead of BF_3_, and in the reactions using PhIF_2_, no desired product was obtained, which suggests that BF_3_ is not only a fluorine source but is also an activator of the hypervalent iodine species produced in the reaction system. The authors have also investigated mechanisms of these reactions using computational methods.

**Scheme 165:**
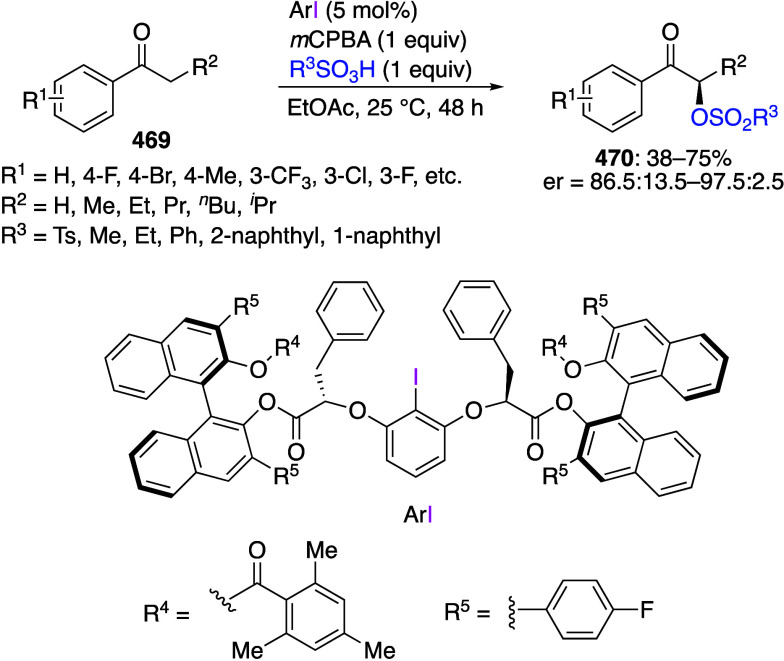
Catalytic α-Tosyloxylation of Ketones **469**

Zhang and co-workers have demonstrated that the reaction of various aryl carbonyl compounds **469** with chiral iodoarenes in the presence of *m*CPBA and TsOH proceeds as enantioselective tosyloxylation, yielding the corresponding α-tosyloxy compounds **470** (Scheme 165).^1286^ The iodoarene used in this reaction is designed to introduce two sterically demanding BINOL functional groups around the iodine, thereby immobilizing the reaction field and preventing free rotation of the substituent. This catalytic design can also be used for intramolecular cyclization reactions. When 5-oxo-5-arylpentanoic acids are used as a substrate in the presence of TFA, the reaction proceeds enantioselectively as a lactonization reaction to afford the corresponding lactones. Wirth group has also reported the synthesis of chiral iodotriptycenes and their use for catalytic enantioselective α-tosyloxylation of propiophenone.^1287^

**Scheme 166:**
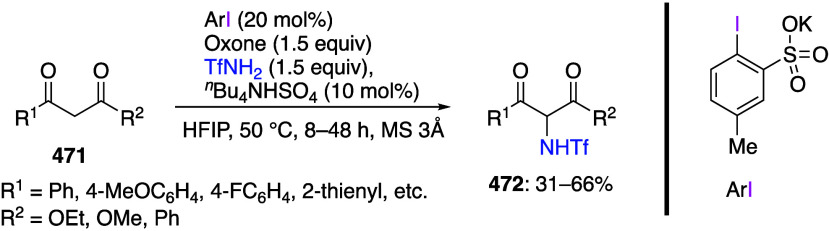
α-Amidation of 1,3-Dicarbonyl Compounds **471**

As an example of a recent catalytic reaction using Oxone as an oxidant, Saito and co-workers reported the α-amidation of diketone compounds with iminoiodane species.^1288^ This reaction was performed by the addition of TfNH_2_ and Oxone to 1,3-dicarbonyl compounds **471** in the presence of iodoarene, giving α-amide products **472** in moderate yield (Scheme 166). This catalytic reaction does not proceed with 1,3-carbonyl compounds bearing sterically bulky substituents. The authors have also succeeded in generating PhINTf from PhIO and TfNH_2_ and using it to obtain the desired products.

**Scheme 167:**
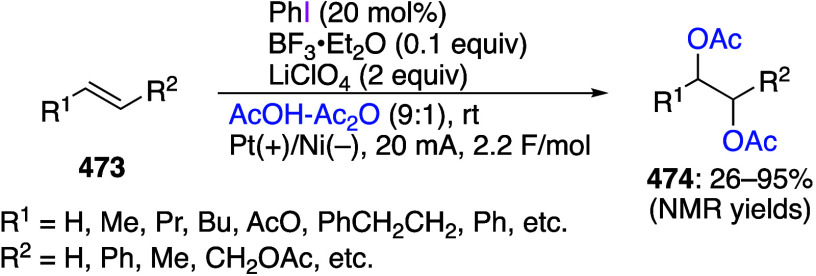
Electrocatalytic Diacetoxylation of Alkenes **473**

De Vos and co-workers reported on the diacetoxylation of alkenes using an electrocatalytic method without the use of organic oxidants.^1268^ The reaction takes place under Pt anode and Ni cathode conditions, and the reaction of alkenes **473**, catalytic amounts of iodobenzene with BF_3_, acetic acid, and acetic anhydride gives the corresponding diacetoxy compounds **474** (Scheme 167). This reaction proceeds even when a carbon rod anode is used instead of the platinum anode. These reaction conditions can also be used for acetoxylactonization. In the reaction of an alkene with a cyclopropyl ring as a substrate, the products of the ring opening were obtained. The authors suggested that a one-electron oxidation mechanism was involved in this reaction.

## 8 Conclusion

The literature summarized in this review reflects an increasing current interest in synthetic applications of hypervalent iodine(III) reagents. Just in 8 years after publication of our 2016 review, numerous new synthetic methodologies based on hypervalent iodine chemistry have been developed and increasingly employed in organic chemistry. Hypervalent iodine reagents are commercially available and environmentally safe compounds with diverse synthetic applications.

Thousands of new research papers dedicated to various uses of hypervalent iodine compounds are published every year. Most of these papers deal with the classic, commercially available reagents, such as (diacetoxyiodo)benzene, (dichloroiodo)benzene, iodosylbenzene, 1-hydroxybenziodoxole, and various iodonium salts. At the same time, the newly deleloped cyclic reagents, including azido-, alkynyl-, and alkenylbenziodoxoles, are attracting significant research activity as the efficient group transfer reagents. Numerous new synthetic methodologies based on hypervalent iodine reagents have been developed recently. Most notably, catalytic and photocatalytic reactions of these compounds are gaining significant interest. Synthetic utilization of new hypervalent iodine reagents and catalytic systems, and the discovery of enantioselective reactions based on chiral hypervalent iodine reagents and catalysts has become an important direction in the field of modern hypervalent iodine chemistry.

Hypervalent iodine reagents and synthetic methodologies involving hypervalent iodine species represent an essential tool of modern organic synthesis. Many synthetic applications of hypervalent iodine compounds are unique and cannot be achieved by using any alternative methods. We anticipate that the development of new reagents and synthetic methodologies based on hypervalent iodine chemistry will continue to be an active area of research in the future.
